# Effect of mobile applications in oral health promotion among elderly people: a systematic review and meta-analysis

**DOI:** 10.1186/s12903-026-08202-z

**Published:** 2026-04-06

**Authors:** Yuanchen Wang, Zhengzhe Zhan, Kaixiao Yan, Jiayi Gui, Xi Guo, Ruirui Zhong, Zeyu Yu, Baotian Zhang, Yutao Xiong, Wei Tang, Chang Liu

**Affiliations:** 1 State Key Laboratory of Oral Diseases & National Center for Stomatology & National Clinical Research Center for Oral Diseases & Department of Oral and Maxillofacial Surgery, West China Hospital of Stomatology, Sichuan University, Chengdu, Sichuan Province 610041 P.R. China; 2 Center for Oral Health, Ruijin Hospital, Shanghai Jiao Tong University School of Medicine, Shanghai, 200025 P.R. China; 3 Department of Oral and Maxillofacial Surgery, University of Michigan School of Dentistry, Ann Arbor, MI 48109 USA; 4 College of Biomedical Engineering, Sichuan University, Chengdu, Sichuan Province 610064 P.R. China

**Keywords:** Mobile applications, Preventive dentistry, Dental care, Dental health education, Elderly people, Systematic review, Evidence based medicine, Meta-analysis

## Abstract

**Objective:**

To evaluate the effects of mobile applications, as reported in randomized controlled trials (RCTs), on oral health prevention and promotion among elderly individuals aged 65 and above.

**Materials and methods:**

Data were sourced from multiple databases, including Medline, Embase and 10 others, as well as ICTRP up to February 2025, without language restrictions. Pairs of reviewers independently selected RCTs, extracted data, appraised risk of bias with RoB-2 and rated certainty (GRADE). Meta-analyses were undertaken wherever appropriate. For outcomes exhibiting high heterogeneity, subgroup analyses were conducted to investigate the sources of the heterogeneity.

**Results:**

From 4375 references, 9 studies met inclusion criteria. Interventions primarily involved asynchronous smartphone applications (*n* = 7) or combined asynchronous/synchronous text messaging (*n* = 2). Compared to conventional strategies, mobile applications demonstrated no effects in gingival index, OHIP-14 and GOHAI. However, versus no intervention, mobile applications improved USFR (SMD, 1.93; 95%CI, 0.85 to 3.01), while reducing OHIP-14 (SMD, -0.82; 95%CI, -1.59 to -0.05) and Winkel tongue coating index (SMD, -1.02; 95%CI, -1.66 to -0.39). Sustained effects over time were also observed.

**Conclusions:**

In comparison to no intervention, mobile applications may have effects on oral health promotion. However, relative to conventional strategies, there had been no significant change in the performance of the mobile application. But current evidence was sparse and low-quality, the results of meta-analyses should be interpreted with caution.

**Trial registration:**

This review was registered in PROSPERO (ID: CRD42024583806) on September 06, 2024. Detailed proposal was available for public access, further details can be found at the following link (https://www.crd.york.ac.uk/PROSPERO/view/CRD42024583806).

**Supplementary Information:**

The online version contains supplementary material available at 10.1186/s12903-026-08202-z.

## Introduction

Over the past decade, global internet usage had demonstrated a significant upward trajectory across diverse populations [1]. This trend became particularly pronounced during the COVID-19 pandemic, wherein digital connectivity emerged as the primary conduit for accessing external information and services. Within this digital ecosystem, mobile applications had established themselves as indispensable platforms for knowledge acquisition across numerous domains [2].

The mobile applications were software programs specifically designed for execution on mobile devices, such as smartphones and tablets, leveraging mobile operating systems (iOS, Android) to deliver targeted functionality [3]. Mobile applications included social media platforms (e.g. WhatsApp, Facebook and Twitter), video platforms (e.g. YouTube, TikTok and BiliBili) and specific applications that provided services to specific groups of people. Technological advances, such as developments of telecommunication, had promoted the widespread and advanced adoption of mobile applications, particularly in medicine [4].

According to epidemiological research findings, the health care cost burden was rapidly increasing, particularly in relation to health issues associated with the elderly population [5]. Older adults face distinct challenges in this landscape, often experiencing constrained access to health knowledge due to mobility limitations, reduced technology familiarity, and constraints of offline health resources [5, 6]. Currently, mobile applications played an important role in knowledge dissemination, information acquisition, and health education [7]. Consequently, mobile applications represented a promising and potentially transformative avenue for delivering health education to elderly populations, thereby supporting proactive health management.

Within the specific domain of oral health, contemporary mobile applications designed to promote oral hygiene had demonstrated efficacy in reducing gingivitis and enhancing preventive behaviors [8, 9]. Specifically, these digital tools function effectively as educational and motivational instruments, facilitating improved plaque control and patient adherence to oral care regimens [10]. Notably, however, existing research predominantly concentrated on adolescent and orthodontic populations [11], leaving a critical gap that the impact of mobile applications on oral health promotion specifically among elderly individuals remains inadequately characterized.

Emerging investigations suggested mobile applications were increasingly being leveraged as educational or self-assessment tools for older adults [12]. Mechanisms underpinning their potential effectiveness include enhancing oral health knowledge of elderly, which subsequently fosters greater awareness of oral care importance and cultivates improved self-management capabilities [13].

While preliminary research offered insights into these potential mechanisms, consensus on the specific quantitative impact of mobile applications on measurable oral health outcomes in the elderly remained elusive. Crucially, no systematic synthesis existed to consolidate available evidence or quantitatively evaluate efficacy via meta-analysis. Addressing this research gap is therefore imperative. Conducting a systematic review and meta-analysis was essential to clarify the precise effects and potential value of mobile applications for enhancing oral health in the ageing population.

## Methods

This review was performed according to the Cochrane Handbook for Systematic Reviews of Interventions [14] and the Preferred Reporting Items for Systematic Reviews and Meta-analysis (PRISMA) 2020 statement [15].

### Research question and eligibility criteria

This systematic review aimed to address the following PICO question regarding the effect of mobile applications for oral health promotion, as compared with conventional strategies or no intervention, in elderly population. The inclusion criteria were guided by the PICOS framework, as follows:

- Population (P): elderly people over 65 years old with certain cognitive ability and self-care ability.
- Intervention (I): any intervention of oral health promotion using mobile applications via smartphones and tablets. Mobile applications include social media platforms (e.g. WhatsApp, Facebook and Twitter), video platforms (e.g. YouTube, TikTok and BiliBili), special applications that send reminders of dental visits, and other applications.
- Comparator (C): any comparator of oral health promotion with conventional strategies, such as lecture-based education, or with no intervention.
- Outcomes (O): oral health-related knowledge, oral health-related daily behaviors, dental treatment adherence and periodontal outcomes/oral hygiene levels.
- Study design (S): randomized controlled trials (RCTs).

Exclusion criteria were (1) people aged less than 65 years; (2) elderly people who have lost cognitive ability or self-care ability (e.g. Alzheimer’s disease); (3) any intervention of oral health promotion not only using mobile applications; (4) nonrandomized studies such as quasi-RCTs, cohort studies, case control studies, and cross-sectional studies; (5) ongoing studies.

### Search strategy

A systematic literature search was conducted across 12 databases to identify both published and unpublished studies up to February 3, 2025: MEDLINE (searched via PubMed), Embase, Cochrane Central Register of Controlled Trials (CENTRAL), Web of Science Core Collection (WoSCC), Korea Citation Index (KCI), Scientific Electronic Library Online (SciELO), Chinese BioMedical Literature Database (CBM), Airiti Library, WHO Global Index Medicus, Open Dissertations, ProQuest Dissertations & Theses (PQDT), and Preprint Citation Index. Furthermore, the World Health Organization International Clinical Trials Registry Platform (ICTRP) was screened for any relevant registered clinical trials up to February 3, 2025. The search strategy imposed no restrictions on the publication language or date. To identify additional pertinent records, citation tracking of eligible studies was also performed. The comprehensive search strategies were detailed in Appendix A.

### Study selection and data collection

An independent screening of the titles and abstracts of the retrieved records was conducted by two reviewers (Z.Z. and Y.W.), after which the full reports for all studies deemed to potentially meet the inclusion criteria were acquired. An independent assessment of the eligibility of all retrieved full reports was conducted by the two reviewers (Z.Z. and Y.W.). Any disagreements that emerged were resolved through discussion or by recourse to another reviewer (C.L.), who acted as an arbiter. Data extraction from the included studies was performed independently by two reviewers (Z.Z. and Y.W.). Any disagreements that arose during this process were resolved through discussion or by recourse to another reviewer (C.L.) acting as an arbiter. When data were unavailable or inapplicable to our review (e.g. the included population contained more than just elderly individuals over 65 years of age, with no separate reporting of results for this age group), we contacted the corresponding authors to obtain the relevant data.

This methodological approach was adapted from previously published studies [16, 17] by our research group, with modifications made to suit the current study objectives.

### Data items

In this review, we defined and extracted the primary outcomes were:

- Oral health-related knowledge: This outcome is measured with self-reported questionnaires.
- Oral health-related daily behaviors: This outcome includes objective or self-reported time and frequency of toothbrushing, mouth rinsing and other oral health-related daily behaviors.
- Dental treatment adherence: This outcome includes appointment attendance (number or proportion of appointment attended, cancelled or late attendance).
- Periodontal outcomes/oral hygiene levels: Clinical assessments of oral hygiene levels include simplified oral hygiene index (OHI-S), plaque index (PLI), gingival index (GI), gingival bleeding index (GBI), sulcus bleeding index (SBI), bleeding on probing (BOP), probing depth (PD), attachment loss (AL) and so on.

And the additional outcomes comprised:

- Caries and tooth loss: Dental caries and tooth loss can be measured with the following two score indices: ‘decayed, missing and filled teeth’ (DMFT) and ‘decayed, missing and filled surfaces’ (DMFS).
- Oral health-related quality of life (OHRQoL) (dPRO): OHRQoL is measured with self-reported questionnaires, such as Geriatric Oral Health Assessment Index (GOHAI), Oral Health Impact Profile-14 (OHIP-14), Oral Health Impact Profile-5 (OHIP-5) and Oral Health Impact Profile-Edentulism (OHIP-Edent).

Additional data were extracted from included studies, encompassing the following aspects: methods (study design, period, location, setting and funding source), participants (inclusion and exclusion criteria, number of patients, sex, age), interventions and comparators, and other notes.

### Assessment of risk of bias

Risk of bias was assessed independently by two reviewers (Z.Z. and Y.W.), with disagreements resolved via discussion or the involvement of another reviewer (C.L.) as an arbiter [16, 17]. The risk of bias was assessed with the Cochrane Risk of Bias 2 (RoB-2) tool. All risk-of-bias judgments were made based on the effect of assignment to intervention (intention-to-treat effect), which was considered the primary estimand of interest. The five criteria covered by this tool were bias arising from *the randomization process*, *deviations from intended interventions*, *missing outcome data*, *measurement of the outcome*, and *selection of the reported result* [18].

Signaling questions of the RoB-2 were available in Appendix B. We responded to the signaling questions in the RoB-2 tool with response options (yes, probably yes, no, probably no and no information), and the judge each domain as “low risk”, “some concerns”, or “high risk”. We summarized the risk of bias judgements across different studies for each of the domains listed for each outcome. The overall risk of bias was the least-favorable assessment across the domains of bias, including “Low risk”, “Some concerns”, and “High risk”.

### Data synthesis

We performed a random effects model for meta-analysis of comparable data using Review Manager Version 5.4 software (Cochrane Collaboration, Oxford, UK). For continuous outcomes, the standardized mean difference was calculated, whereas relative risk and risk difference was computed for dichotomous data. For all estimates, we computed 95% CIs. We assessed between-study heterogeneity using chi-square tests and I^2^ statistic. Cochrane’s chi-square tests were used to determine the presence of statistical heterogeneity at a significance level of 0.1 [17, 19], and the I^2^ statistic was used to quantify the degree of statistical heterogeneity, with I^2^ values of 25%, 50%, and 75% indicating low, moderate, and high heterogeneity, respectively [17, 19]. All results were accurate to two decimal places.

### Subgroup analysis and sensitivity analysis

We proposed subgroup analysis for different types of interventions, length of follow-up, or characteristics of participants. We used the chi-square test to determine the subgroup differences at a significant level of 0.05. Forest plots were present as the results of data synthesis.

Furthermore, a sensitivity analysis was performed to investigate the robustness of the results wherever appropriate. The level of significance was set as *P* < 0.05. In the event of discrepancies between the sensitivity and main analyses, both sets of results would have been reported and their interpretation considered in the results section.

### Assessment of reporting bias

The assessment of potential reporting biases was methodologically planned contingent upon the inclusion of a sufficient number of studies. Specifically, if a meta-analysis had comprised ten or more trials, a funnel plot would have been generated to visually inspect for potential small-study effects. Furthermore, formal statistical examinations for funnel plot asymmetry, including Egger’s regression test and Begg’s rank correlation test, were pre-specified to investigate the likelihood of publication bias likelihood of publication bias [19].

### Assessment of certainty

Two independent reviewers (Z.Z. and Y.W.) applied the GRADE framework to assess the certainty of the evidence for each comparison, and any discrepancies in their judgments were resolved through consultation with a third reviewer (C.L.) [17].

While evidence derived from randomized controlled trials (RCTs) was initially considered high certainty, it was subject to downgrading (to moderate, low, or very low certainty) based on an evaluation across five key domains: study limitations (risk of bias), indirectness, inconsistency, imprecision of the effect estimates, and publication bias [17, 19].

## Results

### Search results

**Fig. 1:**
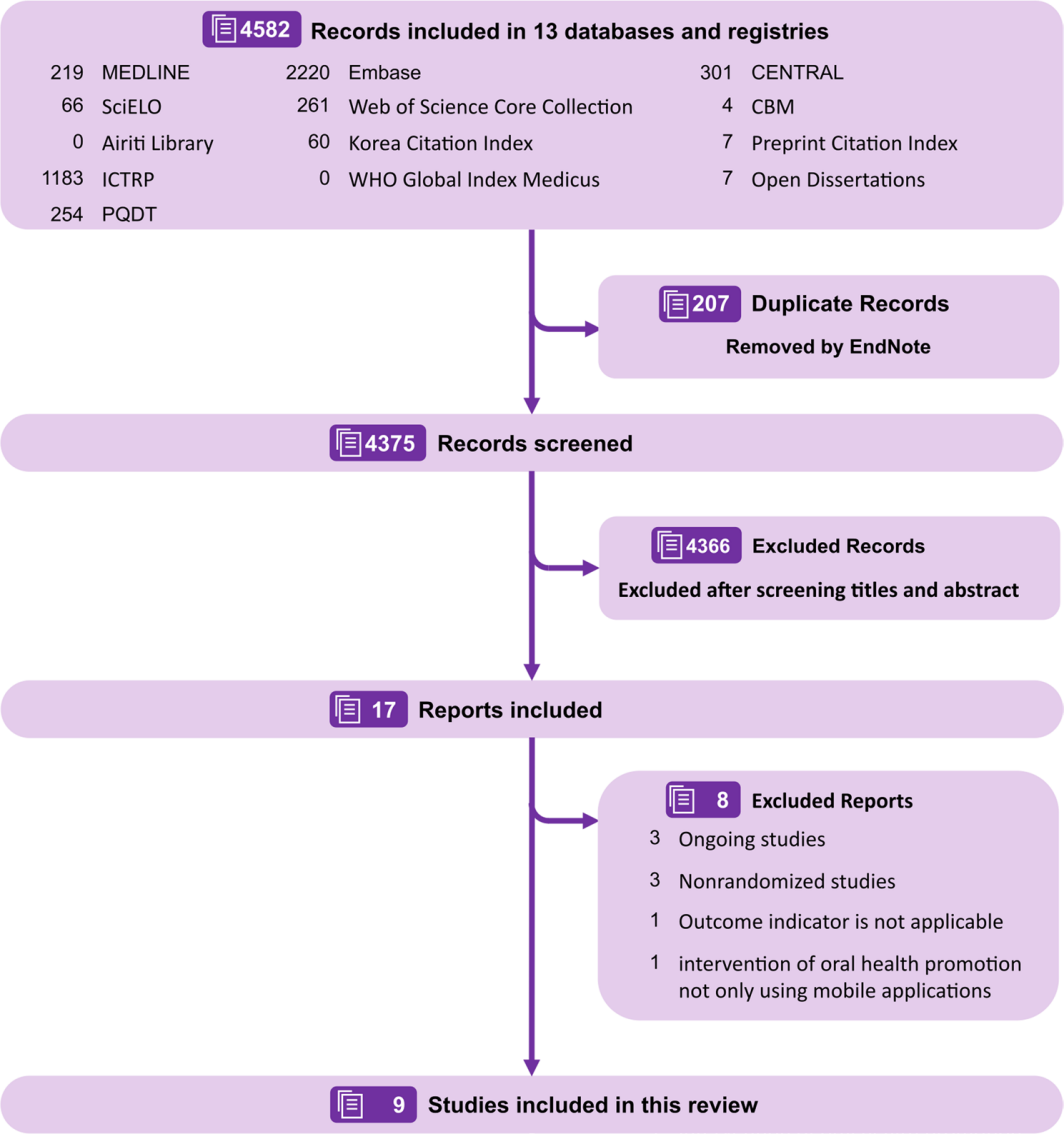
PRISMA 2020 flow diagram for this evidence mapping review: contains the identification, screening, inclusion, and exclusion of studies and reasons for exclusion

As shown in Fig. 1, the initial database searches yielded 4,582 records. Following the removal of 203 duplicates, 4,375 records were subjected to title and abstract screening by two independent reviewers. This process resulted in 42 records being retrieved for a full-text eligibility assessment. Of these, 9 studies met the inclusion criteria and were included in the review, while 32 were excluded primarily due to the unavailability of full text or because they contained only unpublished results. The comprehensive search strategies, along with a list of excluded studies complete with citations and reasons for exclusion, were provided in Appendix A and Appendix C, respectively.

### Characteristics of studies

The selected studies included 402 participants aged over 65 years: 170 allocated to the intervention group and 118 to the conditional control arm, there were also 114 participants allocated to no-treatment control group. Most patients were recruited from senior centers (senior colleges or senior welfare centers) [20–23] and dental practices [24–27], and few studies recruited a sample from society [28].

Regarding the interventions described in the studies, we found that mobile smartphone apps were the most frequently used applications [20–24, 26, 28], and 2 studies utilized text message [25, 27]. The duration of engagement with the strategies was from 2 weeks [22, 23] to 6 months [27], whereas the most common follow-up duration was 2 or 5 weeks [20–23]. Although all interventions were asynchronous, 3 studies provided some instant feedback via text messages, allowing interactions among participants or between participants and researcher [25, 27]. Another study analyzed usage logs and the number of pages viewed per session to reflect usage frequency and intensity of participants [24]. All studies were published in English.

Characteristics of the included studies were summarized in Table 1, with further details available in Appendix D.

**Table 1:** Characteristics of the included studies

Author (Year), Country	Experimental Groups^a^	Follow-up	Main and Secondary Outcomes from Primary Studies
Jade Yi Ming Ng (2021) [24], Malaysia	App use: removable partial denture wearers received education using the PWA Non-app use: removable partial denture wearers received verbal instructions accompanied by demonstration of hygienic procedures.	1 mo. 3 mo. Asynchronous and synchronous	Main: Oral health-related knowledge. PI. GI. DP score
Ji-Yun Ki (2021) [28], Korea	App use: received the OHEMA intervention for 6 weeks Control: did not undergo any oral hygiene education or OHEMA intervention	6 wk. Asynchronous	Main: USFR. Tongue pressure Secondary: Subjective oral dryness.SWAL-QoL
Kyeong Hee Lee (2021) [22], Korea	App use: Oral health education was provided using the smartphone app developed in this study for five weeks Non-app use: received lecture-based oral health education using PowerPoint slides at the affiliated facility twice per week for five weeks Control: did not undergo any oral health education	5 wk. Asynchronous	Main: Oral health-related knowledge. O’Leary index. Winkel tongue coating index. GI Secondary: OHIP-14. GOHAI
Eun Seo Jung (2024) [20], Korea	App use: received combined oral and whole-body exercises for 5 weeks Non-app use: Combined oral and whole- body exercises were performed under the direct guidance of the researchers twice a week for 5 weeks without using the app. Each session lasted for about 1 h. Control: did not undergo any oral health education	5 wk. Asynchronous	Main: USFR. Anterior tongue strength. Posterior tongue strength. Cheek strength Secondary: OHIP-14. GOHAI
Worachate Romalee (2023) [23], Chinese Taiwan	App use: received the OHEMA intervention for 2 weeks Non-app use:received a one-hour lecture on oral health, based on typical programs in Taiwan, was conducted by an experienced oral hygienist Control: did not undergo any oral health education	Immediately. 2 wk. Asynchronous	Main: Oral healthcare-related knowledge. Winkel tongue coating index. PI. GI.
Kristina Wanyonyi (2022) [27], The UK	App use: underwent a ten- week long intervention period where three text messages were sent per week Non-app use: received a leaflet in the post providing the same information contained within the thirty text messages	6 mo. Asynchronous and synchronous	Secondary: OHIP-14
Kyeong Hee Lee (2024) [21], Korea	App use: received the OHEMA intervention 2 times per week, for 6 weeks, 12 times in total Control: no intervention	6 wk. Asynchronous	Main: USFR. O’Leary index. Winkel tongue coating index. Anterior tongue strength. Posterior tongue strength. Cheek strength. GI. Secondary: OHIP-14. GOHAI
Wan Nor Hayati Wan Abd. Manan (2025) [26], Malaysia	App use: received a series of instructional home denture care information videos which were made in-house via WhatsApp and in weeks 1, 3, 5, and 7 after denture insertion and before the postinsertion questionnaires. Non-app use: received instructions at the chairside on denture care in Week 1 and information on the relationship between edentulism and general health in Week 3.	6-8 wk. Asynchronous	Main: Oral healthcare-related knowledge.
Makiko Nishi (2019) [25], Republic of Ireland	App use: received 1 confirmation message, 24 personalized messages weekly, 1 reminder message Non-app use: received 1 confirmation message, 24 non-personalized messages weekly, 1 reminder message	24 wk. Asynchronous and synchronous	Main: Chance of avoiding new cavities.

*Abbreviations*: *PWA* Progressive Web Application, *OHEMA* Oral Health Education Using a Mobile App

^a^App use group referred to intervention using mobile applications, the No-app use group referred to intervention using traditional strategies, and the Control group referred to no intervention being implemented

### Assessment of risk of bias

Given the outcome-specific nature of RoB-2 and the large number of outcomes assessed, risk-of-bias judgments are displayed directly within the forest plots rather than as separate summary figures. Across outcomes, subjectively assessed measures (including OHIP-14, GOHAI, and the Gingival Index) were more frequently judged to be at high risk of bias, whereas objective outcomes were generally rated as having low risk of bias or some concerns. Judgments of some concerns most commonly arose from Domain 1 (randomization process), while high risk of bias was predominantly driven by issues related to Domain 4 (measurement of the outcome) in subjectively assessed measures. A comprehensive documentation of the responses to signaling questions and the rationale for each judgment was provided in Appendix E.

### Assessment of certainty

The certainty of evidence was assessed using the GRADE approach at the outcome level. All studies were graded as either low or very low, primarily due to very serious imprecision and serious to very serious concerns regarding risk of bias. Downgrading for risk of bias was informed by the outcome-specific assessments conducted using the RoB 2 tool. The summarized findings of the GRADE assessments were available in Tables 2 and 3.

**Table 2:** GRADE summary of findings: mobile applications vs. conventional interventions for the promotion of oral health

Outcome	Anticipated Absolute Effects^a^ (95% CI)	Participants (Studies), *n*	GRADE Certainty of Evidence^b^	Comments
	Difference with mobile application			
GI	SMD, 0.37^c^ lower (1.35 lower to 0.60 higher)	60 (2 RCTs)	⊕○○○ Very Low^d,e,f^	Mobile application may result in an invariability in GI.
OHIP-14	SMD, 0.18^c^ lower (0.49 lower to 0.13 higher)	165 (3 RCTs)	⊕○○○ Very Low^d,f^	Mobile application may result in an invariability in OHIP-14.
GOHAI	SMD, 0.04^c^ lower (0.39 lower to 0.47 higher)	97 (2 RCTs)	⊕○○○ Very Low^d,f^	Mobile application may result in an invariability in GOHAI.

*Abbreviations*: *SMD* Standardized Mean Difference

^a^The risk in the intervention group (and its 95% CI) is based on the assumed risk in the comparison group and the relative effect of the intervention (and its 95% CI)

^b^GRADE Working Group grades of evidence: high certainty, we are very confident that the true effect lies close to that of the estimate of the effect; moderate certainty, we are moderately confident in the effect estimate (the true effect is likely to be close to the estimate of the effect, but there is a possibility that it is substantially different); low certainty, our confidence in the effect estimate is limited (the true effect may be substantially different from the estimate of the effect); very low certainty, we have very little confidence in the effect estimate (the true effect is likely to be substantially different from the estimate of effect)

^c^SMD interpreted according to Cohen’s (1988) effect size: 0.2, small; 0.5, moderate; 0.8, large

^d^Serious issues of risk of bias. Studies rated as high risk of bias due to lack or inappropriate concealment of randomization and lack of blinding

^e^Some issues of inconsistency. Unexplained heterogeneity, with point estimates different between studies and confidence intervals narrow or not overlapping (chi-square, *P* = 0.13; I^2^ = 56%)

^f^Serious issues of inaccuracy. Small sample size and 95% CI cross the equivalent line

**Table 3:** GRADE Summary of Findings: Mobile Applications vs. No Interventions for the Promotion of Oral Health

Outcome	Anticipated Absolute Effects^a^ (95% CI)	Participants (Studies), *n*	GRADE Certainty of Evidence^b^	Comments
	Difference with mobile application			
USFR	SMD, 1.93^c^ higher (0.85 to 3.01 higher)	142 (3 RCTs)	⊕○○○ Very Low ^e,f^	Mobile application may result in a large promotion in USFR.
OHIP-14	SMD, 0.82^c^ lower (1.59 to 0.05 lower)	150 (3 RCTs)	⊕○○○ Very Low^d,g^	Mobile application may result in a large reduction in OHIP-14.
GOHAI	SMD, 0.37^c^ lower (0.91 lower to 0.17 higher)	150 (3 RCTs)	⊕○○○ Very Low^d,h^	Mobile application may result in an invariability in GOHAI.
GI	SMD, 1.04^c^ lower (2.72 lower to 0.63 higher)	99 (2 RCTs)	⊕○○○ Very Low^d,f,h^	Mobile application may result in an invariability in GI.
Anterior tongue strength	SMD, 0.00^c^ invariability (0.53 lower to 0.52 higher)	99 (2 RCTs)	⊕⊕○○ Low^h^	Mobile application may result in an invariability in anterior tongue strength.
Posterior tongue strength	SMD, 0.26^c^ higher (0.14 lower to 0.65 higher)	99 (2 RCTs)	⊕⊕○○ Low^h^	Mobile application may result in an invariability in posterior tongue strength.
Cheek strength	SMD, 0.15^c^ lower (0.54 lower to 0.24 higher)	99 (2 RCTs)	⊕⊕○○ Low^h^	Mobile application may result in an invariability in check strength.
O’Leary index	SMD, 0.89^c^ lower (1.79 lower to 0.02 higher)	99 (2 RCTs)	⊕○○○ Very Low^f,h^	Mobile application may result in an invariability in O’Leary index.
Winkel tongue coating index	SMD, 1.02^c^ lower (1.66 lower to 0.39 lower)	99 (2 RCTs)	⊕⊕○○ Low^e,g^	Mobile application may result in a large reduction in Winkel tongue coating index.

*Abbreviations*: *SMD* Standardized Mean Difference

^a^The risk in the intervention group (and its 95% CI) is based on the assumed risk in the comparison group and the relative effect of the intervention (and its 95% CI)

^b^GRADE Working Group grades of evidence: high certainty, we are very confident that the true effect lies close to that of the estimate of the effect; moderate certainty, we are moderately confident in the effect estimate (the true effect is likely to be close to the estimate of the effect, but there is a possibility that it is substantially different); low certainty, our confidence in the effect estimate is limited (the true effect may be substantially different from the estimate of the effect); very low certainty, we have very little confidence in the effect estimate (the true effect is likely to be substantially different from the estimate of effect)

^c^SMD interpreted according to Cohen’s (1988) effect size: 0.2, small; 0.5, moderate; 0.8, large

^d^Serious issues of risk of bias. Studies rated as high risk of bias due to lack or inappropriate concealment of randomization and lack of blinding

^e^Some issues of risk of bias. Studies rated as some concerns risk of bias due to lack or inappropriate concealment of randomization and lack of blinding

^f^Serious issues of inconsistency. Unexplained heterogeneity, with point estimates widely different between studies and confidence intervals not overlapping (I^2^ ≥ 75%)

^g^Some issues of inconsistency. Unexplained heterogeneity, with point estimates difference between studies and confidence intervals narrow or not overlapping (50% ≤ I^2^ ≤ 75%)

^h^Serious issues of inaccuracy. Small sample size and 95% CI cross the equivalent line

### Outcome measures

We were able to conduct meta-analysis for 9 outcomes: gingival index (GI), unstimulated saliva flow rate (USFR), Oral Health Impact Profile-14 (OHIP-14), Geriatric Oral Health Assessment Index (GOHAI), anterior tongue strength, posterior tongue strength, cheek strength, O’Leary index and Winkel tongue coating index.

**Fig. 2:**
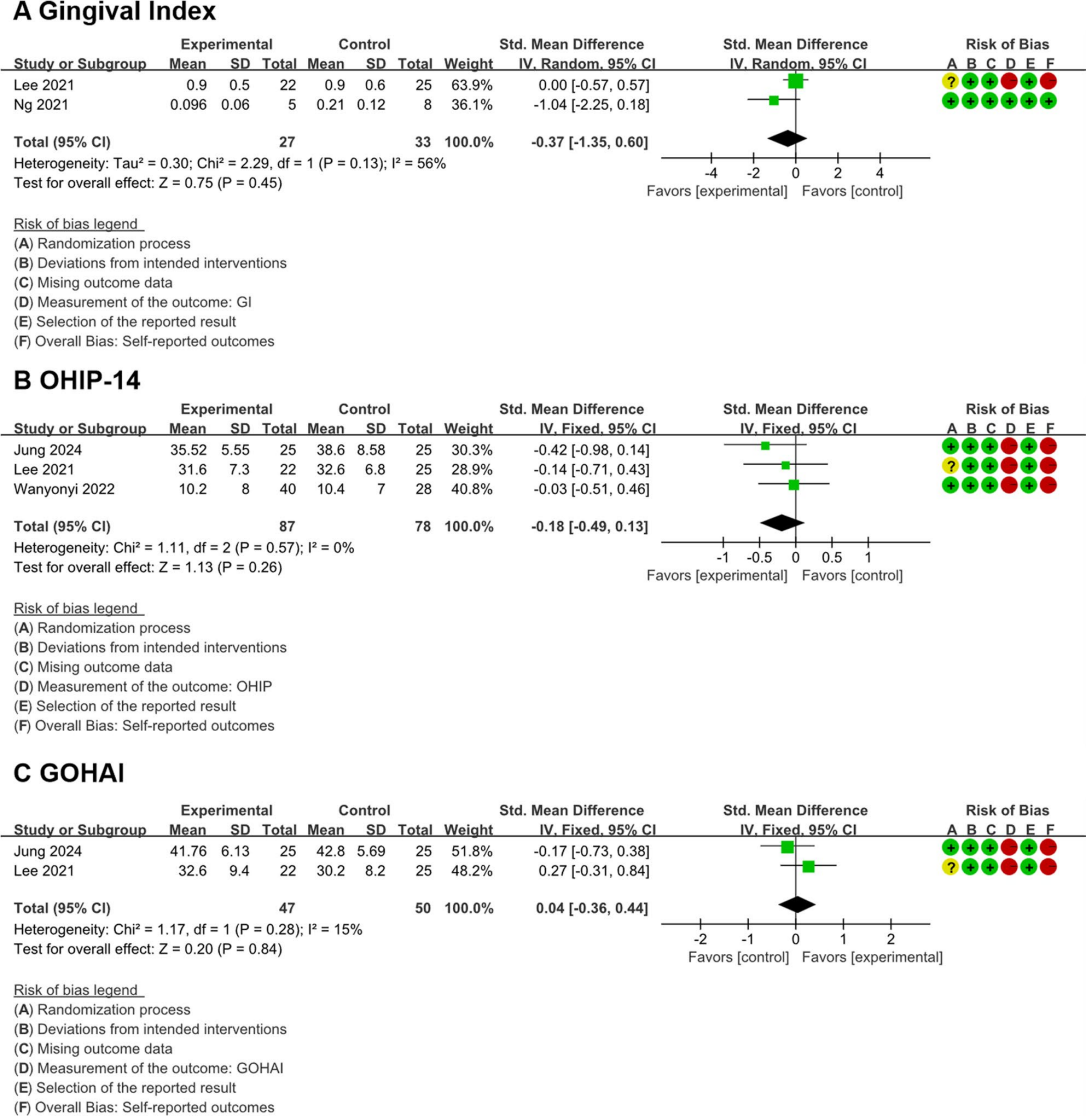
Forest plots: (**A**) gingival index, (**B**) OHIP-14, and (**C**) GOHAI for the longest follow-up value

When compared with conventional education strategies, pooled estimates showed no statistically significant differences associated with mobile application interventions for GI (standardized mean difference, -0.37; 95%CI, -1.35 to 0.60; *P* = 0.45, I^2^ = 56%; very low certainty; Fig. 2A), the OHIP-14 (standardized mean difference, -0.18; 95%CI, -0.49 to 0.13; *P* = 0.26, I^2^ = 0%; very low certainty; Fig. 2B) and the GOHAI (standardized mean difference, 0.04; 95%CI, -0.36 to 0.44; *P* = 0.84, I^2^ = 15%; very low certainty; Fig. 2C).

**Fig. 3:**
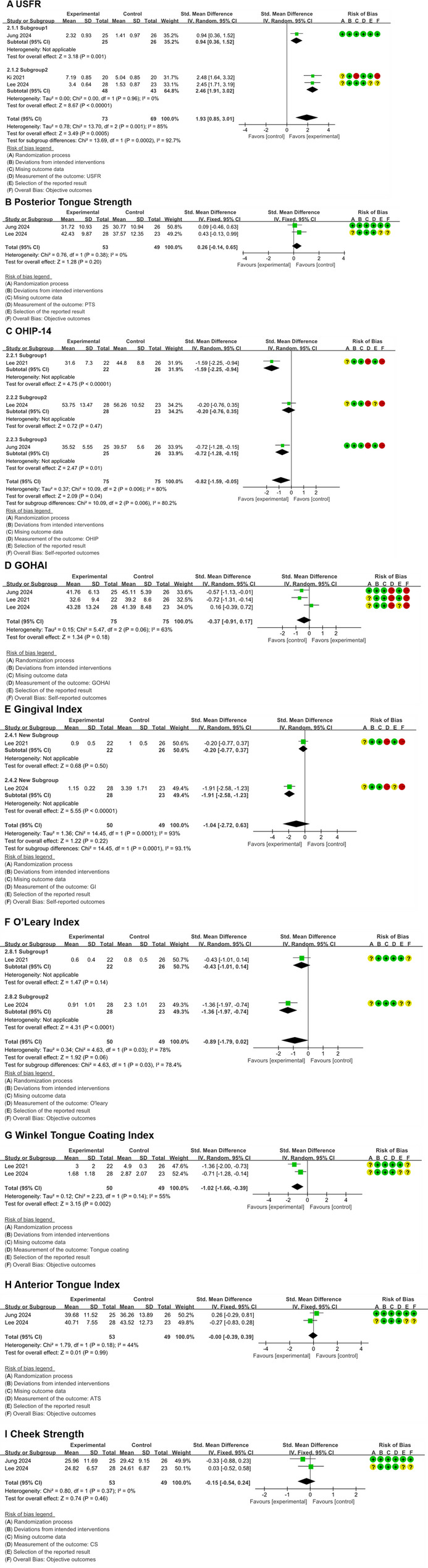
Forest plots: (**A**) USFR, **B** posterior tongue strength and **C** OHIP-14 for the longest follow-up value. Forest plots: (**D**) GOHAI, **E** gingival index and **F** O’Leary index for the longest follow-up value. Forest plots: (**G**) Winkel tongue coating index, **H** anterior tongue strength and **I** cheek strength for the longest follow-up value

But patients as opposed to no intervention experienced promotions of the USFR (standardized mean difference, 1.93; 95%CI, 0.85 to 3.01; *P* = 0.0005, I^2^ = 85%; very low certainty; Fig. 3A), reductions of the OHIP-14 (standardized mean difference, -0.82; 95%CI, -1.59 to -0.05; *P* = 0.04, I^2^ = 80%; very low certainty; Fig. 3C) and the Winkel tongue coating index (standardized mean difference, -1.02; 95%CI, -1.66 to -0.39; *P* = 0.002, I^2^ = 55%; low certainty; Fig. 3G), and invariabilities of the posterior tongue strength (standardized mean difference, 0.24; 95%CI, -0.14 to 0.65; *P* = 0.20, I^2^ = 0%; low certainty; Fig. 3B), the GOHAI (standardized mean difference, -0.37; 95%CI, -0.91 to 0.17; *P* = 0.18, I^2^ = 63%; very low certainty; Fig. 3D), the GI (standardized mean difference, -1.04; 95%CI, -2.72 to 0.63; *P* = 0.22, I^2^ = 93%; very low certainty; Fig. 3E), the O’Leary index (standardized mean difference, -0.89; 95%CI, -1.79 to 0.02; *P* = 0.06, I^2^ = 78%; very low certainty; Fig. 3F), the Anterior tongue strength (standardized mean difference, 0.00; 95%CI, -0.39 to 0.39; *P* = 0.99, I^2^ = 44%; low certainty; Fig. 3H) and the cheek strength (standardized mean difference, -0.15; 95%CI, -0.54 to 0.24; *P* = 0.46, I^2^ = 0%; low certainty; Fig. 3I).

Substantial heterogeneity was observed across several outcomes, and subgroup analyses suggested that differences in intervention type and intervention duration were important contributors to the observed heterogeneity. Given the high heterogeneity and predominantly very low certainty of evidence, the pooled estimates should be interpreted with caution. These meta-analyses are intended to provide a descriptive synthesis of the existing evidence rather than precise estimates of intervention effects.

Several outcomes could not be meta-analyzed because they were reported in single studies or were not comparable across studies (Table 4). For example, Worachate Romalee [23] did not report specific values at endpoint, instead of using changes compared with baseline as a substitute. Thus, it was impossible to group those measures with other indexes, such as GI and oral health-related knowledge. For these outcomes, mobile application interventions generally showed favorable trends compared with no intervention, whereas effects were largely comparable to those of conventional education strategies.

**Table 4:** Outcomes measures from studies not included in meta-analysis

Author (Year)	Main Outcomes^a,b^	Secondary Outcomes
Jade Yi Ming Ng (2021) [24]	Oral health-related knowledge. App use: 16.46 (NR) No-app use: 16.84 (NR); *P* < 0.01 PI. App use: 0.20 (0.13) No-app use: 0.27 (0.17); *P* < 0.01 DP. App use: 2.40 (1.14) No-app use: 2.17 (0.75); *P* < 0.01	NR
Ji-Yun Ki (2021) [28]	Subjective oral dryness. App use: 18.50 (12.61) No-app use: 29.25 (12.61); *P* = 0.42 Tongue pressure. App use: 27.24 (7.83) No-app use: 17.90 (7.38); *P* = 0.99	SWAL-QoL. App use: 171.50 (30.5) No-app use: 163.30 (30.95); *P* = 0.07
Kyeong Hee Lee (2021) [22]	Oral health-related knowledge. App use: 38.10 (6.10) No-app use: 40.00 (4.80) Control: 35.50 (7.80); P = NR	NR
Wan Nor Hayati Wan Abd. Manan (2025) [26]	Oral health-related knowledge. App use: NR No-app use: NR Control: NR; P = NR	NR
Makiko Nishi (2019) [25]	Chance of avoiding new cavities. App use: 44.60 (18.40) No-app use: 35.00 (20.60); *P* = 0.410	NR
Eun Seo Jung (2021) [20]	NR	NR
Kristina Wanyonyi (2022) [27]	NR	NR
Kyeong Hee Lee (2024) [21]	NR	NR
Worachate Romalee (2023) [23]^c^	Oral healthcare-related knowledge: App use: 4.20 (2.95) No-app use: 2.68 (3.12) Control: 0.79 (2.72); *P* < 0.01 Modified plaque score. App use: -14.61 (12.91) No-app use: -13.07 (10.89) Control: -1.88 (12.21); *P* < 0.01 GI. App use: -0.38 (0.29) No-app use: -0.32 (0.31) Control: -0.63 (0.23); *P* < 0.01 Winkel tongue coating index. App use: -3.10 (2.61) No-app use: -2.59 (3.73) Control: 0.47 (3.22); *P* < 0.01	NR

*Abbreviations*: *NR* Not reported, *PI* Plaque index, *DP* Denture plaque, *GI* Gingival index, *SWAL-QoL* Swallowing-quality of life

^a^Results correspond to the last follow-up. Data are presented as mean (SD) or other when stipulated

^b^App use group referred to intervention using mobile applications, the No-app use group referred to intervention using traditional strategies, and the Control group referred to no intervention being implemented

^c^Worachate Romalee, 2023 did not report specific values at endpoint, instead of using changes compared with baseline as a substitute

### Summary of main results

#### GI

The meta-analysis of GI (Figs. 2A and 3E) indicated that there was no statistically significant difference in GI between the mobile applications group, the conventional education strategies group (SMD = -0.37, 95%CI: -1.35 to 0.60), and the no-intervention group (SMD = -1.04, 95%CI: -2.72 to 0.62). Based on the GRADE criteria, the certainty of this evidence was rated as very low. However, significant heterogeneity was demonstrated in this meta-analysis (I^2^ = 93%), with the SMD being markedly higher in Subgroup 1 than in Subgroup 2. This may be attributable to the studies in Subgroup 2 employing more intervention times, thereby yielding a more pronounced reduction within the intervention group compared to the non-intervention group. These results indicate that the effectiveness of mobile applications and conventional education strategies is not significant. However, the overall reliability of the evidence is limited.

#### USFR

As shown in Fig. 3A, the USFR of the mobile applications group was higher compared with that of the no-intervention group (SMD = 1.93, 95%CI: 0.85 to 3.01). Nevertheless, the certainty of this evidence was also rated as very low according to the GRADE criteria. However, significant heterogeneity was demonstrated in this meta-analysis (I^2^ = 85%), with the SMD being markedly lower in Subgroup 1 than in Subgroup 2. This may be attributable to the fact that studies in Subgroup 2 employed OHEMA interventions over a six-week period, whereas studies in Subgroup 1 utilized different interventions administered over a five-week duration.

#### OHIP-14

The meta-analysis of OHIP-14 (Figs. 2B and 3C) revealed that participants using mobile applications reported lower scores compared with those receiving no intervention (SMD = -0.82, 95%CI: -1.59 to -0.05), indicating better oral health-related quality of life. However, no statistically significant differences were found between the mobile application group and the conventional education group (SMD = -0.18, 95%CI: -0.49 to 0.13). However, significant heterogeneity was demonstrated in this meta-analysis (I^2^ = 80%), with significant differences in SMD between subgroups. The reasons may be that there were certain differences in both the intervention measures and the duration of intervention across subgroups. The certainty of this evidence was graded as very low.

#### GOHAI

The meta-analysis of GOHAI (Figs. 2C and 3D) showed that there was no statistically significant difference in scores between the mobile applications group, the conventional education strategies group (SMD = -0.04, 95%CI: -0.36 to 0.44), and the no-intervention group (SMD = -0.37, 95%CI: -0.91 to 0.17). The GRADE assessment rated the quality of evidence as very low.

#### Anterior tongue strength

The meta-analysis of anterior tongue strength (Fig. 3H) revealed no significant difference between the mobile application group and the no-intervention group (SMD = 0.00, 95%CI: -0.39 to 0.39). According to the GRADE assessment, the quality of the evidence was rated as low.

#### Posterior tongue strength

The meta-analysis of posterior tongue strength (Fig. 3B) showed no statistically significant difference between the mobile application group and the no-intervention group (SMD = 0.26, 95%CI: -0.14 to 0.65). The GRADE assessment rated the quality of evidence as low.

#### Cheek strength

The meta-analysis of cheek strength (Fig. 3I) demonstrated no statistically significant difference between the mobile application group and the no-intervention group (SMD = -0.15, 95%CI: -0.54 to 0.24). The GRADE assessment rated the evidence quality as low.

#### O’Leary index

The meta-analysis of the O’Leary Index (Fig. 3F) suggested a potential difference between the mobile application group and the no-intervention group (SMD = -0.89, 95%CI: -1.79 to 0.02), although this did not reach statistical significance. The GRADE assessment classified the evidence quality as very low. However, significant heterogeneity was demonstrated in this meta-analysis (I^2^ = 78%), with the SMD being markedly higher in Subgroup 1 than in Subgroup 2. This may be attributable to the studies in Subgroup 2 employing more intervention times, thereby yielding a more pronounced reduction in the O’Leary index within the intervention group compared to the non-intervention group.

#### Winkel tongue coating index

The meta-analysis of the Winkel Tongue Coating Index (Fig. 3G) found a statistically significant difference favoring the mobile application group over the no-intervention group (SMD = -1.02, 95%CI: -1.66 to -0.39). However, the GRADE assessment rated the certainty of the evidence as low, limiting the reliability of this finding.

## Discussion

Previous reviews [19, 29, 30] have summarized the impact of mobile applications on oral health; however, most of the included populations were adolescents and young adults, leaving a critical gap regarding the elderly. In this review, a total of 402 elderly individuals aged over 65 years with preserved cognitive and self-care abilities were included. By specifically targeting this population, our study provides evidence that can help clinicians and policymakers more comprehensively understand the role of mobile applications in oral health promotion at the population level, across the lifespan. The included studies had intervention durations ranging from two weeks to six months, with five weeks being the most common.

This study found evidence supporting the use of mobile applications to promote oral health in the elderly. Our meta-analysis demonstrated that, compared with no intervention, the use of mobile applications led to statistically and clinically significant improvements in several outcomes, including increased USFR, reduced OHIP-14 scores, and reduced Winkel tongue coating index. However, no significant differences were observed in other indicators such as the GOHAI, GI, anterior tongue strength, posterior tongue strength, cheek strength, and the O’Leary index. Furthermore, when compared to conventional interventions, mobile applications did not demonstrate either superiority or inferiority, indicating that both approaches may have a positive impact on oral health outcomes. Regarding the sustainability of the effects, the findings suggest that the benefits of mobile applications may be maintained over time, with some evidence indicating that effects could even strengthen with prolonged use. 2 studies included intervention periods of up to three months, implying that consistent use may lead to more substantial improvements.

However, substantial heterogeneity was observed across several outcome measures, including USFR, OHIP-14, the Gingival Index, and the O’Leary Index. This heterogeneity is likely attributable to pronounced clinical and methodological differences among the included studies. Although all interventions were broadly classified as app-based oral health interventions, they varied considerably in content, intensity, and underlying mechanisms of action. Some interventions primarily focused on oral health education [22–24, 26], whereas others incorporated structured oral or whole-body exercise programs [20, 21], and still others relied on personalized reminder systems delivered through tailored messaging [25, 27]. In addition, intervention duration ranged widely from two to 24 weeks, which may have further contributed to heterogeneity in intervention effects, particularly for outcomes sensitive to intervention dose and duration, such as salivary flow rate and oral health–related quality of life. Subgroup analyses suggested that differences in intervention type and intervention duration were important contributors to the observed heterogeneity.

In addition to heterogeneity in the intervention arms, the conventional education strategies used as comparators were also highly heterogeneous and lacked standardization. These control interventions ranged from verbal or chairside education [24, 26] and structured educational lectures [22, 23] to written educational materials, such as pamphlets or leaflets [27]. Substantial variation existed in content depth, frequency of delivery, and degree of participant engagement. Importantly, some comparator interventions may themselves have exerted meaningful behavioral effects, whereas others more closely resembled low-intensity or passive information provision. Such inconsistency in control intervention intensity not only increases clinical heterogeneity across studies but also complicates the interpretation of relative effect estimates when comparing app-based interventions with “conventional education.” Consequently, the pooled comparative results should be interpreted with caution, as they may reflect differences in control conditions as much as differences in intervention effectiveness. Consideration of the specific clinical or public health contexts represented by these control strategies is therefore essential when assessing the applicability and generalizability of the findings.

Compared with earlier reviews in this field [19, 29], our study is distinguished by its focus on individuals aged over 65 years and by the inclusion of comparisons against no-intervention controls. Unlike previous reviews that reported superior effects of mobile applications over conventional strategies, our findings showed benefits only when compared with no intervention, but not when compared with conventional education strategies. A plausible explanation for this discrepancy lies in differences in study populations. Adolescents and young adults are generally more familiar with digital environments and smartphone use, which may facilitate engagement with mobile-based oral health interventions [31]. In contrast, older adults—particularly those with limited prior exposure to smartphones—may experience greater barriers to engagement, potentially attenuating the relative effectiveness of app-based interventions when compared with conventional approaches.

Against the backdrop of the digital divide, older adults generally demonstrate lower familiarity with and acceptance of information technologies compared with younger populations [32]. Consequently, the effectiveness of app-based interventions among older adults is highly dependent on user interface and user experience (UI/UX) design [33]. Age-related changes, including visual impairment (e.g., presbyopia), cognitive decline, and reduced fine motor skills, necessitate targeted adaptations in app design, such as larger font sizes, simplified navigation, and reduced interaction complexity [34]. Among the included studies, some trials developed dedicated mobile applications and explicitly reported user-friendly interfaces that allowed tasks to be completed through simple touch-based interactions [20, 22, 24]. In contrast, other studies did not specify whether age-related needs were considered during the app development process [21, 28]. In addition, several interventions relied on existing platforms such as WhatsApp [26] or MAKAR [23], which were not originally designed for older users. Although operational training was provided before intervention initiation in all studies, limited consideration of age-related functional constraints at the design stage may still have resulted in usability barriers. Such differences in usability may have influenced participant engagement and adherence and, consequently, contributed to the heterogeneity observed in intervention effects.

In addition, digital health interventions inherently rely on participants’ cognitive and functional capacities to engage with app-based technologies. Accordingly, most included studies restricted eligibility to older adults with adequate cognitive function and self-care ability. However, frailty and mild cognitive impairment are common in the general older population [35], which may limit individuals’ ability to effectively use and adhere to such digital interventions. These eligibility criteria may have introduced selection bias, whereby relatively healthier older adults with higher technological competence were more likely to be enrolled. As a result, the generalizability of the findings to the broader older population—particularly those with functional or cognitive vulnerability—may be limited.

Despite these challenges, mobile applications may offer several advantages over conventional interventions, making them a potentially viable tool for elderly populations. One of the primary advantages is cost-effectiveness. Mobile applications can provide personalized and tailored interventions at a lower cost compared to traditional methods, which can be particularly beneficial for older adults who may have limited access to healthcare resources [36]. Additionally, mobile applications offer ease of access and convenience, enabling older adults to participate in health-promoting activities from their own homes. Evidence suggests that mobile apps can improve oral health knowledge among older adults, enhance the accessibility of knowledge acquisition [26], and are associated with high user satisfaction in this demographic [37].

Furthermore, personalized interventions delivered via mobile apps may improve user compliance and motivation, support sustained behavioral change, and encourage the adoption of health-positive habits [25]. Where feasible, clinicians should consider developing tailored oral health education plans based on patients’ baseline characteristics and individual needs. Future research should therefore include more comprehensive evaluations and explore the integration of personalized strategies—such as motivational interviewing—delivered by dental professionals. Such approaches may strengthen the existing evidence base and improve the practical implementation of mobile applications in promoting oral health among the elderly.

The strengths of this review lie in its rigorous methodology: study selection, data extraction, and risk of bias assessments were conducted independently and repeatedly by two researchers, with verification by a third, thereby enhancing methodological rigor and quality control. Additionally, the review adopted GRADE to assess certainty of evidence, and clarified the implications of relevant variables for clinical and policy decision-making.

Nevertheless, several limitations should be acknowledged. First, the certainty of evidence for all outcomes was rated as very low according to the GRADE framework, indicating limited confidence in the estimated effects. Second, substantial heterogeneity was observed for several outcomes, including the gingival index, USFR, OHIP-14, and the O’Leary index. Accordingly, the pooled effect estimates should be interpreted with caution and are intended to provide a descriptive synthesis of the available evidence rather than precise estimates of intervention effects. Third, as most included studies were conducted in East Asia [20–22, 24–26, 28], the generalizability of the findings to elderly populations in other regions remains uncertain. Finally, the small number of included studies and the limited total sample size substantially reduce statistical power, increasing the risk of type II error. As a result, the absence of statistically significant effects for several outcomes should be interpreted with caution, as true effects of small to moderate magnitude cannot be excluded.

## Conclusions

This review suggested that mobile applications may have effects on oral health promotion among elderly individuals aged over 65 years compared to no interventions, including increasing USFR and lowering OHIP-14 and Winkel tongue coating index. However, relative to conventional strategies, there had been no significant change in the performance of the mobile applications. It was suggested that mobile applications may be as effective as traditional strategies. But the findings of this review should be interpreted with caution, as the precision of the results is insufficient and the overall reliability of the evidence was limited.

Future research should focus on large-scale, rigorously designed studies to provide more robust evidence regarding the impact of mobile applications on the oral health of elderly individuals. Clinicians and policymakers are advised to interpret the present findings carefully and to tailor interventions to the individual needs and characteristics of older patients.

## Supplementary Information

Supplementary Material 1.

## Data Availability

All data generated or analyzed during this study are included in this published article and its supplementary information files.

## Abbreviations

RCTs: Randomized Controlled Trials
SMD: Standardized mean differences
GRADE: Grading of Recommendations Assessment, Development and Evaluation
PRISMA: Preferred Reporting Items for Systematic Reviews and Meta-analysis
OHI-S: Simplified Oral Hygiene Index
PLI: Plaque Index
GI: Gingival Index
GBI: Gingival Bleeding Index
SBI: Sulcus Bleeding Index
BOP: Bleeding On Probing
PD: Probing Depth
AL: Attachment Loss
DMFT: Decayed, Missing and Filled Teeth
DMFS: Decayed, Missing and Filled Surfaces’
OHRQoL: Oral Health-Related Quality of Life
GOHAI: Geriatric Oral Health Assessment Index
OHIP-14: Oral Health Impact Profile-14
OHIP-5: Oral Health Impact Profile-5
OHIP-Edent: Oral Health Impact Profile-Edentulism
RoB-2: Cochrane Risk of Bias 2 tool
