# Supplementary material for: Effect of mobile applications in oral health promotion among elderly people: a systematic review and meta-analysis
Source: BMC Oral Health. 2026 Apr 6;26:896. doi: 10.1186/s12903-026-08202-z (PMC13202910; doi:10.1186/s12903-026-08202-z)
Supplement: Supplementary file 1 — Supplementary Material 1. [file 12903_2026_8202_MOESM1_ESM.docx]

# Appendix A: Search Strategies

Ovid MEDLINE(R) and Epub Ahead of Print, In-Process, In-Data-Review & Other Non-Indexed Citations, Daily and Versions <1946 to January 28, 2025>

| Number | Searches | Results |
| --- | --- | --- |
| 1 | exp Aged/ or exp Geriatrics/ or exp Geriatric Nursing/ | 3655992 |
| 2 | (centarian* or centenarian* or elder* or eldest or frail* or geriatric* or nonagenarian* or octogenarian* or old age* or older adult or older age* or older female* or older male* or older man or older men or older patient* or older people or older person* or older population or older subject* or older woman or older women or oldest old* or senior* or senium or septuagenarian* or supercentenarian* or very old*).mp. | 713083 |
| 3 | (exp Adolescent/ or exp Child/) not Adult/ | 1820177 |
| 4 | (animals not humans).sh. | 5267077 |
| 5 | (1 or 2) not (3 or 4) | 3863785 |
| 6 | exp Dentistry/ or Oral Medicine/ or exp Tooth Diseases/ or exp Mouth Diseases/ or exp Oral Hygiene/ or Oral Health/ or Health Education, Dental/ or exp Tooth/ | 808952 |
| 7 | (caries or carious or decay* or white spot* or dmf or dmft or dmfs).mp. | 199757 |
| 8 | (periodonti* or periodontal or gingiviti* or gingiva*).mp. | 158375 |
| 9 | (periodontal index* or periodontal indices or periodontal disease index* or periodontal disease indices or CPI or CPITN or gingival index* or gingival indices or bleeding index* or bleeding indices or bleeding on probing or BOP or probing depth* or pocket depth* or attachment level* or attachment loss or oral hygiene index* or oral hygiene indices or plaque index* or plaque indices).mp. | 34448 |
| 10 | (tooth loss* or missing teeth or partial edentulism or patrial tooth loss* or dental arch defect* or dentition defect* or tooth absence or edentulous space* or dental gap* or tooth agenesis or alveolar bone loss*).mp. | 26655 |
| 11 | (dental prosthesis* or denture or fixed partial denture or complete denture or partial denture or removable patrial denture or overdenture).mp. | 68084 |
| 12 | (dental implant* or implant-retained denture or implant placement*).mp. | 55763 |
| 13 | ((oral or mouth* or dental or tooth* or teeth) adj3 (hygiene or health* or care or prevent* or prophyla*)).mp. | 137476 |
| 14 | exp Dental Materials/ or exp Dentifrices/ or exp Mouthwashes/ | 153241 |
| 15 | (mouthwash* or mouthrins* or toothbrush* or toothpaste* or dentifrice*).mp. | 25594 |
| 16 | ((oral or mouth* or dental or tooth* or teeth) adj5 (wash* or rins* or bath* or clean* or brush* or polish* or paste* or foam* or gel*)).mp. | 22504 |
| 17 | (plaque adj3 (control* or remov*)).mp. | 5248 |
| 18 | or/6-17 | 1073172 |
| 19 | 5 and 18 | 109577 |
| 20 | Geriatric Dentistry/ | 1019 |
| 21 | 19 or 20 | 109584 |
| 22 | exp Telemedicine/ or Digital Health/ or Dental Informatics/ or Internet/ or Internet Access/ or Internet-Based Intervention/ or "Internet Use"/ or Wireless Technology/ or Telephone/ or Cell Phone/ or "Cell Phone Use"/ or Smartphone/ or Text Messaging/ or Computers, Handheld/ or exp Wearable Electronic Devices/ or Mobile Applications/ or Blogging/ or Social Media/ or exp Social Networking/ | 221658 |
| 23 | (tele health* or telehealth* or electronic health* or ehealth* or moblie health* or mhealth* or digital health* or tele care or telecare).mp. | 100281 |
| 24 | (mobile* or phone* or telephone* or cellphone* or smartphone* or iphone or ipad or android or pda or app or apps or smart watch* or smartwatch* or smart glasses or smartglasses or smart bracelet* or smart band* or smartband*).mp. | 358381 |
| 25 | ((mobile* or smart* or portable or pocket* or handheld or hand held or palm* or tablet) adj3 (deviceor computer* or microcomputer* or pc)).mp. | 30645 |
| 26 | ((mobile* or smart* or portable or pocket* or handheld or hand held or palm* or wireless or electronic or digital or online) adj3 (assistant* or tablet*)).mp. | 4087 |
| 27 | ((mobile* or smart* or portable or pocket* or handheld or hand held or palm* or wireless or electronic or digital or online or web or website or site or internet or tablet) adj15 (applicationor program* or software or platform or algorithm* or messag* or track* or reminder* or video*)).mp. | 232842 |
| 28 | (social adj3 (media or network* or web or website or site or platform)).mp. | 76585 |
| 29 | (baidu or bilibili or blog* or facebook or flickr or gagahi or google* or gtalk or hashtag or hellotalk or icq or instagram or kakaotalk or kik or kuaishou or linkedin or metaverse or msn or myspace or oicq or paltalk or pinterest or qq or quora or reddit or sina or skout or skype or snapchat or sohu or sweetalk or telegram or tencent or tiktok or tinder or triller or tumblr or twitter or viber or vine or wechat or weibo or weixin or whatsapp or worldtalk or yahoo or youku or youtube or zhihu or zorpia).mp. | 101062 |
| 30 | or/22-29 | 856440 |
| 31 | 21 and 30 | 2314 |
| 32 | exp randomized controlled trial/ | 632992 |
| 33 | controlled clinical trial.pt. | 95682 |
| 34 | randomized.ab. | 678358 |
| 35 | placebo.ab. | 255726 |
| 36 | clinical trials as topic.sh. | 204271 |
| 37 | randomly.ab. | 452081 |
| 38 | trial.ti. | 328185 |
| 39 | or/32-38 | 11628360 |
| 40 | exp animals/ not humans.sh. | 5301552 |
| 41 | 39 not 40 | 132529 |
| 42 | 31 and 41 | 219 |

## Database: EBM Reviews - Cochrane Central Register of Controlled Trials < **January 2025**>

| Number | Searches | Results |
| --- | --- | --- |
| 1 | exp Aged/ or Geriatrics/ or exp Geriatric Nursing/ | 283180 |
| 2 | (centarian* or centenarian* or elder* or eldest or frail* or geriatri* or nonagenarian* or octagenarian* or octogenarian* or old age* or older adult* or older age* or older female* or older male* or older man or older men or older patient* or older people or older person* or older population or older subject* or older woman or older women or oldest old* or senior* or senium or septuagenarian* or supercentenarian* or very old*).mp. | 109265 |
| 3 | (exp Adolescent/ or exp Child/) not Adult/ | 73838 |
| 4 | (animals not humans).sh. | 3653 |
| 5 | (1 or 2) not (3 or 4) | 354647 |
| 6 | exp Dentistry/ or Oral Medicine/ or exp Tooth Diseases/ or exp Mouth Diseases/ or exp Oral Hygiene/ or Oral Health/ or Health Education, Dental/ or exp Tooth/ | 40742 |
| 7 | (caries or carious or decay* or white spot* or dmf or dmft or dmfs).mp. | 13234 |
| 8 | (periodonti* or periodontal or gingiviti* or gingiva*).mp. | 23313 |
| 9 | (periodontal index* or periodontal indices or periodontal disease index* or periodontal disease indices or CPI or CPITN or gingival index* or gingival indices or bleeding index* or bleeding indices or bleeding on probing or BOP or probing depth* or pocket depth* or attachment level* or attachment loss or oral hygiene index* or oral hygiene indices or plaque index* or plaque indices).mp. | 13045 |
| 10 | (tooth loss* or missing teeth or partial edentulism or patrial tooth loss* or dental arch defect* or dentition defect* or tooth absence or edentulous space* or dental gap* or tooth agenesis or alveolar bone loss*). mp. | 3110 |
| 11 | (dental prosthesis* or denture or fixed partial denture or complete denture or partial denture or removable patrial denture or overdenture).mp. | 3993 |
| 12 | (dental implant* or implant-retained denture or implant placement*).mp. | 6001 |
| 13 | ((oral or mouth* or dental or tooth* or teeth) adj3 (hygiene or health* or care or prevent* or prophyla*)).mp. | 22153 |
| 14 | exp Dental Materials/ or exp Dentifrices/ or exp Mouthwashes/ | 13484 |
| 15 | (mouthwash* or mouthrins* or toothbrush* or toothpaste* or dentifrice*).mp. | 11498 |
| 16 | ((oral or mouth* or dental or tooth* or teeth) adj5 (wash* or rins* or bath* or clean* or brush* or polish* or paste* or foam* or gel*)).mp. | 9929 |
| 17 | (plaque adj3 (control* or remov*)).mp. | 2979 |
| 18 | or/6-17 | 78025 |
| 19 | 5 and 18 | 9247 |
| 20 | Geriatric Dentistry/ | 1 |
| 21 | 19 or 20 | 9247 |
| 22 | exp Telemedicine/ or Digital Health/ or Dental Informatics/ or Internet/ or Internet Access/ or Internet-Based Intervention/ or "Internet Use"/ or Wireless Technology/ or Telephone/ or Cell Phone/ or "Cell Phone Use"/ or Smartphone/ or Text Messaging/ or Computers, Handheld/ or exp Wearable Electronic Devices/ or Mobile Applications/ or Blogging/ or Social Media/ or exp Social Networking/ | 18947 |
| 23 | (tele health* or telehealth* or electronic health* or ehealth* or moblie health* or mhealth* or digital health* or tele care or telecare).mp. | 13043 |
| 24 | (mobile* or phone* or telephone* or cellphone* or smartphone* or iphone or ipad or android or pda or app or apps or smart watch* or smartwatch* or smart glasses or smartglasses or smart bracelet* or smart band* or smartband*).mp. | 68934 |
| 25 | ((mobile or smartor portable or pocket* or handheld or hand held or palm* or tablet) adj3 (deviceor computer* or microcomputer* or pc)).mp. | 6050 |
| 26 | ((mobile* or smart* or portable or pocket* or handheld or hand held or palm* or wireless or electronic or digital or online) adj3 (assistant* or tablet*)).mp. | 1542 |
| 27 | ((mobile* or smart* or portable or pocket* or handheld or hand held or palm* or wireless or electronic or digital or online or web or website or site or internet or tablet) adj15 (applicationor program* or software or platform or algorithm* or messag* or track* or reminder* or video*)).mp. | 41186 |
| 28 | (social adj3 (media or network* or web or website or site or platform)).mp. | 6632 |
| 29 | (baidu or bilibili or blog* or facebook or flickr or gagahi or google* or gtalk or hashtag or hellotalk or icq or instagram or kakaotalk or k ik or kuaishou or linkedin or metaverse or msn or myspace or oicq or paltalk or pinterest or qq or quora or reddit or sina or skout or skype or snapchat or sohu or sweetalk or telegram or tencent or tiktok or tinder or triller or tumblr or twitter or viber or vine or wechat or weibo or weixin or whatsapp or worldtalk or yahoo or youku or youtube or zhihu or zorpia).mp. | 5962 |
| 30 | or/22-29 | 111448 |
| 31 | 21 and 30 | 301 |

## Embase <1974 to 2025 January 14>

| Number | Searches | Results |
| --- | --- | --- |
| 1 | (centarian* or centenarian* or elder* or eldest or frail* or geriatric* or nonagenarian* or octogenarian* or octogenarian* or old age* or older adult or older age* or older female* or older male* or older man or older men or older age* or older female* or older male* or older man or older men or older patient* or older people or older person* or older population or older subject* or older woman or older women or oldest old* or senior* or senium or septuagenarian* or supercentenarian* or very old*).mp. [mp=title, abstract, heading word, drug trade name, original title, device manufacturer, drug manufacturer, device trade name, keyword heading word, floating subheading word, candidate term word] | 1326067 |
| 2 | exp Aged/ or exp Geriatrics/ or exp Geriatric Nursing/ | 4338865 |
| 3 | ( exp Adolescent/ or exp Child/) not Adult/ | 2794327 |
| 4 | (animals not humans).sh. | 0 |
| 5 | (1 or 2) not (3 or 4) | 4666952 |
| 6 | exp Dentistry/ or Oral Medicine/ or exp Tooth Diseases/ or exp Mouth Diseases/ or exp Oral Hygiene/ or Oral Health/ or Health Education, Dental/ or exp Tooth/ | 1134223 |
| 7 | (caries or carious or decay* or white spot* or dmf or dmft or dmfs).mp. | 219344 |
| 8 | (periodonti* or periodontal or gingiviti* or gingiva*).mp. | 193129 |
| 9 | (periodontal index* or periodontal indices or periodontal disease index* or periodontal disease indices or CPI or CPITN or gingival index* or gingival indices or bleeding index* or bleeding indices or bleeding on probing or BOP or probing depth* or pocket depth* or attachment level* or attachment loss or oral hygiene index* or oral hygiene indices or plaque index* or plaque indices).mp. | 38319 |
| 10 | (tooth loss* or missing teeth or partial edentulism or patrial tooth loss* or dental arch defect* or dentition defect* or tooth absence or edentulous space* or dental gap* or tooth agenesis or alveolar bone loss*).mp. | 22798 |
| 11 | (dental prosthesis* or denture or fixed partial denture or complete denture or partial denture or removable patrial denture or overdenture).mp. | 56905 |
| 12 | (dental implant* or implant-retained denture or implant placement*).mp. | 36454 |
| 13 | ((oral or mouth* or dental or tooth* or teeth) adj3 (hygiene or health* or care or prevent* or prophyla*)).mp. | 133831 |
| 14 | exp Dental Materials/ or exp Dentifrices/ or exp Mouthwashes/ | 156273 |
| 15 | (mouthwash* or mouthrins* or toothbrush* or toothpaste* or dentifrice*).mp. | 27904 |
| 16 | ((oral or mouth* or dental or tooth* or teeth) adj5 (wash* or rins* or bath* or clean* or brush* or polish* or paste* or foam* or gel*)).mp. | 35102 |
| 17 | (plaque adj3 (control* or remov*)).mp. | 6361 |
| 18 | or/6-17 | 1492648 |
| 19 | 5 and 18 | 177914 |
| 20 | Geriatric Dentistry/ | 64 |
| 21 | 19 or 20 | 177915 |
| 22 | exp Telemedicine/ or Digital Health/ or Dental Informatics/ or Internet/ or Internet Access/ or Internet-Based Intervention/ or "Internet Use"/ or Wireless Technology/ or Telephone/ or Cell Phone/ or "Cell Phone Use"/ or Smartphone/ or Text Messaging/ or Computers, Handheld/ or exp Wearable Electronic Devices/ or Mobile Applications/ or Blogging/ or Social Media/ or exp Social Networking/ | 459786 |
| 23 | (tele health* or telehealth* or electronic health* or ehealth* or moblie health* or mhealth* or digital health* or tele care or telecare).mp. | 152278 |
| 24 | (mobile* or phone* or telephone* or cellphone* or smartphone* or iphone or ipad or android or pda or app or apps or smart watch* or smartwatch* or smart glasses or smartglasses or smart bracelet* or smart band* or smartband*).mp. | 563463 |
| 25 | ((mobile* or smart* or portable or pocket* or handheld or hand held or palm* or tablet) adj3 (deviceor computer* or microcomputer* or pc)).mp. | 46518 |
| 26 | ((mobile* or smart* or portable or pocket* or handheld or hand held or palm* or wireless or electronic or digital or online) adj3 (assistant* or tablet*)).mp. | 8255 |
| 27 | ((mobile* or smart* or portable or pocket* or handheld or hand held or palm* or wireless or electronic or digital or online or web or website or site or internet or tablet) adj15 (applicationor program* or software or platform or algorithm* or messag* or track* or reminder* or video*)).mp. | 363517 |
| 28 | (social adj3 (media or network* or web or website or site or platform)).mp. | 126499 |
| 29 | (baidu or bilibili or blog* or facebook or flickr or gagahi or google* or gtalk or hasht ag or hellotalk or icq or instagram or kakaotalk or kik or kuaishou or linkedin or metaverse or ms n or myspace or oicq or paltalk or pinterest or qq or quora or reddit or sina or skout or skype or snapchat or sohu or sweetalk or telegram or tencent or tiktok or tinder or triller or tumblr or twitter or viber or vine or wechat or weibo or weixin or whatsapp or worldtalk or yahoo or youku or youtube or zhihu or zorpia).mp. | 137933 |
| 30 | or/22-29 | 1303563 |
| 31 | 21 and 30 | 5618 |
| 32 | exp randomized controlled trial/ | 1097203 |
| 33 | controlled clinical trial/ | 460005 |
| 34 | random$.ti,ab. | 2476703 |
| 35 | randomization/ | 101242 |
| 36 | intermethod comparison/ | 315891 |
| 37 | placebo.ti,ab. | 461923 |
| 38 | (compare or compared or comparison).ti,ab. | 8752796 |
| 39 | ((evaluated or evaluate or evaluating or assessed or assess) and (compare or compared or comparing or comparison)).ab. | 3312018 |
| 40 | (open adj label).ti,ab. | 186451 |
| 41 | ((double or single or doubly or singly) adj (blind or blinded or blindly)).ti,ab. | 365873 |
| 42 | double blind procedure/ | 307174 |
| 43 | parallel group$1.ti,ab. | 52552 |
| 44 | (crossover or cross over).ti,ab. | 155671 |
| 45 | ((assign*ormatchormatchedorallocation*)*adj*5(*alternateorgroup*1 or intervention1*orpatient*1 or subject1*orparticipant*1)). ti,ab. | 508047 |
| 46 | (assigned or allocated).ti,ab. | 603857 |
| 47 | (controlled adj7 (study or design or trial)).ti,ab. | 629900 |
| 48 | (volunteer or volunteers).ti,ab. | 321582 |
| 49 | human experiment/ | 724420 |
| 50 | trial.t i. | 556642 |
| 51 | or/32-50 | 11599287 |
| 52 | (random*adjsampl*adj7 ("cross section"*orquestionnaire*1 or survey*ordatabase*1)).ti,ab. not (comparative study/ or controlled study/ or randomi?ed controlled.ti,ab. or randomly assigned.ti,ab.) | 10647 |
| 53 | exp Cross-Sectional Studies/ not (exp randomized controlled trial/ or controlled clinical trial/ or controlled study/ or randomi?ed controlled.ti,ab. or control group$1.ti,ab.) | 461353 |
| 54 | (((case adj control)*andrandom*) not randomized controlled).ti,ab. | 25359 |
| 55 | systematic review.ti,ab. not (trial or study).ti. | 417381 |
| 56 | (nonrandom*notrandom*).ti,ab. | 21247 |
| 57 | "random field$".ti,ab. | 3206 |
| 58 | (random cluster adj3 sampl$).ti,ab. | 1789 |
| 59 | (review.ab. and review.pt.) not trial.ti. | 1315713 |
| 60 | "we searched".ab. and (review.ti. or review.pt.) | 59369 |
| 61 | "update review".ab. | 154 |
| 62 | (databases adj4 searched).ab. | 78241 |
| 63 | (rat or rats or mouse or mice or swine or porcine or murine or sheep or lambs or pigs or piglets or rabbit or rabbits or cat or cats or dog or dogs or cattle or bovine or monkey or monkeys or trout or marmoset$1).ti. and animal experiment/ | 1312740 |
| 64 | animal experiment/ not (human experiment/ or human/) | 2772090 |
| 65 | or/52-64 | 4957417 |
| 66 | 51 not 65 | 10139651 |
| 67 | 31 and 66 | 2220 |

## Web of Science Core Collection (WoSCC)

TS=(centarian* or centenarian* or elder* or eldest or frail* or geriatric* or nonagenarian* or octogenarian* or octogenarian* or “old age*” or “older adult” or “older age*” or “older female*” or “older male*” or “older man” or “older men” or “older age*” or “older female*” or “older male*” or “older man” or “older men” or “older patient*” or “older people” or “older person*” or “older population” or “older subject*” or “older woman” or “older women” or “oldest old*” or senior* or senium or septuagenarian* or supercentenarian* or “very old*”) AND TS=(caries or carious or decay* or “white spot*” or dmf or dmft or dmfs or periodonti* or periodontal or gingiviti* or gingiva* or “periodontal index*” or “periodontal indices” or “periodontal disease index*” or “periodontal disease indices” or CPI or CPITN or “gingival index*” or “gingival indices” or “bleeding index*” or “bleeding indices” or “bleeding on probing” or BOP or “probing depth*” or “pocket depth*” or “attachment level*” or “attachment loss” or “oral hygiene index*” or “oral hygiene indices” or “plaque index*” or “plaque indices” or “tooth loss*” or “missing teeth” or “partial edentulism” or “patrial tooth loss*” or “dental arch defect*” or “dentition defect*” or “tooth absence” or “edentulous space*” or “dental gap*” or “tooth agenesis or alveolar bone loss*” or “dental prosthesis*” or denture or “fixed partial denture” or “complete denture” or “partial denture” or “removable patrial denture” or overdenture or “dental implant*” or “implant-retained denture” or “implant placement*” or ((oral or mouth* or dental or tooth* or teeth) near/3 (hygiene or health* or care or prevent* or prophyla*)) or mouthwash* or mouthrins* or toothbrush* or toothpaste* or dentifrice* or ((oral or mouth* or dental or tooth* or teeth) near/5 (wash* or rins* or bath* or clean* or brush* or polish* or paste* or foam* or gel*)) or (plaque near/3 (control* or remov*))) AND TS=(("tele health*" or telehealth* or "electronic health*" or ehealth* or "moblie health*" or mhealth* or "digital health*" or "tele care" or telecare) or (mobile* or phone* or telephone* or cellphone* or smartphone* or iphone or ipad or android or pda or app or apps or "smart watch*" or smartwatch* or "smart glasses" or smartglasses or "smart bracelet*" or "smart band*" or smartband*) or ((mobile* or smart* or portable or pocket* or handheld or "hand held" or palm* or tablet*) near/3 (device* or computer* or microcomputer* or pc)) or ((mobile* or smart* or portable or pocket* or handheld or "hand held" or palm* or wireless or electronic or digital or online) near/3 (assistant* or tablet*)) or ((mobile* or smart* or portable or pocket* or handheld or "hand held" or palm* or wireless or electronic or digital or online or web or website or site or internet or tablet*) near/15 (application* or program* or software or platform or algorithm* or messag* or track* or reminder* or video*)) or (social near/3 (media or network* or web or website or site or platform)) or (baidu or bilibili or blog* or facebook or flickr or gagahi or google* or gtalk or hashtag or hellotalk or icq or instagram or kakaotalk or kik or kuaishou or linkedin or metaverse or msn or myspace or oicq or paltalk or pinterest or qq or quora or reddit or sina or skout or skype or snapchat or sohu or sweetalk or telegram or tencent or tiktok or tinder or triller or tumblr or twitter or viber or vine or wechat or weibo or weixin or whatsapp or worldtalk or yahoo or youku or youtube or zhihu or zorpia)) NOT PMID=(1* or 2* or 3* or 4* or 5* or 6* or 7* or 8* or 9*)

Results = 261

## Korea Citation Index-Korean Journal Database (KCI-KJD)

TS=(centarian* or centenarian* or elder* or eldest or frail* or geriatric* or nonagenarian* or octogenarian* or octogenarian* or “old age*” or “older adult” or “older age*” or “older female*” or “older male*” or “older man” or “older men” or “older age*” or “older female*” or “older male*” or “older man” or “older men” or “older patient*” or “older people” or “older person*” or “older population” or “older subject*” or “older woman” or “older women” or “oldest old*” or senior* or senium or septuagenarian* or supercentenarian* or “very old*”) AND TS=(caries or carious or decay* or “white spot*” or dmf or dmft or dmfs or periodonti* or periodontal or gingiviti* or gingiva* or “periodontal index*” or “periodontal indices” or “periodontal disease index*” or “periodontal disease indices” or CPI or CPITN or “gingival index*” or “gingival indices” or “bleeding index*” or “bleeding indices” or “bleeding on probing” or BOP or “probing depth*” or “pocket depth*” or “attachment level*” or “attachment loss” or “oral hygiene index*” or “oral hygiene indices” or “plaque index*” or “plaque indices” or “tooth loss*” or “missing teeth” or “partial edentulism” or “patrial tooth loss*” or “dental arch defect*” or “dentition defect*” or “tooth absence” or “edentulous space*” or “dental gap*” or “tooth agenesis or alveolar bone loss*” or “dental prosthesis*” or denture or “fixed partial denture” or “complete denture” or “partial denture” or “removable patrial denture” or overdenture or “dental implant*” or “implant-retained denture” or “implant placement*” or ((oral or mouth* or dental or tooth* or teeth) near/3 (hygiene or health* or care or prevent* or prophyla*)) or mouthwash* or mouthrins* or toothbrush* or toothpaste* or dentifrice* or ((oral or mouth* or dental or tooth* or teeth) near/5 (wash* or rins* or bath* or clean* or brush* or polish* or paste* or foam* or gel*)) or (plaque near/3 (control* or remov*))) AND TS=(("tele health*" or telehealth* or "electronic health*" or ehealth* or "moblie health*" or mhealth* or "digital health*" or "tele care" or telecare) or (mobile* or phone* or telephone* or cellphone* or smartphone* or iphone or ipad or android or pda or app or apps or "smart watch*" or smartwatch* or "smart glasses" or smartglasses or "smart bracelet*" or "smart band*" or smartband*) or ((mobile* or smart* or portable or pocket* or handheld or "hand held" or palm* or tablet*) near/3 (device* or computer* or microcomputer* or pc)) or ((mobile* or smart* or portable or pocket* or handheld or "hand held" or palm* or wireless or electronic or digital or online) near/3 (assistant* or tablet*)) or ((mobile* or smart* or portable or pocket* or handheld or "hand held" or palm* or wireless or electronic or digital or online or web or website or site or internet or tablet*) near/15 (application* or program* or software or platform or algorithm* or messag* or track* or reminder* or video*)) or (social near/3 (media or network* or web or website or site or platform)) or (baidu or bilibili or blog* or facebook or flickr or gagahi or google* or gtalk or hashtag or hellotalk or icq or instagram or kakaotalk or kik or kuaishou or linkedin or metaverse or msn or myspace or oicq or paltalk or pinterest or qq or quora or reddit or sina or skout or skype or snapchat or sohu or sweetalk or telegram or tencent or tiktok or tinder or triller or tumblr or twitter or viber or vine or wechat or weibo or weixin or whatsapp or worldtalk or yahoo or youku or youtube or zhihu or zorpia))

Results = 60

## Scientific Electronic Library Online (SciELO)

TS=(centarian* or centenarian* or elder* or eldest or frail* or geriatric* or nonagenarian* or octogenarian* or octogenarian* or “old age*” or “older adult” or “older age*” or “older female*” or “older male*” or “older man” or “older men” or “older age*” or “older female*” or “older male*” or “older man” or “older men” or “older patient*” or “older people” or “older person*” or “older population” or “older subject*” or “older woman” or “older women” or “oldest old*” or senior* or senium or septuagenarian* or supercentenarian* or “very old*”) AND TS=(caries or carious or decay* or “white spot*” or dmf or dmft or dmfs or periodonti* or periodontal or gingiviti* or gingiva* or “periodontal index*” or “periodontal indices” or “periodontal disease index*” or “periodontal disease indices” or CPI or CPITN or “gingival index*” or “gingival indices” or “bleeding index*” or “bleeding indices” or “bleeding on probing” or BOP or “probing depth*” or “pocket depth*” or “attachment level*” or “attachment loss” or “oral hygiene index*” or “oral hygiene indices” or “plaque index*” or “plaque indices” or “tooth loss*” or “missing teeth” or “partial edentulism” or “patrial tooth loss*” or “dental arch defect*” or “dentition defect*” or “tooth absence” or “edentulous space*” or “dental gap*” or “tooth agenesis or alveolar bone loss*” or “dental prosthesis*” or denture or “fixed partial denture” or “complete denture” or “partial denture” or “removable patrial denture” or overdenture or “dental implant*” or “implant-retained denture” or “implant placement*” or ((oral or mouth* or dental or tooth* or teeth) near/3 (hygiene or health* or care or prevent* or prophyla*)) or mouthwash* or mouthrins* or toothbrush* or toothpaste* or dentifrice* or ((oral or mouth* or dental or tooth* or teeth) near/5 (wash* or rins* or bath* or clean* or brush* or polish* or paste* or foam* or gel*)) or (plaque near/3 (control* or remov*))) AND TS=(("tele health*" or telehealth* or "electronic health*" or ehealth* or "moblie health*" or mhealth* or "digital health*" or "tele care" or telecare) or (mobile* or phone* or telephone* or cellphone* or smartphone* or iphone or ipad or android or pda or app or apps or "smart watch*" or smartwatch* or "smart glasses" or smartglasses or "smart bracelet*" or "smart band*" or smartband*) or ((mobile* or smart* or portable or pocket* or handheld or "hand held" or palm* or tablet*) near/3 (device* or computer* or microcomputer* or pc)) or ((mobile* or smart* or portable or pocket* or handheld or "hand held" or palm* or wireless or electronic or digital or online) near/3 (assistant* or tablet*)) or ((mobile* or smart* or portable or pocket* or handheld or "hand held" or palm* or wireless or electronic or digital or online or web or website or site or internet or tablet*) near/15 (application* or program* or software or platform or algorithm* or messag* or track* or reminder* or video*)) or (social near/3 (media or network* or web or website or site or platform)) or (baidu or bilibili or blog* or facebook or flickr or gagahi or google* or gtalk or hashtag or hellotalk or icq or instagram or kakaotalk or kik or kuaishou or linkedin or metaverse or msn or myspace or oicq or paltalk or pinterest or qq or quora or reddit or sina or skout or skype or snapchat or sohu or sweetalk or telegram or tencent or tiktok or tinder or triller or tumblr or twitter or viber or vine or wechat or weibo or weixin or whatsapp or worldtalk or yahoo or youku or youtube or zhihu or zorpia))

Results = 60

## SinoMed-Chinese BioMedical Literature Database (CBM)

1. “老人”[常用字段] OR “老年”[常用字段] OR “老年人”[常用字段] OR “老年人群”[常用字段] OR “高龄老人”[常用字段] OR “老年护理”[常用字段]

2. “口腔”[常用字段] OR “牙”[常用字段] OR “齿“[常用字段]

3. “手机应用”[常用字段] OR “移动应用”[常用字段] OR “手机软件”[常用字段] OR ”移动软件”[常用字段] OR “智能手机”[常用字段] OR “平板”[常用字段] OR "app"[常用字段] OR "应用程序"[常用字段]

4. “随机”[常用字段]

5. #1 and #2 and #3 and #4

Results = 4

## Airiti Library

((([ALL4]:(“老人” OR “老年” OR “老年人” OR “老年人群” OR “高齡老人” OR “老年護理”) AND [ALL4]:(“口腔” OR “牙” OR “齒”)) AND [ALL4]:(“手機應用” OR “移動應用” OR “手機軟件” OR “移動軟件” OR “智能手機” OR “平板” OR "app" OR "應用程序")) AND [ALL4]:(“隨機”))

Results = 0

## WHO Global Index Medicus

tw:("centarian* or centenarian* or elder* or eldest or frail* or geriatric* or nonagenarian* or octogenarian* or octogenarian* or “old age” or “older adult” or “older adults” or “older age” or “older age-related diseases” or “older female” or “older females” or “older male” or “older males” or “older man” or “older men” or “older patient” or “older patients” or “older people” or “older person” or “older persons” or “older population” or “older subject” or “older subjects” or “older woman” or “older women” or “oldest old” or senior* or senium or septuagenarian* or supercentenarian* or “very old”")

AND tw:("caries or carious or decay* or “white spot” or “white spots”or dmf or dmft or dmfs or periodonti* or periodontal or gingiviti* or gingiva* or “periodontal index” or “periodontal indexing” or “periodontal indices” or “periodontal disease index” or “periodontal disease indexing” or“periodontal disease indices” or CPI or CPITN or “gingival index” or “gingival indexing” or “gingival indices” or “bleeding index” or “bleeding indexing” or “bleeding indices” or “bleeding on probing” or BOP or “probing depth” or “probing depths” or “pocket depth” or “pocket depths” or “attachment level” or “attachment levels” or “attachment loss” or “oral hygiene index” or “oral hygiene indexing” or “oral hygiene indices” or “plaque index” or “plaque indexing” or “plaque indices” or “tooth loss” or “tooth losses” or “missing teeth” or “partial edentulism” or “patrial tooth loss” or “patrial tooth losses” or “dental arch defect” or “dental arch defects” or “dentition defect” or “dentition defects” or “tooth absence” or “edentulous space” or “edentulous spaces” or “dental gaps” or “dental gap” or “tooth agenesis or alveolar bone loss” or “tooth agenesis or alveolar bone losses” or “dental prosthesis” or “dental prostheses” or denture or “fixed partial denture” or “complete denture” or “partial denture” or “removable patrial denture” or overdenture or “dental implant” or “dental implants” or “implant-retained denture” or “implant placement” or “implant placements” or ((oral or mouth* or dental or tooth* or teeth) NEAR/3 (hygiene or health* or care or prevent* or prophyla*)) or mouthwash* or mouthrins* or toothbrush* or toothpaste* or dentifrice* or ((oral or mouth* or dental or tooth* or teeth) NEAR/5 (wash* or rins* or bath* or clean* or brush* or polish* or paste* or foam* or gel*)) or (plaque NEAR/3 (control* or remov*))")) AND tw:("("tele health" or "tele healths" or telehealth* or "electronic health" or "electronic healths" or ehealth* or "moblie health" or "moblie healths" or mhealth* or "digital health" or "digital healths" or "tele care" or telecare) or (mobile* or phone* or telephone* or cellphone* or smartphone* or iphone or ipad or android or pda or app or apps or "smart watch" or "smart watches" or smartwatch* or "smart glasses" or smartglasses or "smart bracelet" or "smart bracelets" or "smart band" or "smart bands" or smartband*) or ((mobile* or smart* or portable or pocket* or handheld or "hand held" or palm* or tablet*) NEAR/3 (device* or computer* or microcomputer* or pc)) or ((mobile* or smart* or portable or pocket* or handheld or "hand held" or palm* or wireless or electronic or digital or online) NEAR/3 (assistant* or tablet*)) or ((mobile* or smart* or portable or pocket* or handheld or "hand held" or palm* or wireless or electronic or digital or online or web or website or site or internet or tablet*) NEAR/15 (application* or program* or software or platform or algorithm* or messag* or track* or reminder* or video*)) or (social NEAR/3 (media or network* or web or website or site or platform)) or (baidu or bilibili or blog* or facebook or flickr or gagahi or google* or gtalk or hashtag or hellotalk or icq or instagram or kakaotalk or kik or kuaishou or linkedin or metaverse or msn or myspace or oicq or paltalk or pinterest or qq or quora or reddit or sina or skout or skype or snapchat or sohu or sweetalk or telegram or tencent or tiktok or tinder or triller or tumblr or twitter or viber or vine or wechat or weibo or weixin or whatsapp or worldtalk or yahoo or youku or youtube or zhihu or zorpia)"))

Results = 0

## WHO International Clinical Trials Registry Platform (ICTRP)

Advanced Search:

**·** (dental OR oral OR mouth* OR tooth* OR teeth OR caries OR periodonti* OR periodontal OR gingiviti* OR gingiva* OR “tooth loss*” OR “dental arch defect*” OR “edentulous space*”) in the Condition; AND

**·** (mobile* OR phone* OR telephone* OR cellphone* OR iphone OR ipad OR android OR pda OR app OR apps OR smart* OR portable OR pocket* OR handheld OR "hand held" OR palm* OR wireless OR electronic OR digital OR online OR baidu OR bilibili OR blog* OR facebook OR flickr OR gagahi OR google* OR gtalk OR hashtag OR hellotalk OR icq OR instagram OR kakaotalk OR kik OR kuaishou OR linkedin OR metaverse OR msn OR myspace OR oicq OR paltalk OR pinterest OR qq OR quora OR reddit OR sina OR skout OR skype OR snapchat OR sohu OR sweetalk OR telegram OR tencent OR tiktok OR tinder OR triller OR tumblr OR twitter OR viber OR vine OR wechat OR weibo OR weixin OR whatsapp OR worldtalk OR yahoo OR youku OR youtube OR zhihu OR zorpia) in the Intervention; AND

**·** Recruitment status is ALL

Results = 1183

## Open Dissertations

S1. SU (centarian* or centenarian* or elder* or eldest or frail* or geriatric* or nonagenarian* or octogenarian* or octogenarian* or old age* or older adult or older age* or older female* or older male* or older man or older men or older age* or older female* or older male* or older man or older men or older patient* or older people or older person* or older population or older subject* or older woman or older women or oldest old* or senior* or senium or septuagenarian* or supercentenarian* or very old*)

S2. SU (caries or carious or decay* or white spot* or dmf or dmft or dmfs or periodonti* or periodontal or gingiviti* or gingiva* or periodontal index* or periodontal indices or periodontal disease index* or periodontal disease indices or CPI or CPITN or gingival index* or gingival indices or bleeding index* or bleeding indices or bleeding on probing or BOP or probing depth* or pocket depth* or attachment level* or attachment loss or oral hygiene index* or oral hygiene indices or plaque index* or plaque indices or tooth loss* or missing teeth or partial edentulism or patrial tooth loss* or dental arch defect* or dentition defect* or tooth absence or edentulous space* or dental gap* or tooth agenesis or alveolar bone loss* or dental prosthesis* or denture or fixed partial denture or complete denture or partial denture or removable patrial denture or overdenture or dental implant* or implant-retained denture or implant placement* or ((oral or mouth* or dental or tooth* or teeth) N/3 (hygiene or health* or care or prevent* or prophyla*)) or mouthwash* or mouthrins* or toothbrush* or toothpaste* or dentifrice* or ((oral or mouth* or dental or tooth* or teeth) N/5 (wash* or rins* or bath* or clean* or brush* or polish* or paste* or foam* or gel*)) or (plaque N/3 (control* or remov*)))

S3. SU ((tele health* or telehealth* or electronic health* or ehealth* or moblie health* or mhealth* or digital health* or tele care or telecare) or (mobile* or phone* or telephone* or cellphone* or smartphone* or iphone or ipad or android or pda or app or apps or smart watch* or smartwatch* or smart glasses or smartglasses or smart bracelet* or smart band* or smartband*) or ((mobile* or smart* or portable or pocket* or handheld or hand held or palm* or tablet*) N/3 (device* or computer* or microcomputer* or pc)) or ((mobile* or smart* or portable or pocket* or handheld or hand held or palm* or wireless or electronic or digital or online) N/3 (assistant* or tablet*)) or ((mobile* or smart* or portable or pocket* or handheld or hand held or palm* or wireless or electronic or digital or online or web or website or site or internet or tablet*) N/15 (application* or program* or software or platform or algorithm* or messag* or track* or reminder* or video*)) or (social N/3 (media or network* or web or website or site or platform)) or (baidu or bilibili or blog* or facebook or flickr or gagahi or google* or gtalk or hashtag or hellotalk or icq or instagram or kakaotalk or kik or kuaishou or linkedin or metaverse or msn or myspace or oicq or paltalk or pinterest or qq or quora or reddit or sina or skout or skype or snapchat or sohu or sweetalk or telegram or tencent or tiktok or tinder or triller or tumblr or twitter or viber or vine or wechat or weibo or weixin or whatsapp or worldtalk or yahoo or youku or youtube or zhihu or zorpia)))

S4. S1 AND S2 AND S3

Results = 7

## Web of Science ProQuest Dissertations & Theses (PQDT)

TS=(centarian* or centenarian* or elder* or eldest or frail* or geriatric* or nonagenarian* or octogenarian* or octogenarian* or “old age*” or “older adult” or “older age*” or “older female*” or “older male*” or “older man” or “older men” or “older age*” or “older female*” or “older male*” or “older man” or “older men” or “older patient*” or “older people” or “older person*” or “older population” or “older subject*” or “older woman” or “older women” or “oldest old*” or senior* or senium or septuagenarian* or supercentenarian* or “very old*”) AND TS=(caries or carious or decay* or “white spot*” or dmf or dmft or dmfs or periodonti* or periodontal or gingiviti* or gingiva* or “periodontal index*” or “periodontal indices” or “periodontal disease index*” or “periodontal disease indices” or CPI or CPITN or “gingival index*” or “gingival indices” or “bleeding index*” or “bleeding indices” or “bleeding on probing” or BOP or “probing depth*” or “pocket depth*” or “attachment level*” or “attachment loss” or “oral hygiene index*” or “oral hygiene indices” or “plaque index*” or “plaque indices” or “tooth loss*” or “missing teeth” or “partial edentulism” or “patrial tooth loss*” or “dental arch defect*” or “dentition defect*” or “tooth absence” or “edentulous space*” or “dental gap*” or “tooth agenesis or alveolar bone loss*” or “dental prosthesis*” or denture or “fixed partial denture” or “complete denture” or “partial denture” or “removable patrial denture” or overdenture or “dental implant*” or “implant-retained denture” or “implant placement*” or ((oral or mouth* or dental or tooth* or teeth) near/3 (hygiene or health* or care or prevent* or prophyla*)) or mouthwash* or mouthrins* or toothbrush* or toothpaste* or dentifrice* or ((oral or mouth* or dental or tooth* or teeth) near/5 (wash* or rins* or bath* or clean* or brush* or polish* or paste* or foam* or gel*)) or (plaque near/3 (control* or remov*))) AND TS=(("tele health*" or telehealth* or "electronic health*" or ehealth* or "moblie health*" or mhealth* or "digital health*" or "tele care" or telecare) or (mobile* or phone* or telephone* or cellphone* or smartphone* or iphone or ipad or android or pda or app or apps or "smart watch*" or smartwatch* or "smart glasses" or smartglasses or "smart bracelet*" or "smart band*" or smartband*) or ((mobile* or smart* or portable or pocket* or handheld or "hand held" or palm* or tablet*) near/3 (device* or computer* or microcomputer* or pc)) or ((mobile* or smart* or portable or pocket* or handheld or "hand held" or palm* or wireless or electronic or digital or online) near/3 (assistant* or tablet*)) or ((mobile* or smart* or portable or pocket* or handheld or "hand held" or palm* or wireless or electronic or digital or online or web or website or site or internet or tablet*) near/15 (application* or program* or software or platform or algorithm* or messag* or track* or reminder* or video*)) or (social near/3 (media or network* or web or website or site or platform)) or (baidu or bilibili or blog* or facebook or flickr or gagahi or google* or gtalk or hashtag or hellotalk or icq or instagram or kakaotalk or kik or kuaishou or linkedin or metaverse or msn or myspace or oicq or paltalk or pinterest or qq or quora or reddit or sina or skout or skype or snapchat or sohu or sweetalk or telegram or tencent or tiktok or tinder or triller or tumblr or twitter or viber or vine or wechat or weibo or weixin or whatsapp or worldtalk or yahoo or youku or youtube or zhihu or zorpia))

Results = 254

## Web of Science Preprint Citation Index

TS=(centarian* or centenarian* or elder* or eldest or frail* or geriatric* or nonagenarian* or octogenarian* or octogenarian* or “old age*” or “older adult” or “older age*” or “older female*” or “older male*” or “older man” or “older men” or “older age*” or “older female*” or “older male*” or “older man” or “older men” or “older patient*” or “older people” or “older person*” or “older population” or “older subject*” or “older woman” or “older women” or “oldest old*” or senior* or senium or septuagenarian* or supercentenarian* or “very old*”) AND TS=(caries or carious or decay* or “white spot*” or dmf or dmft or dmfs or periodonti* or periodontal or gingiviti* or gingiva* or “periodontal index*” or “periodontal indices” or “periodontal disease index*” or “periodontal disease indices” or CPI or CPITN or “gingival index*” or “gingival indices” or “bleeding index*” or “bleeding indices” or “bleeding on probing” or BOP or “probing depth*” or “pocket depth*” or “attachment level*” or “attachment loss” or “oral hygiene index*” or “oral hygiene indices” or “plaque index*” or “plaque indices” or “tooth loss*” or “missing teeth” or “partial edentulism” or “patrial tooth loss*” or “dental arch defect*” or “dentition defect*” or “tooth absence” or “edentulous space*” or “dental gap*” or “tooth agenesis or alveolar bone loss*” or “dental prosthesis*” or denture or “fixed partial denture” or “complete denture” or “partial denture” or “removable patrial denture” or overdenture or “dental implant*” or “implant-retained denture” or “implant placement*” or ((oral or mouth* or dental or tooth* or teeth) near/3 (hygiene or health* or care or prevent* or prophyla*)) or mouthwash* or mouthrins* or toothbrush* or toothpaste* or dentifrice* or ((oral or mouth* or dental or tooth* or teeth) near/5 (wash* or rins* or bath* or clean* or brush* or polish* or paste* or foam* or gel*)) or (plaque near/3 (control* or remov*))) AND TS=(("tele health*" or telehealth* or "electronic health*" or ehealth* or "moblie health*" or mhealth* or "digital health*" or "tele care" or telecare) or (mobile* or phone* or telephone* or cellphone* or smartphone* or iphone or ipad or android or pda or app or apps or "smart watch*" or smartwatch* or "smart glasses" or smartglasses or "smart bracelet*" or "smart band*" or smartband*) or ((mobile* or smart* or portable or pocket* or handheld or "hand held" or palm* or tablet*) near/3 (device* or computer* or microcomputer* or pc)) or ((mobile* or smart* or portable or pocket* or handheld or "hand held" or palm* or wireless or electronic or digital or online) near/3 (assistant* or tablet*)) or ((mobile* or smart* or portable or pocket* or handheld or "hand held" or palm* or wireless or electronic or digital or online or web or website or site or internet or tablet*) near/15 (application* or program* or software or platform or algorithm* or messag* or track* or reminder* or video*)) or (social near/3 (media or network* or web or website or site or platform)) or (baidu or bilibili or blog* or facebook or flickr or gagahi or google* or gtalk or hashtag or hellotalk or icq or instagram or kakaotalk or kik or kuaishou or linkedin or metaverse or msn or myspace or oicq or paltalk or pinterest or qq or quora or reddit or sina or skout or skype or snapchat or sohu or sweetalk or telegram or tencent or tiktok or tinder or triller or tumblr or twitter or viber or vine or wechat or weibo or weixin or whatsapp or worldtalk or yahoo or youku or youtube or zhihu or zorpia))

Results = 7

# Appendix B: Risk of bias assessment tools -RoB-2 tool (Revised Cochrane risk-of-bias tool for randomized trials)

**Signalling questions**

**Domain 1: Randomization process**

1.1 Was the allocation sequence random?

1.2 Was the allocation sequence concealed until participants were enrolled and assigned to interventions?

1.3 Did baseline differences between intervention groups suggest a problem with the randomization process?

Optional: What is the predicted direction of bias arising from the randomization process?

**Domain 2: Deviations from intended interventions** **(*effect of assignment to intervention*)**

2.1. Were participants aware of their assigned intervention during the trial?

2.2. Were carers and people delivering the interventions aware of participants' assigned

intervention during the trial?

2.3. If Y/PY/NI to 2.1 or 2.2: Were there deviations from the intended intervention that arose because of the trial context?

2.4 If Y/PY to 2.3: Were these deviations likely to have affected the outcome?

2.5. If Y/PY/NI to 2.4: Were these deviations from intended intervention balanced between groups?

2.6 Was an appropriate analysis used to estimate the effect of assignment to intervention?

2.7 If N/PN/NI to 2.6: Was there potential for a substantial impact (on the result) of the failure to analyse participants in the group to which they were randomized?

Optional: What is the predicted direction of bias due to deviations from intended interventions?

**Domain 2: Deviations from intended interventions (*effect of adhering to intervention*)**

2.1. Were participants aware of their assigned intervention during the trial?

2.2. Were carers and people delivering the interventions aware of participants' assigned intervention during the trial?

2.3. [If applicable:] If Y/PY/NI to 2.1 or 2.2: Were important non-protocol interventions balanced across intervention groups?

2.4. [If applicable:] Were there failures in implementing the intervention that could have affected the outcome?

2.5. [If applicable:] Was there non-adherence to the assigned intervention regimen that could have affected participants’ outcomes?

2.6. If N/PN/NI to 2.3, or Y/PY/NI to 2.4 or 2.5: Was an appropriate analysis used to estimate the effect of adhering to intervention?

Optional: What is the predicted direction of bias due to deviations from intended interventions?

**Domain 3: Missing outcome data**

3.1 Were data for this outcome available for all, or nearly all, participants randomized?

3.2 If N/PN/NI to 3.1: Is there evidence that the result was not biased by missing outcome data?

3.3 If N/PN to 3.2: Could missingness in the outcome depend on its true value?

3.4 If Y/PY/NI to 3.3: Is it likely that missingness in the outcome depended on its true value?

Optional: What is the predicted direction of bias due to missing outcome data?

**Domain 4: Measurement of the outcome**

4.1 Was the method of measuring the outcome inappropriate?

4.2 Could measurement or ascertainment of the outcome have differed between intervention groups?

4.3 If N/PN/NI to 4.1 and 4.2: Were outcome assessors aware of the intervention received by study participants?

4.4 If Y/PY/NI to 4.3: Could assessment of the outcome have been influenced by knowledge of intervention received?

4.5 If Y/PY/NI to 4.4: Is it likely that assessment of the outcome was influenced by knowledge of intervention received?

Optional: What is the predicted direction of bias in measurement of the outcome?

**Domain 5: Selection of the reported result**

5.1 Were the data that produced this result analysed in accordance with a pre-specified analysis plan that was finalized before unblinded outcome data were available for analysis?

5.2. Is the numerical result being assessed likely to have been selected, on the basis of the results, from multiple eligible outcome measurements (e.g. scales, definitions, time points) within the outcome domain?

5.3 Is the numerical result being assessed likely to have been selected, on the basis of the results, from multiple eligible analyses of the data?

Optional: What is the predicted direction of bias due to selection of the reported result?

**Reference**

Sterne JAC, Savović J, Page MJ, Elbers RG, Blencowe NS, Boutron I, Cates CJ, Cheng H-Y, Corbett MS, Eldridge SM, Hernán MA, Hopewell S, Hróbjartsson A, Junqueira DR, Jüni P, Kirkham JJ, Lasserson T, Li T, McAleenan A, Reeves BC, Shepperd S, Shrier I, Stewart LA, Tilling K, White IR, Whiting PF, Higgins JPT. RoB 2: a revised tool for assessing risk of bias in randomised trials.*BMJ* 2019; 366: l4898.

# Appendix C: Excluded studies and Reason for Exclusion (8 studies with 8 reports)

| Study ID | References | Reason for exclusion |
| --- | --- | --- |
| Melbye 2023 | Melbye EL, Bull VH, Hidle KS Assessment of the SmartJournal Intervention for Improved Oral Care in Nursing Homes: Protocol for a Cluster Randomized Controlled Trial JMIR Res Protoc 2023;12:e46926 | Ongoing studies |
| Veynachter 2023 | Veynachter T, Sural Y, Baudot P, *et al*. Diagnostic performance of two teledentistry tools in elderly care facilities: the ONE-1 protocol for a pilot prospective diagnostic study. *BMJ Open* 2023;**13**:e063906. doi:10.1136/ bmjopen-2022-063906 | Ongoing studies |
| Venegas 2022 | Venegas B, Rueda S, Flores M, Cerda A & Beltrán V. Prevention of oral cancer through the implementation of a teledentistry platform for the elderly. J Oral Res 2022; S-1 (Seminario Científico):1-8. Doi:10.17126/joralres.2022.027 | Non-interventional study |
| Park 2024 | Park, C., Kim, N., & Oh, J. (2024). Relationship between digital oral health literacy and digital device utilization among adults in Gangwon-do. Journal of Korean Society of Dental Hygiene. | Non-interventional study |
| Beltrán 2022 | Beltrán V, Acuña-Mardones P, Díaz J, Alvarado E & von Marttens A. TEGO: A new concept of teledentistry for the elderly through a web platform and mobile app in the context of the covid-19 pandemic. J Oral Res 2022; S-1 (Seminario Científico):1-8. Doi:10.17126/joralres.2022.023 | The outcome indicator is not applicable. |
| Sequeira 2010 | Sequeira, É. (2009). Aplicação de modelo educacional interativo como recurso para orientação e motivação sobre saúde oral em idosos. Tese de Doutorado, Faculdade de Medicina, Universidade de São Paulo, São Paulo. doi:10.11606/T.5.2009.tde-07122009-164644. Recuperado em 2025-09-12, de www.teses.usp.br | Cohort studies |
| Tynan 2018 | Tynan, A., Deeth, L., & McKenzie, D. (2018). An integrated oral health program for rural residential aged care facilities: a mixed methods comparative study. *BMC health services research*, *18*(1), 515. https://doi.org/10.1186/s12913-018-3321-5 | intervention of oral health promotion not only using mobile applications |
| Sepúlveda 2023 | Oral Health Promotion Mediated by Teledentistry in Community-dwelling Older Adults of La Araucan&iacute;a Region, Chile | Ongoing studies |

|  |  |  |
| --- | --- | --- |

# Appendix D: Details of the included studies (9 studies with 9 reports)

| Author (Year), Country | Participants | Experimental Groups | Follow-up | Main and Secondary Outcomes from Primary Studies |
| --- | --- | --- | --- | --- |
| Jade Yi Ming Ng (2021), Malaysia | Patients aged 25–75 years who wore cobalt–chromium RPD, had been using a smartphone with Internet access within the last 6 months, and are literate in English or Malay language. 13 over 65 years old, 39 below 65 years old | **Intervention:**  removable partial denture wearers received education using the PWA  **Control:**  removable partial denture wearers received verbal instructions accompanied by demonstration of hygienic procedures. | 1 mo. 3 mo.  Asynchronous and synchronous | **Main:**  Oral health-related knowledge. PI. GI. DP score |
| Ji-Yun Ki (2021), Korea | Elderly people aged 65 years, understanding of the content and purpose of the study, voluntary agreement to participate, ability to communicate without linguistic, auditory, or visual impairment, and normal cognitive ability | **Intervention:**  received the OHEMA intervention for 6 weeks  **Control:**  did not undergo any oral hygiene education or OHEMA intervention | 6 wk.  Asynchronous | **Main:**  USFR. Tongue pressure  **Secondary:**  Subjective oral dryness.  SWAL-QoL |
| Kyeong Hee Lee (2021), Korea | Adults aged 65 years or older who owned a smartphone, had no communication problems, and were able to read and understand Korean, enrolled at a senior college and senior welfare center in Gyeonggi Province, Korea | **App use:**  Oral health education was provided using the smartphone app developed in this study for five weeks  **Non-app use:**  received lecture-based oral health education using PowerPoint slides at the affiliated facility twice per week for five weeks  **Control:**  did not undergo any oral health education | 5 wk.  Asynchronous | **Main:**  Oral health-related knowledge. O’Leary index. Winkel tongue coating index. GI  **Secondary:**  OHIP-14. GOHAI |
| Eun Seo Jung (2021), Korea | Individuals aged 65 or above, registered in a senior college and senior welfare center in the Gyeonggi Province, owning a smartphone and living independently | **App use:**  received combined oral and whole- body exercises for 5 weeks  **Non-app use:**  Combined oral and whole- body exercises were performed under the direct guidance of the researchers twice a week for 5 weeks without using the app. Each session lasted for about 1 h.  **Control:**  did not undergo any oral health education | 5 wk.  Asynchronous | **Main:**  USFR. Anterior tongue strength. Posterior tongue strength. Cheek strength  **Secondary:**  OHIP-14. GOHAI |
| Worachate Romalee (2023), Chinese Tiwan | Adults aged ≥ 65 years from six randomly selected activity centers in Beitou District, Taipei, Taiwan—all centers had provided no oral health education to members in the 6 months preceding the study, and participants were proficient in using smartphones/tablets, free of communication barriers, and literate in traditional Chinese. | **App use:**  received the OHEMA intervention for 2 weeks  **Non-app use:**  received a one-hour lecture on oral health, based on typical programs in Taiwan, was conducted by an experienced oral hygienist  **Control:**  did not undergo any oral health education | Immediately. 2 wk.  Asynchronous | **Main:**  Oral healthcare-related knowledge. Winkel tongue coating index. PI. GI. |
| Kristina Wanyonyi (2022), The UK | Patients aged 65 years or older, dentate, fluent in spoken and written English, having a scheduled care plan at UPDA, and willing to provide informed consent and responsibly participate in the study | **Intervention:**  underwent a ten- week long intervention period where three text messages were sent per week  **Control:**  received a leaflet in the post providing the same information contained within the thirty text messages | 6 mo.  Asynchronous and synchronous | **Secondary:**  OHIP-14 |
| Kyeong Hee Lee (2024), Korea | Participants were recruited from senior welfare centers in the Seoul metropolitan area using non-probability sampling methods. We included individuals aged 65 and over who were able to communicate effectively, had sufficient mobility, owned a personal cell phone, and could access content using simple gestures. | **App use:**  received the OHEMA intervention 2 times per week, for 6 weeks, 12 times in total  **Control:**  no intervention | 2 wk.  Asynchronous | **Main:**  USFR. O’Leary index. Winkel tongue coating index. Anterior tongue strength. Posterior tongue strength. Cheek strength. GI.  **Secondary:**  OHIP-14. GOHAI |
| Wan Nor Hayati Wan Abd. Manan (2025), Malaysia | Sixty-two participants who attended the Polyclinic Kulliyyah of Dentistry, IIUM Kuantan in 2022 for removable prosthesis fabrication were recruited. | **App use:**  received a series of instructional home denture care information videos which were made in-house via WhatsApp and in weeks 1, 3, 5, and 7 after denture insertion and before the postinsertion questionnaires.  **Non-app use:**  received instructions at the chairside on denture care in Week 1 and information on the relationship between edentulism and general health in Week 3. | 6-8 wk.  Asynchronous | **Main:**  Oral healthcare-related knowledge. |
| Makiko Nishi (2019), Republic of Ireland | The pre-determined inclusion criteria for patient participants were (1) those who were ready to give consent, (2) 19–70 years of age, (3) medical-card holder (i.e. proxy for economically disadvantaged status; a medical-card holder is entitled to a range of health services free of charge in the RoI), (4) at least 20 teeth present, (5) not pregnant and (6) ability to use text messages. | **App use:**  received 1 confirmation message, 24 personalized messages weekly, 1 reminder message  **Non-app use:**  received 1 confirmation message, 24 non-personalized messages weekly, 1 reminder message | 24 wk.  Asynchronous and synchronous | **Main:**  Chance of avoiding new cavities. |

*Abbreviations*: *PWA* Progressive Web Application, *OHEMA* Oral Health Education Using a Mobile App

^1^App use group referred to intervention using mobile applications, the No-app use group referred to intervention using traditional strategies, and the Control group referred to no intervention being implement

# Appendix E: Risk-of-Bias Assessment of Included Studies (9 studies with 9 reports)

## Jung 2024

| **Outcome** | OHIP-14 | **Results** | SMD, -0.42 | **Weight** | 1 |
| --- | --- | --- | --- | --- | --- |
| **Domain** | **Signalling question** | | | **Response** | **Comments** |
| **Bias arising from the randomization process** | 1.1 Was the allocation sequence random? | | | Y | The selected participants were allocated to non-app use, app use and control groups using a random draw. |
|  | 1.2 Was the allocation sequence concealed until participants were enrolled and assigned to interventions? | | | PY |  |
|  | 1.3 Did baseline differences between intervention groups suggest a problem with the randomization process? | | | N | Table 1 shows no significant differences between groups. |
|  | **Risk of bias judgement** | | | **Low** |  |
| **Bias due to deviations from intended interventions** | 2.1.Were participants aware of their assigned intervention during the trial? | | | Y | Due to the nature of the intervention, blinding of the participants was infeasible. Researchers directly guided the non-app group; app group used self-guided videos. Likely aware. |
|  | 2.2.Were carers and people delivering the interventions aware of participants' assigned intervention during the trial? | | | Y |  |
|  | 2.3. If Y/PY/NI to 2.1 or 2.2: Were there deviations from the intended intervention that arose because of the experimental context? | | | PN | There were no deviations from the intended intervention that arose. |
|  | 2.4 If Y/PY to 2.3: Were these deviations likely to have affected the outcome? | | | NA |  |
|  | 2.5. If Y/PY/NI to 2.4: Were these deviations from intended intervention balanced between groups? | | | NA |  |
|  | 2.6 Was an appropriate analysis used to estimate the effect of assignment to intervention? | | | PY | Analysis appears to follow intention-to-treat principles, as all randomized participants who completed the study were included in final analysis. |
|  | 2.7 If N/PN/NI to 2.6: Was there potential for a substantial impact (on the result) of the failure to analyse participants in the group to which they were randomized? | | | NA |  |
|  | **Risk of bias judgement** | | | **Low** |  |
| **Bias due to missing outcome data** | 3.1 Were data for this outcome available for all, or nearly all, participants randomized? | | | PN | 90 recruited, 76 analyzed (84.4% retention). |
|  | 3.2 If N/PN/NI to 3.1: Is there evidence that result was not biased by missing outcome data? | | | PN | No sensitivity analysis or imputation described. |
|  | 3.3 If N/PN to 3.2: Could missingness in the outcome depend on its true value? | | | PN | The number of lost-to-follow-up cases was similar across groups, and the reasons were also similar. |
|  | 3.4 If Y/PY/NI to 3.3: Is it likely that missingness in the outcome depended on its true value? | | | NA |  |
|  | **Risk of bias judgement** | | | **Low** |  |
| **Bias in measurement of the outcome** | 4.1 Was the method of measuring the outcome inappropriate? | | | N | OHIP-14 is a validated instrument for oral health-related quality of life. |
|  | 4.2 Could measurement or ascertainment of the outcome have differed between intervention groups? | | | N | Self-reported questionnaire, same method for all groups. |
|  | 4.3 Were outcome assessors aware of the intervention received by study participants? | | | Y | OHIP-14 is participant-reported; participants were aware of their group. |
|  | 4.4 If Y/PY/NI to 4.3: Could assessment of the outcome have been influenced by knowledge of intervention received? | | | PY | OHIP-14 could be influenced by awareness of intervention. |
|  | 4.5 If Y/PY/NI to 4.4: Is it likely that assessment of the outcome was influenced by knowledge of intervention received? | | | PY |  |
|  | **Risk of bias judgement** | | | **High** |  |
| **Bias in selection of the reported result** | 5.1 Were the data that produced this result analysed in accordance with a pre-specified analysis plan that was finalized before unblinded outcome data were available for analysis? | | | Y | There was a trial register entry, which provided details of the pre‐specified outcomes. |
|  | 5.2 ... multiple eligible outcome measurements (e.g. scales, definitions, time points) within the outcome domain? | | | N | OHIP-14 only one scale used for perceived oral health. |
|  | 5.3 ... multiple eligible analyses of the data? | | | N | Only paired t-test and ANOVA reported for OHIP-14. No evidence of multiple analysis attempts. |
|  | **Risk of bias judgement** | | | **Low** |  |
| **Overall bias** | **Risk of bias judgement** | | | **High** |  |

Abbreviations: Y = yes, PY = probably yes, PN = probably no, N = no, NI = no information, NA = not applicable

| **Outcome** | GOHAI | **Results** | SMD, -0.42 | | **Weight** | 1 |
| --- | --- | --- | --- | --- | --- | --- |
| **Domain** | **Signalling question** | | | **Response** | | **Comments** |
| **Bias arising from the randomization process** | 1.1 Was the allocation sequence random? | | | Y | | The selected participants were allocated to non-app use, app use and control groups using a random draw. |
|  | 1.2 Was the allocation sequence concealed until participants were enrolled and assigned to interventions? | | | PY | |  |
|  | 1.3 Did baseline differences between intervention groups suggest a problem with the randomization process? | | | N | | Table 1 shows no significant differences between groups. |
|  | **Risk of bias judgement** | | | **Low** | |  |
| **Bias due to deviations from intended interventions** | 2.1.Were participants aware of their assigned intervention during the trial? | | | Y | | Due to the nature of the intervention, blinding of the participants was infeasible. Researchers directly guided the non-app group; app group used self-guided videos. Likely aware. |
|  | 2.2.Were carers and people delivering the interventions aware of participants' assigned intervention during the trial? | | | Y | |  |
|  | 2.3. If Y/PY/NI to 2.1 or 2.2: Were there deviations from the intended intervention that arose because of the experimental context? | | | PN | | There were no deviations from the intended intervention that arose. |
|  | 2.4 If Y/PY to 2.3: Were these deviations likely to have affected the outcome? | | | NA | |  |
|  | 2.5. If Y/PY/NI to 2.4: Were these deviations from intended intervention balanced between groups? | | | NA | |  |
|  | 2.6 Was an appropriate analysis used to estimate the effect of assignment to intervention? | | | PY | | Analysis appears to follow intention-to-treat principles, as all randomized participants who completed the study were included in final analysis. |
|  | 2.7 If N/PN/NI to 2.6: Was there potential for a substantial impact (on the result) of the failure to analyse participants in the group to which they were randomized? | | | NA | |  |
|  | **Risk of bias judgement** | | | **Low** | |  |
| **Bias due to missing outcome data** | 3.1 Were data for this outcome available for all, or nearly all, participants randomized? | | | PN | | 90 recruited, 76 analyzed (84.4% retention). |
|  | 3.2 If N/PN/NI to 3.1: Is there evidence that result was not biased by missing outcome data? | | | PN | | No sensitivity analysis or imputation described. |
|  | 3.3 If N/PN to 3.2: Could missingness in the outcome depend on its true value? | | | PN | | The number of lost-to-follow-up cases was similar across groups, and the reasons were also similar. |
|  | 3.4 If Y/PY/NI to 3.3: Is it likely that missingness in the outcome depended on its true value? | | | NA | |  |
|  | **Risk of bias judgement** | | | **Low** | |  |
| **Bias in measurement of the outcome** | 4.1 Was the method of measuring the outcome inappropriate? | | | N | | GOHAI is a validated instrument for oral health-related quality of life. |
|  | 4.2 Could measurement or ascertainment of the outcome have differed between intervention groups? | | | N | | Self-reported questionnaire, same method for all groups. |
|  | 4.3 Were outcome assessors aware of the intervention received by study participants? | | | Y | | GOHAI is participant-reported; participants were aware of their group. |
|  | 4.4 If Y/PY/NI to 4.3: Could assessment of the outcome have been influenced by knowledge of intervention received? | | | PY | | GOHAI could be influenced by awareness of intervention. |
|  | 4.5 If Y/PY/NI to 4.4: Is it likely that assessment of the outcome was influenced by knowledge of intervention received? | | | PY | |  |
|  | **Risk of bias judgement** | | | **High** | |  |
| **Bias in selection of the reported result** | 5.1 Were the data that produced this result analysed in accordance with a pre-specified analysis plan that was finalized before unblinded outcome data were available for analysis? | | | Y | | There was a trial register entry, which provided details of the pre‐specified outcomes. |
|  | 5.2 ... multiple eligible outcome measurements (e.g. scales, definitions, time points) within the outcome domain? | | | N | | OHIP-14 only one scale used for perceived oral health. |
|  | 5.3 ... multiple eligible analyses of the data? | | | N | | Only paired t-test and ANOVA reported for OHIP-14. No evidence of multiple analysis attempts. |
|  | **Risk of bias judgement** | | | **Low** | |  |
| **Overall bias** | **Risk of bias judgement** | | | **High** | |  |

Abbreviations: Y = yes, PY = probably yes, PN = probably no, N = no, NI = no information, NA = not applicable

| **Outcome** | USFR | **Results** | SMD, 0.94 | | **Weight** | 1 |
| --- | --- | --- | --- | --- | --- | --- |
| **Domain** | **Signalling question** | | | **Response** | | **Comments** |
| **Bias arising from the randomization process** | 1.1 Was the allocation sequence random? | | | Y | | The selected participants were allocated to non-app use, app use and control groups using a random draw. |
|  | 1.2 Was the allocation sequence concealed until participants were enrolled and assigned to interventions? | | | PY | |  |
|  | 1.3 Did baseline differences between intervention groups suggest a problem with the randomization process? | | | N | | Table 1 shows no significant differences between groups. |
|  | **Risk of bias judgement** | | | **Low** | |  |
| **Bias due to deviations from intended interventions** | 2.1.Were participants aware of their assigned intervention during the trial? | | | Y | | Due to the nature of the intervention, blinding of the participants was infeasible. Researchers directly guided the non-app group; app group used self-guided videos. Likely aware. |
|  | 2.2.Were carers and people delivering the interventions aware of participants' assigned intervention during the trial? | | | Y | |  |
|  | 2.3. If Y/PY/NI to 2.1 or 2.2: Were there deviations from the intended intervention that arose because of the experimental context? | | | PN | | There were no deviations from the intended intervention that arose. |
|  | 2.4 If Y/PY to 2.3: Were these deviations likely to have affected the outcome? | | | NA | |  |
|  | 2.5. If Y/PY/NI to 2.4: Were these deviations from intended intervention balanced between groups? | | | NA | |  |
|  | 2.6 Was an appropriate analysis used to estimate the effect of assignment to intervention? | | | PY | | Analysis appears to follow intention-to-treat principles, as all randomized participants who completed the study were included in final analysis. |
|  | 2.7 If N/PN/NI to 2.6: Was there potential for a substantial impact (on the result) of the failure to analyse participants in the group to which they were randomized? | | | NA | |  |
|  | **Risk of bias judgement** | | | **Low** | |  |
| **Bias due to missing outcome data** | 3.1 Were data for this outcome available for all, or nearly all, participants randomized? | | | PN | | 90 recruited, 76 analyzed (84.4% retention). |
|  | 3.2 If N/PN/NI to 3.1: Is there evidence that result was not biased by missing outcome data? | | | PN | | No sensitivity analysis or imputation described. |
|  | 3.3 If N/PN to 3.2: Could missingness in the outcome depend on its true value? | | | PN | | The number of lost-to-follow-up cases was similar across groups, and the reasons were also similar. |
|  | 3.4 If Y/PY/NI to 3.3: Is it likely that missingness in the outcome depended on its true value? | | | NA | |  |
|  | **Risk of bias judgement** | | | **Low** | |  |
| **Bias in measurement of the outcome** | 4.1 Was the method of measuring the outcome inappropriate? | | | N | | USFR was measured using a standardized, precise electronic scale (SHINKO BJ-420), following a controlled protocol. |
|  | 4.2 Could measurement or ascertainment of the outcome have differed between intervention groups? | | | N | | Same method, time, place, and researcher for all groups. |
|  | 4.3 Were outcome assessors aware of the intervention received by study participants? | | | N | | The paper states: “Blinding was done so that the researchers assessing outcomes did not know which group the participants belonged to.” |
|  | 4.4 If Y/PY/NI to 4.3: Could assessment of the outcome have been influenced by knowledge of intervention received? | | | NA | |  |
|  | 4.5 If Y/PY/NI to 4.4: Is it likely that assessment of the outcome was influenced by knowledge of intervention received? | | | NA | |  |
|  | **Risk of bias judgement** | | | **Low** | |  |
| **Bias in selection of the reported result** | 5.1 Were the data that produced this result analysed in accordance with a pre-specified analysis plan that was finalized before unblinded outcome data were available for analysis? | | | Y | | There was a trial register entry, which provided details of the pre‐specified outcomes. |
|  | 5.2 ... multiple eligible outcome measurements (e.g. scales, definitions, time points) within the outcome domain? | | | N | | OHIP-14 only one scale used for perceived oral health. |
|  | 5.3 ... multiple eligible analyses of the data? | | | N | | Only paired t-test and ANOVA reported for OHIP-14. No evidence of multiple analysis attempts. |
|  | **Risk of bias judgement** | | | **Low** | |  |
| **Overall bias** | **Risk of bias judgement** | | | **Low** | |  |

Abbreviations: Y = yes, PY = probably yes, PN = probably no, N = no, NI = no information, NA = not applicable

| **Outcome** | Posterior tongue strength | **Results** | SMD, 0.09 | **Weight** | 1 |
| --- | --- | --- | --- | --- | --- |
| **Domain** | **Signalling question** | | | **Response** | **Comments** |
| **Bias arising from the randomization process** | 1.1 Was the allocation sequence random? | | | Y | The selected participants were allocated to non-app use, app use and control groups using a random draw. |
|  | 1.2 Was the allocation sequence concealed until participants were enrolled and assigned to interventions? | | | PY |  |
|  | 1.3 Did baseline differences between intervention groups suggest a problem with the randomization process? | | | N | Table 1 shows no significant differences between groups. |
|  | **Risk of bias judgement** | | | **Low** |  |
| **Bias due to deviations from intended interventions** | 2.1.Were participants aware of their assigned intervention during the trial? | | | Y | Due to the nature of the intervention, blinding of the participants was infeasible. Researchers directly guided the non-app group; app group used self-guided videos. Likely aware. |
|  | 2.2.Were carers and people delivering the interventions aware of participants' assigned intervention during the trial? | | | Y |  |
|  | 2.3. If Y/PY/NI to 2.1 or 2.2: Were there deviations from the intended intervention that arose because of the experimental context? | | | PN | There were no deviations from the intended intervention that arose. |
|  | 2.4 If Y/PY to 2.3: Were these deviations likely to have affected the outcome? | | | NA |  |
|  | 2.5. If Y/PY/NI to 2.4: Were these deviations from intended intervention balanced between groups? | | | NA |  |
|  | 2.6 Was an appropriate analysis used to estimate the effect of assignment to intervention? | | | PY | Analysis appears to follow intention-to-treat principles, as all randomized participants who completed the study were included in final analysis. |
|  | 2.7 If N/PN/NI to 2.6: Was there potential for a substantial impact (on the result) of the failure to analyse participants in the group to which they were randomized? | | | NA |  |
|  | **Risk of bias judgement** | | | **Low** |  |
| **Bias due to missing outcome data** | 3.1 Were data for this outcome available for all, or nearly all, participants randomized? | | | PN | 90 recruited, 76 analyzed (84.4% retention). |
|  | 3.2 If N/PN/NI to 3.1: Is there evidence that result was not biased by missing outcome data? | | | PN | No sensitivity analysis or imputation described. |
|  | 3.3 If N/PN to 3.2: Could missingness in the outcome depend on its true value? | | | PN | The number of lost-to-follow-up cases was similar across groups, and the reasons were also similar. |
|  | 3.4 If Y/PY/NI to 3.3: Is it likely that missingness in the outcome depended on its true value? | | | NA |  |
|  | **Risk of bias judgement** | | | **Low** |  |
| **Bias in measurement of the outcome** | 4.1 Was the method of measuring the outcome inappropriate? | | | N | Posterior tongue strength was measured using the Iowa Oral Performance Instrument (IOPI), a validated device for oral muscle strength. |
|  | 4.2 Could measurement or ascertainment of the outcome have differed between intervention groups? | | | N | The same instrument, protocol, and researcher were used for all groups at the same time points. |
|  | 4.3 Were outcome assessors aware of the intervention received by study participants? | | | N | The paper states: “Blinding was done so that the researchers assessing outcomes did not know which group the participants belonged to.” |
|  | 4.4 If Y/PY/NI to 4.3: Could assessment of the outcome have been influenced by knowledge of intervention received? | | | NA |  |
|  | 4.5 If Y/PY/NI to 4.4: Is it likely that assessment of the outcome was influenced by knowledge of intervention received? | | | NA |  |
|  | **Risk of bias judgement** | | | **Low** |  |
| **Bias in selection of the reported result** | 5.1 Were the data that produced this result analysed in accordance with a pre-specified analysis plan that was finalized before unblinded outcome data were available for analysis? | | | Y | There was a trial register entry, which provided details of the pre‐specified outcomes. |
|  | 5.2 ... multiple eligible outcome measurements (e.g. scales, definitions, time points) within the outcome domain? | | | N | OHIP-14 only one scale used for perceived oral health. |
|  | 5.3 ... multiple eligible analyses of the data? | | | N | Only paired t-test and ANOVA reported for OHIP-14. No evidence of multiple analysis attempts. |
|  | **Risk of bias judgement** | | | **Low** |  |
| **Overall bias** | **Risk of bias judgement** | | | **Low** |  |

Abbreviations: Y = yes, PY = probably yes, PN = probably no, N = no, NI = no information, NA = not applicable

| **Outcome** | Anterior tongue strength | **Results** | SMD, 0.26 | | **Weight** | 1 |
| --- | --- | --- | --- | --- | --- | --- |
| **Domain** | **Signalling question** | | | **Response** | | **Comments** |
| **Bias arising from the randomization process** | 1.1 Was the allocation sequence random? | | | Y | | The selected participants were allocated to non-app use, app use and control groups using a random draw. |
|  | 1.2 Was the allocation sequence concealed until participants were enrolled and assigned to interventions? | | | PY | |  |
|  | 1.3 Did baseline differences between intervention groups suggest a problem with the randomization process? | | | N | | Table 1 shows no significant differences between groups. |
|  | **Risk of bias judgement** | | | **Low** | |  |
| **Bias due to deviations from intended interventions** | 2.1.Were participants aware of their assigned intervention during the trial? | | | Y | | Due to the nature of the intervention, blinding of the participants was infeasible. Researchers directly guided the non-app group; app group used self-guided videos. Likely aware. |
|  | 2.2.Were carers and people delivering the interventions aware of participants' assigned intervention during the trial? | | | Y | |  |
|  | 2.3. If Y/PY/NI to 2.1 or 2.2: Were there deviations from the intended intervention that arose because of the experimental context? | | | PN | | There were no deviations from the intended intervention that arose. |
|  | 2.4 If Y/PY to 2.3: Were these deviations likely to have affected the outcome? | | | NA | |  |
|  | 2.5. If Y/PY/NI to 2.4: Were these deviations from intended intervention balanced between groups? | | | NA | |  |
|  | 2.6 Was an appropriate analysis used to estimate the effect of assignment to intervention? | | | PY | | Analysis appears to follow intention-to-treat principles, as all randomized participants who completed the study were included in final analysis. |
|  | 2.7 If N/PN/NI to 2.6: Was there potential for a substantial impact (on the result) of the failure to analyse participants in the group to which they were randomized? | | | NA | |  |
|  | **Risk of bias judgement** | | | **Low** | |  |
| **Bias due to missing outcome data** | 3.1 Were data for this outcome available for all, or nearly all, participants randomized? | | | PN | | 90 recruited, 76 analyzed (84.4% retention). |
|  | 3.2 If N/PN/NI to 3.1: Is there evidence that result was not biased by missing outcome data? | | | PN | | No sensitivity analysis or imputation described. |
|  | 3.3 If N/PN to 3.2: Could missingness in the outcome depend on its true value? | | | PN | | The number of lost-to-follow-up cases was similar across groups, and the reasons were also similar. |
|  | 3.4 If Y/PY/NI to 3.3: Is it likely that missingness in the outcome depended on its true value? | | | NA | |  |
|  | **Risk of bias judgement** | | | **Low** | |  |
| **Bias in measurement of the outcome** | 4.1 Was the method of measuring the outcome inappropriate? | | | N | | Anterior tongue strength was measured using the Iowa Oral Performance Instrument (IOPI), a validated device for oral muscle strength. |
|  | 4.2 Could measurement or ascertainment of the outcome have differed between intervention groups? | | | N | | The same instrument, protocol, and researcher were used for all groups at the same time points. |
|  | 4.3 Were outcome assessors aware of the intervention received by study participants? | | | N | | The paper states: “Blinding was done so that the researchers assessing outcomes did not know which group the participants belonged to.” |
|  | 4.4 If Y/PY/NI to 4.3: Could assessment of the outcome have been influenced by knowledge of intervention received? | | | NA | |  |
|  | 4.5 If Y/PY/NI to 4.4: Is it likely that assessment of the outcome was influenced by knowledge of intervention received? | | | NA | |  |
|  | **Risk of bias judgement** | | | **Low** | |  |
| **Bias in selection of the reported result** | 5.1 Were the data that produced this result analysed in accordance with a pre-specified analysis plan that was finalized before unblinded outcome data were available for analysis? | | | Y | | There was a trial register entry, which provided details of the pre‐specified outcomes. |
|  | 5.2 ... multiple eligible outcome measurements (e.g. scales, definitions, time points) within the outcome domain? | | | N | | OHIP-14 only one scale used for perceived oral health. |
|  | 5.3 ... multiple eligible analyses of the data? | | | N | | Only paired t-test and ANOVA reported for OHIP-14. No evidence of multiple analysis attempts. |
|  | **Risk of bias judgement** | | | **Low** | |  |
| **Overall bias** | **Risk of bias judgement** | | | **Low** | |  |

Abbreviations: Y = yes, PY = probably yes, PN = probably no, N = no, NI = no information, NA = not applicable

| **Outcome** | Cheek strength | **Results** | SMD, -0.33 | | **Weight** | 1 |
| --- | --- | --- | --- | --- | --- | --- |
| **Domain** | **Signalling question** | | | **Response** | | **Comments** |
| **Bias arising from the randomization process** | 1.1 Was the allocation sequence random? | | | Y | | The selected participants were allocated to non-app use, app use and control groups using a random draw. |
|  | 1.2 Was the allocation sequence concealed until participants were enrolled and assigned to interventions? | | | PY | |  |
|  | 1.3 Did baseline differences between intervention groups suggest a problem with the randomization process? | | | N | | Table 1 shows no significant differences between groups. |
|  | **Risk of bias judgement** | | | **Low** | |  |
| **Bias due to deviations from intended interventions** | 2.1.Were participants aware of their assigned intervention during the trial? | | | Y | | Due to the nature of the intervention, blinding of the participants was infeasible. Researchers directly guided the non-app group; app group used self-guided videos. Likely aware. |
|  | 2.2.Were carers and people delivering the interventions aware of participants' assigned intervention during the trial? | | | Y | |  |
|  | 2.3. If Y/PY/NI to 2.1 or 2.2: Were there deviations from the intended intervention that arose because of the experimental context? | | | PN | | There were no deviations from the intended intervention that arose. |
|  | 2.4 If Y/PY to 2.3: Were these deviations likely to have affected the outcome? | | | NA | |  |
|  | 2.5. If Y/PY/NI to 2.4: Were these deviations from intended intervention balanced between groups? | | | NA | |  |
|  | 2.6 Was an appropriate analysis used to estimate the effect of assignment to intervention? | | | PY | | Analysis appears to follow intention-to-treat principles, as all randomized participants who completed the study were included in final analysis. |
|  | 2.7 If N/PN/NI to 2.6: Was there potential for a substantial impact (on the result) of the failure to analyse participants in the group to which they were randomized? | | | NA | |  |
|  | **Risk of bias judgement** | | | **Low** | |  |
| **Bias due to missing outcome data** | 3.1 Were data for this outcome available for all, or nearly all, participants randomized? | | | PN | | 90 recruited, 76 analyzed (84.4% retention). |
|  | 3.2 If N/PN/NI to 3.1: Is there evidence that result was not biased by missing outcome data? | | | PN | | No sensitivity analysis or imputation described. |
|  | 3.3 If N/PN to 3.2: Could missingness in the outcome depend on its true value? | | | PN | | The number of lost-to-follow-up cases was similar across groups, and the reasons were also similar. |
|  | 3.4 If Y/PY/NI to 3.3: Is it likely that missingness in the outcome depended on its true value? | | | NA | |  |
|  | **Risk of bias judgement** | | | **Low** | |  |
| **Bias in measurement of the outcome** | 4.1 Was the method of measuring the outcome inappropriate? | | | N | | Cheek strength was measured using the Iowa Oral Performance Instrument (IOPI), a validated device for oral muscle strength. |
|  | 4.2 Could measurement or ascertainment of the outcome have differed between intervention groups? | | | N | | The same instrument, protocol, and researcher were used for all groups at the same time points. |
|  | 4.3 Were outcome assessors aware of the intervention received by study participants? | | | N | | The paper states: “Blinding was done so that the researchers assessing outcomes did not know which group the participants belonged to.” |
|  | 4.4 If Y/PY/NI to 4.3: Could assessment of the outcome have been influenced by knowledge of intervention received? | | | NA | |  |
|  | 4.5 If Y/PY/NI to 4.4: Is it likely that assessment of the outcome was influenced by knowledge of intervention received? | | | NA | |  |
|  | **Risk of bias judgement** | | | **Low** | |  |
| **Bias in selection of the reported result** | 5.1 Were the data that produced this result analysed in accordance with a pre-specified analysis plan that was finalized before unblinded outcome data were available for analysis? | | | Y | | There was a trial register entry, which provided details of the pre‐specified outcomes. |
|  | 5.2 ... multiple eligible outcome measurements (e.g. scales, definitions, time points) within the outcome domain? | | | N | | OHIP-14 only one scale used for perceived oral health. |
|  | 5.3 ... multiple eligible analyses of the data? | | | N | | Only paired t-test and ANOVA reported for OHIP-14. No evidence of multiple analysis attempts. |
|  | **Risk of bias judgement** | | | **Low** | |  |
| **Overall bias** | **Risk of bias judgement** | | | **Low** | |  |

Abbreviations: Y = yes, PY = probably yes, PN = probably no, N = no, NI = no information, NA = not applicable

## Ki 2021

| **Outcome** | USFR | **Results** | SMD, 2.48 | | **Weight** | 1 |
| --- | --- | --- | --- | --- | --- | --- |
| **Domain** | **Signalling question** | | | **Response** | | **Comments** |
| **Bias arising from the randomization process** | 1.1 Was the allocation sequence random? | | | NI | | The only information about randomization methods is a statement that the   study is randomized. |
|  | 1.2 Was the allocation sequence concealed until participants were enrolled and assigned to interventions? | | | NI | |  |
|  | 1.3 Did baseline differences between intervention groups suggest a problem with the randomization process? | | | N | | Between-group homogeneity was verified for subjective oral dryness, tongue  pressure, and SWAL-QoL (p > 0.05), but the USFR was lower in the intervention group (4.02) than in the control group (5.56) (p = 0.003). |
|  | **Risk of bias judgement** | | | **Some concerns** | |  |
| **Bias due to deviations from intended interventions** | 2.1.Were participants aware of their assigned intervention during the trial? | | | Y | | Participants understand the content and purpose of the study.The intervention involved mobile app education (OHEMA) versus no intervention, making it impossible to blind participants or personnel to group assignment. |
|  | 2.2.Were carers and people delivering the interventions aware of participants' assigned intervention during the trial? | | | Y | |  |
|  | 2.3. If Y/PY/NI to 2.1 or 2.2: Were there deviations from the intended intervention that arose because of the experimental context? | | | N | | There were no deviations from the intended intervention that arose. |
|  | 2.4 If Y/PY to 2.3: Were these deviations likely to have affected the outcome? | | | NA | |  |
|  | 2.5. If Y/PY/NI to 2.4: Were these deviations from intended intervention balanced between groups? | | | NA | |  |
|  | 2.6 Was an appropriate analysis used to estimate the effect of assignment to intervention? | | | Y | | ITT analysis. |
|  | 2.7 If N/PN/NI to 2.6: Was there potential for a substantial impact (on the result) of the failure to analyse participants in the group to which they were randomized? | | | NA | |  |
|  | **Risk of bias judgement** | | | **Low** | |  |
| **Bias due to missing outcome data** | 3.1 Were data for this outcome available for all, or nearly all, participants randomized? | | | N | | 46 participants (intervention group n = 24, control group n = 22) were recruited. The final analysis involved 40 individuals (intervention group n = 20, control group n = 20). The dropout rate is 13%. |
|  | 3.2 If N/PN/NI to 3.1: Is there evidence that result was not biased by missing outcome data? | | | PN | | No sensitivity analysis or imputation described. |
|  | 3.3 If N/PN to 3.2: Could missingness in the outcome depend on its true value? | | | PY | | The intervention group experienced loss to follow-up due to health issues, whereas none occurred in the control group. |
|  | 3.4 If Y/PY/NI to 3.3: Is it likely that missingness in the outcome depended on its true value? | | | PY | |  |
|  | **Risk of bias judgement** | | | **High** | |  |
| **Bias in measurement of the outcome** | 4.1 Was the method of measuring the outcome inappropriate? | | | N | | USFR measured with Schirmer strip — a standard method. |
|  | 4.2 Could measurement or ascertainment of the outcome have differed between intervention groups? | | | N | | Same method used for both groups. |
|  | 4.3 Were outcome assessors aware of the intervention received by study participants? | | | PY | | No mention of blinding of assessors. Likely aware due to study design. |
|  | 4.4 If Y/PY/NI to 4.3: Could assessment of the outcome have been influenced by knowledge of intervention received? | | | N | | USFR is objective (mm wetness). Unlikely influenced by knowledge. |
|  | 4.5 If Y/PY/NI to 4.4: Is it likely that assessment of the outcome was influenced by knowledge of intervention received? | | | NA | |  |
|  | **Risk of bias judgement** | | | **Low** | |  |
| **Bias in selection of the reported result** | 5.1 Were the data that produced this result analysed in accordance with a pre-specified analysis plan that was finalized before unblinded outcome data were available for analysis? | | | Y | | There was a trial register entry, which provided details of the pre‐specified outcomes. |
|  | 5.2 ... multiple eligible outcome measurements (e.g. scales, definitions, time points) within the outcome domain? | | | N | | USFR is one of the pre-set primary outcome indicators |
|  | 5.3 ... multiple eligible analyses of the data? | | | N | | Analysis using ANCOVA (baseline values as covariate) is reported. |
|  | **Risk of bias judgement** | | | **Low** | |  |
| **Overall bias** | **Risk of bias judgement** | | | **High** | |  |

Abbreviations: Y = yes, PY = probably yes, PN = probably no, N = no, NI = no information, NA = not applicable

## Lee 2021

| **Outcome** | GI | **Results** | SMD, 0.00 | **Weight** | 1 |
| --- | --- | --- | --- | --- | --- |
| **Domain** | **Signalling question** | | | **Response** | **Comments** |
| **Bias arising from the randomization process** | 1.1 Was the allocation sequence random? | | | NI | The only information about randomization methods is a statement that the   study is randomized. |
|  | 1.2 Was the allocation sequence concealed until participants were enrolled and assigned to interventions? | | | NI |  |
|  | 1.3 Did baseline differences between intervention groups suggest a problem with the randomization process? | | | N | Tables 1–3 show no statistically significant differences in baseline characteristics, including oral health indices. |
|  | **Risk of bias judgement** | | | **Some concerns** |  |
| **Bias due to deviations from intended interventions** | 2.1.Were participants aware of their assigned intervention during the trial? | | | Y | The interventions (lecture-based vs. app-based education) are fundamentally different in delivery method, making blinding of participants and personnel impossible. |
|  | 2.2.Were carers and people delivering the interventions aware of participants' assigned intervention during the trial? | | | Y |  |
|  | 2.3. If Y/PY/NI to 2.1 or 2.2: Were there deviations from the intended intervention that arose because of the experimental context? | | | PN | There were no deviations from the intended intervention that arose. |
|  | 2.4 If Y/PY to 2.3: Were these deviations likely to have affected the outcome? | | | NA |  |
|  | 2.5. If Y/PY/NI to 2.4: Were these deviations from intended intervention balanced between groups? | | | NA |  |
|  | 2.6 Was an appropriate analysis used to estimate the effect of assignment to intervention? | | | PY | ITT analysis. |
|  | 2.7 If N/PN/NI to 2.6: Was there potential for a substantial impact (on the result) of the failure to analyse participants in the group to which they were randomized? | | | NA |  |
|  | **Risk of bias judgement** | | | **Low** |  |
| **Bias due to missing outcome data** | 3.1 Were data for this outcome available for all, or nearly all, participants randomized? | | | N | The study included 90 participants, with 30 in each group. After excluding participants who withdrew from the study or missed the baseline or post-intervention survey, data from a total of 73 participants (25 in the non-app use group, 22 in the app use group and 26 in the control group) were included in the analysis. The dropout rate is 19%. |
|  | 3.2 If N/PN/NI to 3.1: Is there evidence that result was not biased by missing outcome data? | | | PN | No sensitivity analyses or methods to correct bias were mentioned. |
|  | 3.3 If N/PN to 3.2: Could missingness in the outcome depend on its true value? | | | PN | The reasons for loss to follow-up across groups were similar, primarily due to participants no longer being willing to participate. |
|  | 3.4 If Y/PY/NI to 3.3: Is it likely that missingness in the outcome depended on its true value? | | | NA |  |
|  | **Risk of bias judgement** | | | **Low** |  |
| **Bias in measurement of the outcome** | 4.1 Was the method of measuring the outcome inappropriate? | | | N | Löe & Silness Index is a validated, widely used measure for gingival inflammation. |
|  | 4.2 Could measurement or ascertainment of the outcome have differed between intervention groups? | | | N | Same examiner, same method, same time points. |
|  | 4.3 Were outcome assessors aware of the intervention received by study participants? | | | Y | The outcome assessors were likely aware of group assignment, and the measurement involves clinical judgment. |
|  | 4.4 If Y/PY/NI to 4.3: Could assessment of the outcome have been influenced by knowledge of intervention received? | | | PY | GI assessment involves some subjectivity. |
|  | 4.5 If Y/PY/NI to 4.4: Is it likely that assessment of the outcome was influenced by knowledge of intervention received? | | | PY |  |
|  | **Risk of bias judgement** | | | **High** |  |
| **Bias in selection of the reported result** | 5.1 Were the data that produced this result analysed in accordance with a pre-specified analysis plan that was finalized before unblinded outcome data were available for analysis? | | | Y | There was a trial register entry, which provided details of the pre‐specified outcomes. |
|  | 5.2 ... multiple eligible outcome measurements (e.g. scales, definitions, time points) within the outcome domain? | | | N | Löe & Silness Index is one of the pre-set primary outcome indicators |
|  | 5.3 ... multiple eligible analyses of the data? | | | N | The collected data were analysed using IBM SPSS Statistics (version 22.0, IBM Corporation, New York, NY, USA), with statistical significance set at .05. All continuous variables were confirmed to be normally distributed using the Kolmogorov–Smirnov test. Differences in general characteristics and oral health-related characteristics among the groups were analysed using cross-tabulations and the chisquare test and Fisher's exact test. Mean MMSE-K and ADL scores were compared between the three groups using one-way analysis of variance (ANOVA). Changes in post-intervention outcome measures were analysed using a paired samples t-test. Outcomes were analysed using repeated measures of ANOVA with a within subjects factor (time) and a between subjects factor (group). No violations in sphericity were found in Mauchly's sphericity test. |
|  | **Risk of bias judgement** | | | **Low** |  |
| **Overall bias** | **Risk of bias judgement** | | | **High** |  |

Abbreviations: Y = yes, PY = probably yes, PN = probably no, N = no, NI = no information, NA = not applicable

| **Outcome** | OHIP-14 | **Results** | SMD, -0.14 | **Weight** | | 1 |
| --- | --- | --- | --- | --- | --- | --- |
| **Domain** | **Signalling question** | | | **Response** | | **Comments** |
| **Bias arising from the randomization process** | 1.1 Was the allocation sequence random? | | | NI | | The only information about randomization methods is a statement that the   study is randomized. |
|  | 1.2 Was the allocation sequence concealed until participants were enrolled and assigned to interventions? | | | NI | |  |
|  | 1.3 Did baseline differences between intervention groups suggest a problem with the randomization process? | | | N | | Tables 1–3 show no statistically significant differences in baseline characteristics, including oral health indices. |
|  | **Risk of bias judgement** | | | **Some concerns** | |  |
| **Bias due to deviations from intended interventions** | 2.1.Were participants aware of their assigned intervention during the trial? | | | Y | | The interventions (lecture-based vs. app-based education) are fundamentally different in delivery method, making blinding of participants and personnel impossible. |
|  | 2.2.Were carers and people delivering the interventions aware of participants' assigned intervention during the trial? | | | Y | |  |
|  | 2.3. If Y/PY/NI to 2.1 or 2.2: Were there deviations from the intended intervention that arose because of the experimental context? | | | PN | | There were no deviations from the intended intervention that arose. |
|  | 2.4 If Y/PY to 2.3: Were these deviations likely to have affected the outcome? | | | NA | |  |
|  | 2.5. If Y/PY/NI to 2.4: Were these deviations from intended intervention balanced between groups? | | | NA | |  |
|  | 2.6 Was an appropriate analysis used to estimate the effect of assignment to intervention? | | | PY | | ITT analysis. |
|  | 2.7 If N/PN/NI to 2.6: Was there potential for a substantial impact (on the result) of the failure to analyse participants in the group to which they were randomized? | | | NA | |  |
|  | **Risk of bias judgement** | | | **Low** | |  |
| **Bias due to missing outcome data** | 3.1 Were data for this outcome available for all, or nearly all, participants randomized? | | | N | | The study included 90 participants, with 30 in each group. After excluding participants who withdrew from the study or missed the baseline or post-intervention survey, data from a total of 73 participants (25 in the non-app use group, 22 in the app use group and 26 in the control group) were included in the analysis. The dropout rate is 19%. |
|  | 3.2 If N/PN/NI to 3.1: Is there evidence that result was not biased by missing outcome data? | | | PN | | No sensitivity analyses or methods to correct bias were mentioned. |
|  | 3.3 If N/PN to 3.2: Could missingness in the outcome depend on its true value? | | | PN | | The reasons for loss to follow-up across groups were similar, primarily due to participants no longer being willing to participate. |
|  | 3.4 If Y/PY/NI to 3.3: Is it likely that missingness in the outcome depended on its true value? | | | NA | |  |
|  | **Risk of bias judgement** | | | **Low** | |  |
| **Bias in measurement of the outcome** | 4.1 Was the method of measuring the outcome inappropriate? | | | N | | OHIP-14 is a validated scale. |
|  | 4.2 Could measurement or ascertainment of the outcome have differed between intervention groups? | | | N | | All groups completed the same questionnaire at same time points. |
|  | 4.3 Were outcome assessors aware of the intervention received by study participants? | | | Y | | OHIP-14 is self-reported; participants were aware of their group. |
|  | 4.4 If Y/PY/NI to 4.3: Could assessment of the outcome have been influenced by knowledge of intervention received? | | | PY | | OHIP-14 can be influenced by awareness of intervention. |
|  | 4.5 If Y/PY/NI to 4.4: Is it likely that assessment of the outcome was influenced by knowledge of intervention received? | | | PY | |  |
|  | **Risk of bias judgement** | | | **High** | |  |
| **Bias in selection of the reported result** | 5.1 Were the data that produced this result analysed in accordance with a pre-specified analysis plan that was finalized before unblinded outcome data were available for analysis? | | | Y | | There was a trial register entry, which provided details of the pre‐specified outcomes. |
|  | 5.2 ... multiple eligible outcome measurements (e.g. scales, definitions, time points) within the outcome domain? | | | N | | OHIP-14 is one of the pre-set primary outcome indicators |
|  | 5.3 ... multiple eligible analyses of the data? | | | N | | The collected data were analysed using IBM SPSS Statistics (version 22.0, IBM Corporation, New York, NY, USA), with statistical significance set at .05. All continuous variables were confirmed to be normally distributed using the Kolmogorov–Smirnov test. Differences in general characteristics and oral health-related characteristics among the groups were analysed using cross-tabulations and the chisquare test and Fisher's exact test. Mean MMSE-K and ADL scores were compared between the three groups using one-way analysis of variance (ANOVA). Changes in post-intervention outcome measures were analysed using a paired samples t-test. Outcomes were analysed using repeated measures of ANOVA with a within subjects factor (time) and a between subjects factor (group). No violations in sphericity were found in Mauchly's sphericity test. |
|  | **Risk of bias judgement** | | | **Low** | |  |
| **Overall bias** | **Risk of bias judgement** | | | | **High** |  |

Abbreviations: Y = yes, PY = probably yes, PN = probably no, N = no, NI = no information, NA = not applicable

| **Outcome** | GOHAI | **Results** | SMD, 0.27 | | **Weight** | 1 |
| --- | --- | --- | --- | --- | --- | --- |
| **Domain** | **Signalling question** | | | **Response** | | **Comments** |
| **Bias arising from the randomization process** | 1.1 Was the allocation sequence random? | | | NI | | The only information about randomization methods is a statement that the   study is randomized. |
|  | 1.2 Was the allocation sequence concealed until participants were enrolled and assigned to interventions? | | | NI | |  |
|  | 1.3 Did baseline differences between intervention groups suggest a problem with the randomization process? | | | N | | Tables 1–3 show no statistically significant differences in baseline characteristics, including oral health indices. |
|  | **Risk of bias judgement** | | | **Some concerns** | |  |
| **Bias due to deviations from intended interventions** | 2.1.Were participants aware of their assigned intervention during the trial? | | | Y | | The interventions (lecture-based vs. app-based education) are fundamentally different in delivery method, making blinding of participants and personnel impossible. |
|  | 2.2.Were carers and people delivering the interventions aware of participants' assigned intervention during the trial? | | | Y | |  |
|  | 2.3. If Y/PY/NI to 2.1 or 2.2: Were there deviations from the intended intervention that arose because of the experimental context? | | | PN | | There were no deviations from the intended intervention that arose. |
|  | 2.4 If Y/PY to 2.3: Were these deviations likely to have affected the outcome? | | | NA | |  |
|  | 2.5. If Y/PY/NI to 2.4: Were these deviations from intended intervention balanced between groups? | | | NA | |  |
|  | 2.6 Was an appropriate analysis used to estimate the effect of assignment to intervention? | | | PY | | ITT analysis. |
|  | 2.7 If N/PN/NI to 2.6: Was there potential for a substantial impact (on the result) of the failure to analyse participants in the group to which they were randomized? | | | NA | |  |
|  | **Risk of bias judgement** | | | **Low** | |  |
| **Bias due to missing outcome data** | 3.1 Were data for this outcome available for all, or nearly all, participants randomized? | | | N | | The study included 90 participants, with 30 in each group. After excluding participants who withdrew from the study or missed the baseline or post-intervention survey, data from a total of 73 participants (25 in the non-app use group, 22 in the app use group and 26 in the control group) were included in the analysis. The dropout rate is 19%. |
|  | 3.2 If N/PN/NI to 3.1: Is there evidence that result was not biased by missing outcome data? | | | PN | | No sensitivity analyses or methods to correct bias were mentioned. |
|  | 3.3 If N/PN to 3.2: Could missingness in the outcome depend on its true value? | | | PN | | The reasons for loss to follow-up across groups were similar, primarily due to participants no longer being willing to participate. |
|  | 3.4 If Y/PY/NI to 3.3: Is it likely that missingness in the outcome depended on its true value? | | | NA | |  |
|  | **Risk of bias judgement** | | | **Low** | |  |
| **Bias in measurement of the outcome** | 4.1 Was the method of measuring the outcome inappropriate? | | | N | | GOHAI is a validated instrument for older adults’ oral health-related quality of life. |
|  | 4.2 Could measurement or ascertainment of the outcome have differed between intervention groups? | | | N | | Same questionnaire used for all groups at same time points. |
|  | 4.3 Were outcome assessors aware of the intervention received by study participants? | | | Y | | GOHAI is self-reported; participants were aware of their group. |
|  | 4.4 If Y/PY/NI to 4.3: Could assessment of the outcome have been influenced by knowledge of intervention received? | | | PY | | GOHAI can be influenced by awareness of intervention. |
|  | 4.5 If Y/PY/NI to 4.4: Is it likely that assessment of the outcome was influenced by knowledge of intervention received? | | | PY | |  |
|  | **Risk of bias judgement** | | | **High** | |  |
| **Bias in selection of the reported result** | 5.1 Were the data that produced this result analysed in accordance with a pre-specified analysis plan that was finalized before unblinded outcome data were available for analysis? | | | Y | | There was a trial register entry, which provided details of the pre‐specified outcomes. |
|  | 5.2 ... multiple eligible outcome measurements (e.g. scales, definitions, time points) within the outcome domain? | | | N | | GOHAI is one of the pre-set primary outcome indicators. |
|  | 5.3 ... multiple eligible analyses of the data? | | | N | | The collected data were analysed using IBM SPSS Statistics (version 22.0, IBM Corporation, New York, NY, USA), with statistical significance set at .05. All continuous variables were confirmed to be normally distributed using the Kolmogorov–Smirnov test. Differences in general characteristics and oral health-related characteristics among the groups were analysed using cross-tabulations and the chisquare test and Fisher's exact test. Mean MMSE-K and ADL scores were compared between the three groups using one-way analysis of variance (ANOVA). Changes in post-intervention outcome measures were analysed using a paired samples t-test. Outcomes were analysed using repeated measures of ANOVA with a within subjects factor (time) and a between subjects factor (group). No violations in sphericity were found in Mauchly's sphericity test. |
|  | **Risk of bias judgement** | | | **Low** | |  |
| **Overall bias** | **Risk of bias judgement** | | | **High** | |  |

Abbreviations: Y = yes, PY = probably yes, PN = probably no, N = no, NI = no information, NA = not applicable

| **Outcome** | O'Leary index | **Results** | SMD, -0.43 | **Weight** | 1 |
| --- | --- | --- | --- | --- | --- |
| **Domain** | **Signalling question** | | | **Response** | **Comments** |
| **Bias arising from the randomization process** | 1.1 Was the allocation sequence random? | | | NI | The only information about randomization methods is a statement that the   study is randomized. |
|  | 1.2 Was the allocation sequence concealed until participants were enrolled and assigned to interventions? | | | NI |  |
|  | 1.3 Did baseline differences between intervention groups suggest a problem with the randomization process? | | | N | Tables 1–3 show no statistically significant differences in baseline characteristics, including oral health indices. |
|  | **Risk of bias judgement** | | | **Some concerns** |  |
| **Bias due to deviations from intended interventions** | 2.1.Were participants aware of their assigned intervention during the trial? | | | Y | The interventions (lecture-based vs. app-based education) are fundamentally different in delivery method, making blinding of participants and personnel impossible. |
|  | 2.2.Were carers and people delivering the interventions aware of participants' assigned intervention during the trial? | | | Y |  |
|  | 2.3. If Y/PY/NI to 2.1 or 2.2: Were there deviations from the intended intervention that arose because of the experimental context? | | | PN | There were no deviations from the intended intervention that arose. |
|  | 2.4 If Y/PY to 2.3: Were these deviations likely to have affected the outcome? | | | NA |  |
|  | 2.5. If Y/PY/NI to 2.4: Were these deviations from intended intervention balanced between groups? | | | NA |  |
|  | 2.6 Was an appropriate analysis used to estimate the effect of assignment to intervention? | | | PY | ITT analysis. |
|  | 2.7 If N/PN/NI to 2.6: Was there potential for a substantial impact (on the result) of the failure to analyse participants in the group to which they were randomized? | | | NA |  |
|  | **Risk of bias judgement** | | | **Low** |  |
| **Bias due to missing outcome data** | 3.1 Were data for this outcome available for all, or nearly all, participants randomized? | | | N | The study included 90 participants, with 30 in each group. After excluding participants who withdrew from the study or missed the baseline or post-intervention survey, data from a total of 73 participants (25 in the non-app use group, 22 in the app use group and 26 in the control group) were included in the analysis. The dropout rate is 19%. |
|  | 3.2 If N/PN/NI to 3.1: Is there evidence that result was not biased by missing outcome data? | | | PN | No sensitivity analyses or methods to correct bias were mentioned. |
|  | 3.3 If N/PN to 3.2: Could missingness in the outcome depend on its true value? | | | PN | The reasons for loss to follow-up across groups were similar, primarily due to participants no longer being willing to participate. |
|  | 3.4 If Y/PY/NI to 3.3: Is it likely that missingness in the outcome depended on its true value? | | | NA |  |
|  | **Risk of bias judgement** | | | **Low** |  |
| **Bias in measurement of the outcome** | 4.1 Was the method of measuring the outcome inappropriate? | | | N | O’Leary index is a standard plaque index. |
|  | 4.2 Could measurement or ascertainment of the outcome have differed between intervention groups? | | | N | Same examiner(s) likely used same method for all groups. |
|  | 4.3 Were outcome assessors aware of the intervention received by study participants? | | | Y | The outcome assessors were likely aware of group assignment. |
|  | 4.4 If Y/PY/NI to 4.3: Could assessment of the outcome have been influenced by knowledge of intervention received? | | | PN | Plaque index is objective; less prone to assessor bias. |
|  | 4.5 If Y/PY/NI to 4.4: Is it likely that assessment of the outcome was influenced by knowledge of intervention received? | | | NA |  |
|  | **Risk of bias judgement** | | | **Low** |  |
| **Bias in selection of the reported result** | 5.1 Were the data that produced this result analysed in accordance with a pre-specified analysis plan that was finalized before unblinded outcome data were available for analysis? | | | Y | There was a trial register entry, which provided details of the pre‐specified outcomes. |
|  | 5.2 ... multiple eligible outcome measurements (e.g. scales, definitions, time points) within the outcome domain? | | | N | O'Leary index is one of the pre-set primary outcome indicators |
|  | 5.3 ... multiple eligible analyses of the data? | | | N | The collected data were analysed using IBM SPSS Statistics (version 22.0, IBM Corporation, New York, NY, USA), with statistical significance set at .05. All continuous variables were confirmed to be normally distributed using the Kolmogorov–Smirnov test. Differences in general characteristics and oral health-related characteristics among the groups were analysed using cross-tabulations and the chisquare test and Fisher's exact test. Mean MMSE-K and ADL scores were compared between the three groups using one-way analysis of variance (ANOVA). Changes in post-intervention outcome measures were analysed using a paired samples t-test. Outcomes were analysed using repeated measures of ANOVA with a within subjects factor (time) and a between subjects factor (group). No violations in sphericity were found in Mauchly's sphericity test. |
|  | **Risk of bias judgement** | | | **Low** |  |
| **Overall bias** | **Risk of bias judgement** | | | **Some concerns** |  |

Abbreviations: Y = yes, PY = probably yes, PN = probably no, N = no, NI = no information, NA = not applicable

| **Outcome** | Winkel tongue coating index | **Results** | SMD, -1.36 | | **Weight** | 1 |
| --- | --- | --- | --- | --- | --- | --- |
| **Domain** | **Signalling question** | | | **Response** | | **Comments** |
| **Bias arising from the randomization process** | 1.1 Was the allocation sequence random? | | | NI | | The only information about randomization methods is a statement that the   study is randomized. |
|  | 1.2 Was the allocation sequence concealed until participants were enrolled and assigned to interventions? | | | NI | |  |
|  | 1.3 Did baseline differences between intervention groups suggest a problem with the randomization process? | | | N | | Tables 1–3 show no statistically significant differences in baseline characteristics, including oral health indices. |
|  | **Risk of bias judgement** | | | **Some concerns** | |  |
| **Bias due to deviations from intended interventions** | 2.1.Were participants aware of their assigned intervention during the trial? | | | Y | | The interventions (lecture-based vs. app-based education) are fundamentally different in delivery method, making blinding of participants and personnel impossible. |
|  | 2.2.Were carers and people delivering the interventions aware of participants' assigned intervention during the trial? | | | Y | |  |
|  | 2.3. If Y/PY/NI to 2.1 or 2.2: Were there deviations from the intended intervention that arose because of the experimental context? | | | PN | | There were no deviations from the intended intervention that arose. |
|  | 2.4 If Y/PY to 2.3: Were these deviations likely to have affected the outcome? | | | NA | |  |
|  | 2.5. If Y/PY/NI to 2.4: Were these deviations from intended intervention balanced between groups? | | | NA | |  |
|  | 2.6 Was an appropriate analysis used to estimate the effect of assignment to intervention? | | | PY | | ITT analysis. |
|  | 2.7 If N/PN/NI to 2.6: Was there potential for a substantial impact (on the result) of the failure to analyse participants in the group to which they were randomized? | | | NA | |  |
|  | **Risk of bias judgement** | | | **Low** | |  |
| **Bias due to missing outcome data** | 3.1 Were data for this outcome available for all, or nearly all, participants randomized? | | | N | | The study included 90 participants, with 30 in each group. After excluding participants who withdrew from the study or missed the baseline or post-intervention survey, data from a total of 73 participants (25 in the non-app use group, 22 in the app use group and 26 in the control group) were included in the analysis. The dropout rate is 19%. |
|  | 3.2 If N/PN/NI to 3.1: Is there evidence that result was not biased by missing outcome data? | | | PN | | No sensitivity analyses or methods to correct bias were mentioned. |
|  | 3.3 If N/PN to 3.2: Could missingness in the outcome depend on its true value? | | | PN | | The reasons for loss to follow-up across groups were similar, primarily due to participants no longer being willing to participate. |
|  | 3.4 If Y/PY/NI to 3.3: Is it likely that missingness in the outcome depended on its true value? | | | NA | |  |
|  | **Risk of bias judgement** | | | **Low** | |  |
| **Bias in measurement of the outcome** | 4.1 Was the method of measuring the outcome inappropriate? | | | N | | Winkel tongue coating index is a validated method. |
|  | 4.2 Could measurement or ascertainment of the outcome have differed between intervention groups? | | | N | | Same examiner(s) likely used same method for all groups. |
|  | 4.3 Were outcome assessors aware of the intervention received by study participants? | | | Y | | The outcome assessors were likely aware of group assignment. |
|  | 4.4 If Y/PY/NI to 4.3: Could assessment of the outcome have been influenced by knowledge of intervention received? | | | PN | | Winkel tongue coating index is objective; less prone to assessor bias. |
|  | 4.5 If Y/PY/NI to 4.4: Is it likely that assessment of the outcome was influenced by knowledge of intervention received? | | | NA | |  |
|  | **Risk of bias judgement** | | | **Low** | |  |
| **Bias in selection of the reported result** | 5.1 Were the data that produced this result analysed in accordance with a pre-specified analysis plan that was finalized before unblinded outcome data were available for analysis? | | | Y | | There was a trial register entry, which provided details of the pre‐specified outcomes. |
|  | 5.2 ... multiple eligible outcome measurements (e.g. scales, definitions, time points) within the outcome domain? | | | N | | Winkel tongue coating index is one of the pre-set primary outcome indicators. |
|  | 5.3 ... multiple eligible analyses of the data? | | | N | | The collected data were analysed using IBM SPSS Statistics (version 22.0, IBM Corporation, New York, NY, USA), with statistical significance set at .05. All continuous variables were confirmed to be normally distributed using the Kolmogorov–Smirnov test. Differences in general characteristics and oral health-related characteristics among the groups were analysed using cross-tabulations and the chisquare test and Fisher's exact test. Mean MMSE-K and ADL scores were compared between the three groups using one-way analysis of variance (ANOVA). Changes in post-intervention outcome measures were analysed using a paired samples t-test. Outcomes were analysed using repeated measures of ANOVA with a within subjects factor (time) and a between subjects factor (group). No violations in sphericity were found in Mauchly's sphericity test. |
|  | **Risk of bias judgement** | | | **Low** | |  |
| **Overall bias** | **Risk of bias judgement** | | | **Some concerns** | |  |

Abbreviations: Y = yes, PY = probably yes, PN = probably no, N = no, NI = no information, NA = not applicable

## lee 2024

| **Outcome** | USFR | **Results** | SMD, 2.45 | **Weight** | 1 |
| --- | --- | --- | --- | --- | --- |
| **Domain** | **Signalling question** | | **Response** | | **Comments** |
| **Bias arising from the randomization process** | 1.1 Was the allocation sequence random? | | NI | | The only information about randomization methods is a statement that the   study is randomized. |
|  | 1.2 Was the allocation sequence concealed until participants were enrolled and assigned to interventions? | | NI | |  |
|  | 1.3 Did baseline differences between intervention groups suggest a problem with the randomization process? | | N | | There were no statistically significant differences in oral health-related behavior characteristics and general characteristics of the participants(p > 0.05). |
|  | **Risk of bias judgement** | | **Some concerns** | |  |
| **Bias due to deviations from intended interventions** | 2.1.Were participants aware of their assigned intervention during the trial? | | Y | | The interventions are fundamentally different in delivery method, making blinding of participants and personnel impossible. |
|  | 2.2.Were carers and people delivering the interventions aware of participants' assigned intervention during the trial? | | PY | |  |
|  | 2.3. If Y/PY/NI to 2.1 or 2.2: Were there deviations from the intended intervention that arose because of the experimental context? | | PN | | There were no deviations from the intended intervention that arose. |
|  | 2.4 If Y/PY to 2.3: Were these deviations likely to have affected the outcome? | | NA | |  |
|  | 2.5. If Y/PY/NI to 2.4: Were these deviations from intended intervention balanced between groups? | | NA | |  |
|  | 2.6 Was an appropriate analysis used to estimate the effect of assignment to intervention? | | Y | | Analysis appears to follow intention-to-treat principles, as all randomized participants who completed the study were included in final analysis. |
|  | 2.7 If N/PN/NI to 2.6: Was there potential for a substantial impact (on the result) of the failure to analyse participants in the group to which they were randomized? | | NA | |  |
|  | **Risk of bias judgement** | | **Low** | |  |
| **Bias due to missing outcome data** | 3.1 Were data for this outcome available for all, or nearly all, participants randomized? | | PN | | "60 participants (30 per group)", "the final analysis included data from 28 participants in the experimental group and 23 in the control group". The dropout rate is 15%. |
|  | 3.2 If N/PN/NI to 3.1: Is there evidence that result was not biased by missing outcome data? | | PN | | No sensitivity analyses or methods to correct bias were mentioned. |
|  | 3.3 If N/PN to 3.2: Could missingness in the outcome depend on its true value? | | PN | | Participants withdrew because they were unwilling to continue. |
|  | 3.4 If Y/PY/NI to 3.3: Is it likely that missingness in the outcome depended on its true value? | | NA | |  |
|  | **Risk of bias judgement** | | **Low** | |  |
| **Bias in measurement of the outcome** | 4.1 Was the method of measuring the outcome inappropriate? | | N | | USFR measured by expectoration into a test tube is a standard, valid method. |
|  | 4.2 Could measurement or ascertainment of the outcome have differed between intervention groups? | | PN | | Same measurement procedure described for both groups, at same time points. |
|  | 4.3 Were outcome assessors aware of the intervention received by study participants? | | PY | | Not explicitly stated, but likely that assessors were aware due to unblinded study design. |
|  | 4.4 If Y/PY/NI to 4.3: Could assessment of the outcome have been influenced by knowledge of intervention received? | | PN | | USFR is an objective, quantitative measure unlikely to be influenced by assessor judgement. |
|  | 4.5 If Y/PY/NI to 4.4: Is it likely that assessment of the outcome was influenced by knowledge of intervention received? | | NA | |  |
|  | **Risk of bias judgement** | | **Low** | |  |
| **Bias in selection of the reported result** | 5.1 Were the data that produced this result analysed in accordance with a pre-specified analysis plan that was finalized before unblinded outcome data were available for analysis? | | NI | | No mention of protocol. |
|  | 5.2 ... multiple eligible outcome measurements (e.g. scales, definitions, time points) within the outcome domain? | | NI | | No mention of protocol. |
|  | 5.3 ... multiple eligible analyses of the data? | | NI | | No mention of protocol. |
|  | **Risk of bias judgement** | | **Some concerns** | |  |
| **Overall bias** | **Risk of bias judgement** | | **Some concerns** | |  |

Abbreviations: Y = yes, PY = probably yes, PN = probably no, N = no, NI = no information, NA = not applicable

| **Outcome** | Posterior tongue strength | **Results** | | SMD, 0.43 | **Weight** | 1 |
| --- | --- | --- | --- | --- | --- | --- |
| **Domain** | **Signalling question** | | **Response** | | | **Comments** |
| **Bias arising from the randomization process** | 1.1 Was the allocation sequence random? | | NI | | | The only information about randomization methods is a statement that the   study is randomized. |
|  | 1.2 Was the allocation sequence concealed until participants were enrolled and assigned to interventions? | | NI | | |  |
|  | 1.3 Did baseline differences between intervention groups suggest a problem with the randomization process? | | N | | | There were no statistically significant differences in oral health-related behavior characteristics and general characteristics of the participants(p > 0.05). |
|  | **Risk of bias judgement** | | **Some concerns** | | |  |
| **Bias due to deviations from intended interventions** | 2.1.Were participants aware of their assigned intervention during the trial? | | Y | | | The interventions are fundamentally different in delivery method, making blinding of participants and personnel impossible. |
|  | 2.2.Were carers and people delivering the interventions aware of participants' assigned intervention during the trial? | | PY | | |  |
|  | 2.3. If Y/PY/NI to 2.1 or 2.2: Were there deviations from the intended intervention that arose because of the experimental context? | | PN | | | There were no deviations from the intended intervention that arose. |
|  | 2.4 If Y/PY to 2.3: Were these deviations likely to have affected the outcome? | | NA | | |  |
|  | 2.5. If Y/PY/NI to 2.4: Were these deviations from intended intervention balanced between groups? | | NA | | |  |
|  | 2.6 Was an appropriate analysis used to estimate the effect of assignment to intervention? | | Y | | | Analysis appears to follow intention-to-treat principles, as all randomized participants who completed the study were included in final analysis. |
|  | 2.7 If N/PN/NI to 2.6: Was there potential for a substantial impact (on the result) of the failure to analyse participants in the group to which they were randomized? | | NA | | |  |
|  | **Risk of bias judgement** | | **Low** | | |  |
| **Bias due to missing outcome data** | 3.1 Were data for this outcome available for all, or nearly all, participants randomized? | | PN | | | "60 participants (30 per group)", "the final analysis included data from 28 participants in the experimental group and 23 in the control group". The dropout rate is 15%. |
|  | 3.2 If N/PN/NI to 3.1: Is there evidence that result was not biased by missing outcome data? | | PN | | | No sensitivity analyses or methods to correct bias were mentioned. |
|  | 3.3 If N/PN to 3.2: Could missingness in the outcome depend on its true value? | | PN | | | Participants withdrew because they were unwilling to continue. |
|  | 3.4 If Y/PY/NI to 3.3: Is it likely that missingness in the outcome depended on its true value? | | NA | | |  |
|  | **Risk of bias judgement** | | **Low** | | |  |
| **Bias in measurement of the outcome** | 4.1 Was the method of measuring the outcome inappropriate? | | N | | | Iowa Oral Performance Instrument (IOPI) is valid for tongue strength. |
|  | 4.2 Could measurement or ascertainment of the outcome have differed between intervention groups? | | PN | | | Same instrument used for both groups, same timing. |
|  | 4.3 Were outcome assessors aware of the intervention received by study participants? | | PY | | | Not explicitly stated, but likely that assessors were aware due to unblinded study design. |
|  | 4.4 If Y/PY/NI to 4.3: Could assessment of the outcome have been influenced by knowledge of intervention received? | | PN | | | IOPI is an objective, quantitative measure unlikely to be influenced by assessor judgement. |
|  | 4.5 If Y/PY/NI to 4.4: Is it likely that assessment of the outcome was influenced by knowledge of intervention received? | | NA | | |  |
|  | **Risk of bias judgement** | | **Low** | | |  |
| **Bias in selection of the reported result** | 5.1 Were the data that produced this result analysed in accordance with a pre-specified analysis plan that was finalized before unblinded outcome data were available for analysis? | | NI | | | No mention of protocol. |
|  | 5.2 ... multiple eligible outcome measurements (e.g. scales, definitions, time points) within the outcome domain? | | NI | | | No mention of protocol. |
|  | 5.3 ... multiple eligible analyses of the data? | | NI | | | No mention of protocol. |
|  | **Risk of bias judgement** | | **Some concerns** | | |  |
| **Overall bias** | **Risk of bias judgement** | | **Some concerns** | | |  |

Abbreviations: Y = yes, PY = probably yes, PN = probably no, N = no, NI = no information, NA = not applicable

| **Outcome** | OHIP-14 | **Results** | | SMD, -0.20 | **Weight** | 1 |
| --- | --- | --- | --- | --- | --- | --- |
| **Domain** | **Signalling question** | | **Response** | | | **Comments** |
| **Bias arising from the randomization process** | 1.1 Was the allocation sequence random? | | NI | | | The only information about randomization methods is a statement that the   study is randomized. |
|  | 1.2 Was the allocation sequence concealed until participants were enrolled and assigned to interventions? | | NI | | |  |
|  | 1.3 Did baseline differences between intervention groups suggest a problem with the randomization process? | | N | | | There were no statistically significant differences in oral health-related behavior characteristics and general characteristics of the participants(p > 0.05). |
|  | **Risk of bias judgement** | | **Some concerns** | | |  |
| **Bias due to deviations from intended interventions** | 2.1.Were participants aware of their assigned intervention during the trial? | | Y | | | The interventions are fundamentally different in delivery method, making blinding of participants and personnel impossible. |
|  | 2.2.Were carers and people delivering the interventions aware of participants' assigned intervention during the trial? | | PY | | |  |
|  | 2.3. If Y/PY/NI to 2.1 or 2.2: Were there deviations from the intended intervention that arose because of the experimental context? | | PN | | | There were no deviations from the intended intervention that arose. |
|  | 2.4 If Y/PY to 2.3: Were these deviations likely to have affected the outcome? | | NA | | |  |
|  | 2.5. If Y/PY/NI to 2.4: Were these deviations from intended intervention balanced between groups? | | NA | | |  |
|  | 2.6 Was an appropriate analysis used to estimate the effect of assignment to intervention? | | Y | | | Analysis appears to follow intention-to-treat principles, as all randomized participants who completed the study were included in final analysis. |
|  | 2.7 If N/PN/NI to 2.6: Was there potential for a substantial impact (on the result) of the failure to analyse participants in the group to which they were randomized? | | NA | | |  |
|  | **Risk of bias judgement** | | **Low** | | |  |
| **Bias due to missing outcome data** | 3.1 Were data for this outcome available for all, or nearly all, participants randomized? | | PN | | | "60 participants (30 per group)", "the final analysis included data from 28 participants in the experimental group and 23 in the control group". The dropout rate is 15%. |
|  | 3.2 If N/PN/NI to 3.1: Is there evidence that result was not biased by missing outcome data? | | PN | | | No sensitivity analyses or methods to correct bias were mentioned. |
|  | 3.3 If N/PN to 3.2: Could missingness in the outcome depend on its true value? | | PN | | | Participants withdrew because they were unwilling to continue. |
|  | 3.4 If Y/PY/NI to 3.3: Is it likely that missingness in the outcome depended on its true value? | | NA | | |  |
|  | **Risk of bias judgement** | | **Low** | | |  |
| **Bias in measurement of the outcome** | 4.1 Was the method of measuring the outcome inappropriate? | | N | | | OHIP-14 is a validated instrument for oral health-related quality of life. |
|  | 4.2 Could measurement or ascertainment of the outcome have differed between intervention groups? | | PN | | | Same instrument and time points used for both groups. |
|  | 4.3 Were outcome assessors aware of the intervention received by study participants? | | PY | | | OHIP-14 is self-reported; participants were aware of their group. |
|  | 4.4 If Y/PY/NI to 4.3: Could assessment of the outcome have been influenced by knowledge of intervention received? | | PY | | | OHIP-14 can be influenced by awareness of intervention. |
|  | 4.5 If Y/PY/NI to 4.4: Is it likely that assessment of the outcome was influenced by knowledge of intervention received? | | PY | | |  |
|  | **Risk of bias judgement** | | **High** | | |  |
| **Bias in selection of the reported result** | 5.1 Were the data that produced this result analysed in accordance with a pre-specified analysis plan that was finalized before unblinded outcome data were available for analysis? | | NI | | | No mention of protocol. |
|  | 5.2 ... multiple eligible outcome measurements (e.g. scales, definitions, time points) within the outcome domain? | | NI | | | No mention of protocol. |
|  | 5.3 ... multiple eligible analyses of the data? | | NI | | | No mention of protocol. |
|  | **Risk of bias judgement** | | **Some concerns** | | |  |
| **Overall bias** | **Risk of bias judgement** | | **High** | | |  |

Abbreviations: Y = yes, PY = probably yes, PN = probably no, N = no, NI = no information, NA = not applicable

| **Outcome** | GOHAI | **Results** | | SMD, 0.16 | **Weight** | 1 |
| --- | --- | --- | --- | --- | --- | --- |
| **Domain** | **Signalling question** | | **Response** | | | **Comments** |
| **Bias arising from the randomization process** | 1.1 Was the allocation sequence random? | | NI | | | The only information about randomization methods is a statement that the   study is randomized. |
|  | 1.2 Was the allocation sequence concealed until participants were enrolled and assigned to interventions? | | NI | | |  |
|  | 1.3 Did baseline differences between intervention groups suggest a problem with the randomization process? | | N | | | There were no statistically significant differences in oral health-related behavior characteristics and general characteristics of the participants(p > 0.05). |
|  | **Risk of bias judgement** | | **Some concerns** | | |  |
| **Bias due to deviations from intended interventions** | 2.1.Were participants aware of their assigned intervention during the trial? | | Y | | | The interventions are fundamentally different in delivery method, making blinding of participants and personnel impossible. |
|  | 2.2.Were carers and people delivering the interventions aware of participants' assigned intervention during the trial? | | PY | | |  |
|  | 2.3. If Y/PY/NI to 2.1 or 2.2: Were there deviations from the intended intervention that arose because of the experimental context? | | PN | | | There were no deviations from the intended intervention that arose. |
|  | 2.4 If Y/PY to 2.3: Were these deviations likely to have affected the outcome? | | NA | | |  |
|  | 2.5. If Y/PY/NI to 2.4: Were these deviations from intended intervention balanced between groups? | | NA | | |  |
|  | 2.6 Was an appropriate analysis used to estimate the effect of assignment to intervention? | | Y | | | Analysis appears to follow intention-to-treat principles, as all randomized participants who completed the study were included in final analysis. |
|  | 2.7 If N/PN/NI to 2.6: Was there potential for a substantial impact (on the result) of the failure to analyse participants in the group to which they were randomized? | | NA | | |  |
|  | **Risk of bias judgement** | | **Low** | | |  |
| **Bias due to missing outcome data** | 3.1 Were data for this outcome available for all, or nearly all, participants randomized? | | PN | | | "60 participants (30 per group)", "the final analysis included data from 28 participants in the experimental group and 23 in the control group". The dropout rate is 15%. |
|  | 3.2 If N/PN/NI to 3.1: Is there evidence that result was not biased by missing outcome data? | | PN | | | No sensitivity analyses or methods to correct bias were mentioned. |
|  | 3.3 If N/PN to 3.2: Could missingness in the outcome depend on its true value? | | PN | | | Participants withdrew because they were unwilling to continue. |
|  | 3.4 If Y/PY/NI to 3.3: Is it likely that missingness in the outcome depended on its true value? | | NA | | |  |
|  | **Risk of bias judgement** | | **Low** | | |  |
| **Bias in measurement of the outcome** | 4.1 Was the method of measuring the outcome inappropriate? | | N | | | GOHAI is a validated instrument for older adults’ oral health-related quality of life. |
|  | 4.2 Could measurement or ascertainment of the outcome have differed between intervention groups? | | PN | | | Same instrument and time points used for both groups. |
|  | 4.3 Were outcome assessors aware of the intervention received by study participants? | | PY | | | GOHAI is self-reported; participants were aware of their group. |
|  | 4.4 If Y/PY/NI to 4.3: Could assessment of the outcome have been influenced by knowledge of intervention received? | | PY | | | GOHAI can be influenced by awareness of intervention. |
|  | 4.5 If Y/PY/NI to 4.4: Is it likely that assessment of the outcome was influenced by knowledge of intervention received? | | PY | | |  |
|  | **Risk of bias judgement** | | **High** | | |  |
| **Bias in selection of the reported result** | 5.1 Were the data that produced this result analysed in accordance with a pre-specified analysis plan that was finalized before unblinded outcome data were available for analysis? | | NI | | | No mention of protocol. |
|  | 5.2 ... multiple eligible outcome measurements (e.g. scales, definitions, time points) within the outcome domain? | | NI | | | No mention of protocol. |
|  | 5.3 ... multiple eligible analyses of the data? | | NI | | | No mention of protocol. |
|  | **Risk of bias judgement** | | **Some concerns** | | |  |
| **Overall bias** | **Risk of bias judgement** | | **High** | | |  |

Abbreviations: Y = yes, PY = probably yes, PN = probably no, N = no, NI = no information, NA = not applicable

| **Outcome** | GI | **Results** | | SMD, -1.91 | **Weight** | 1 |
| --- | --- | --- | --- | --- | --- | --- |
| **Domain** | **Signalling question** | | **Response** | | | **Comments** |
| **Bias arising from the randomization process** | 1.1 Was the allocation sequence random? | | NI | | | The only information about randomization methods is a statement that the   study is randomized. |
|  | 1.2 Was the allocation sequence concealed until participants were enrolled and assigned to interventions? | | NI | | |  |
|  | 1.3 Did baseline differences between intervention groups suggest a problem with the randomization process? | | N | | | There were no statistically significant differences in oral health-related behavior characteristics and general characteristics of the participants(p > 0.05). |
|  | **Risk of bias judgement** | | **Some concerns** | | |  |
| **Bias due to deviations from intended interventions** | 2.1.Were participants aware of their assigned intervention during the trial? | | Y | | | The interventions are fundamentally different in delivery method, making blinding of participants and personnel impossible. |
|  | 2.2.Were carers and people delivering the interventions aware of participants' assigned intervention during the trial? | | PY | | |  |
|  | 2.3. If Y/PY/NI to 2.1 or 2.2: Were there deviations from the intended intervention that arose because of the experimental context? | | PN | | | There were no deviations from the intended intervention that arose. |
|  | 2.4 If Y/PY to 2.3: Were these deviations likely to have affected the outcome? | | NA | | |  |
|  | 2.5. If Y/PY/NI to 2.4: Were these deviations from intended intervention balanced between groups? | | NA | | |  |
|  | 2.6 Was an appropriate analysis used to estimate the effect of assignment to intervention? | | Y | | | Analysis appears to follow intention-to-treat principles, as all randomized participants who completed the study were included in final analysis. |
|  | 2.7 If N/PN/NI to 2.6: Was there potential for a substantial impact (on the result) of the failure to analyse participants in the group to which they were randomized? | | NA | | |  |
|  | **Risk of bias judgement** | | **Low** | | |  |
| **Bias due to missing outcome data** | 3.1 Were data for this outcome available for all, or nearly all, participants randomized? | | PN | | | "60 participants (30 per group)", "the final analysis included data from 28 participants in the experimental group and 23 in the control group". The dropout rate is 15%. |
|  | 3.2 If N/PN/NI to 3.1: Is there evidence that result was not biased by missing outcome data? | | PN | | | No sensitivity analyses or methods to correct bias were mentioned. |
|  | 3.3 If N/PN to 3.2: Could missingness in the outcome depend on its true value? | | PN | | | Participants withdrew because they were unwilling to continue. |
|  | 3.4 If Y/PY/NI to 3.3: Is it likely that missingness in the outcome depended on its true value? | | NA | | |  |
|  | **Risk of bias judgement** | | **Low** | | |  |
| **Bias in measurement of the outcome** | 4.1 Was the method of measuring the outcome inappropriate? | | N | | | Löe & Silness Index is a validated, widely used measure for gingival inflammation. |
|  | 4.2 Could measurement or ascertainment of the outcome have differed between intervention groups? | | PN | | | Two different researchers performed exams independently, likely same method for both groups. |
|  | 4.3 Were outcome assessors aware of the intervention received by study participants? | | PY | | | Blinding of assessors not mentioned; likely aware due to app training sessions. |
|  | 4.4 If Y/PY/NI to 4.3: Could assessment of the outcome have been influenced by knowledge of intervention received? | | PY | | | GI involves clinical judgment; awareness of group assignment could influence scoring. |
|  | 4.5 If Y/PY/NI to 4.4: Is it likely that assessment of the outcome was influenced by knowledge of intervention received? | | PY | | |  |
|  | **Risk of bias judgement** | | **High** | | |  |
| **Bias in selection of the reported result** | 5.1 Were the data that produced this result analysed in accordance with a pre-specified analysis plan that was finalized before unblinded outcome data were available for analysis? | | NI | | | No mention of protocol. |
|  | 5.2 ... multiple eligible outcome measurements (e.g. scales, definitions, time points) within the outcome domain? | | NI | | | No mention of protocol. |
|  | 5.3 ... multiple eligible analyses of the data? | | NI | | | No mention of protocol. |
|  | **Risk of bias judgement** | | **Some concerns** | | |  |
| **Overall bias** | **Risk of bias judgement** | | **High** | | |  |

Abbreviations: Y = yes, PY = probably yes, PN = probably no, N = no, NI = no information, NA = not applicable

| **Outcome** | O'Leary index | **Results** | | SMD, -1.36 | **Weight** | 1 |
| --- | --- | --- | --- | --- | --- | --- |
| **Domain** | **Signalling question** | | **Response** | | | **Comments** |
| **Bias arising from the randomization process** | 1.1 Was the allocation sequence random? | | NI | | | The only information about randomization methods is a statement that the   study is randomized. |
|  | 1.2 Was the allocation sequence concealed until participants were enrolled and assigned to interventions? | | NI | | |  |
|  | 1.3 Did baseline differences between intervention groups suggest a problem with the randomization process? | | N | | | There were no statistically significant differences in oral health-related behavior characteristics and general characteristics of the participants(p > 0.05). |
|  | **Risk of bias judgement** | | **Some concerns** | | |  |
| **Bias due to deviations from intended interventions** | 2.1.Were participants aware of their assigned intervention during the trial? | | Y | | | The interventions are fundamentally different in delivery method, making blinding of participants and personnel impossible. |
|  | 2.2.Were carers and people delivering the interventions aware of participants' assigned intervention during the trial? | | PY | | |  |
|  | 2.3. If Y/PY/NI to 2.1 or 2.2: Were there deviations from the intended intervention that arose because of the experimental context? | | PN | | | There were no deviations from the intended intervention that arose. |
|  | 2.4 If Y/PY to 2.3: Were these deviations likely to have affected the outcome? | | NA | | |  |
|  | 2.5. If Y/PY/NI to 2.4: Were these deviations from intended intervention balanced between groups? | | NA | | |  |
|  | 2.6 Was an appropriate analysis used to estimate the effect of assignment to intervention? | | Y | | | Analysis appears to follow intention-to-treat principles, as all randomized participants who completed the study were included in final analysis. |
|  | 2.7 If N/PN/NI to 2.6: Was there potential for a substantial impact (on the result) of the failure to analyse participants in the group to which they were randomized? | | NA | | |  |
|  | **Risk of bias judgement** | | **Low** | | |  |
| **Bias due to missing outcome data** | 3.1 Were data for this outcome available for all, or nearly all, participants randomized? | | PN | | | "60 participants (30 per group)", "the final analysis included data from 28 participants in the experimental group and 23 in the control group". The dropout rate is 15%. |
|  | 3.2 If N/PN/NI to 3.1: Is there evidence that result was not biased by missing outcome data? | | PN | | | No sensitivity analyses or methods to correct bias were mentioned. |
|  | 3.3 If N/PN to 3.2: Could missingness in the outcome depend on its true value? | | PN | | | Participants withdrew because they were unwilling to continue. |
|  | 3.4 If Y/PY/NI to 3.3: Is it likely that missingness in the outcome depended on its true value? | | NA | | |  |
|  | **Risk of bias judgement** | | **Low** | | |  |
| **Bias in measurement of the outcome** | 4.1 Was the method of measuring the outcome inappropriate? | | N | | | O’Leary index is a validated plaque index. |
|  | 4.2 Could measurement or ascertainment of the outcome have differed between intervention groups? | | PN | | | Two different researchers performed exams independently, likely same method for both groups. |
|  | 4.3 Were outcome assessors aware of the intervention received by study participants? | | PY | | | Blinding of assessors not mentioned; likely aware due to app training sessions. |
|  | 4.4 If Y/PY/NI to 4.3: Could assessment of the outcome have been influenced by knowledge of intervention received? | | PN | | | Plaque index is objective; less prone to assessor bias. |
|  | 4.5 If Y/PY/NI to 4.4: Is it likely that assessment of the outcome was influenced by knowledge of intervention received? | | NA | | |  |
|  | **Risk of bias judgement** | | **Low** | | |  |
| **Bias in selection of the reported result** | 5.1 Were the data that produced this result analysed in accordance with a pre-specified analysis plan that was finalized before unblinded outcome data were available for analysis? | | NI | | | No mention of protocol. |
|  | 5.2 ... multiple eligible outcome measurements (e.g. scales, definitions, time points) within the outcome domain? | | NI | | | No mention of protocol. |
|  | 5.3 ... multiple eligible analyses of the data? | | NI | | | No mention of protocol. |
|  | **Risk of bias judgement** | | **Some concerns** | | |  |
| **Overall bias** | **Risk of bias judgement** | | **Some concerns** | | |  |

Abbreviations: Y = yes, PY = probably yes, PN = probably no, N = no, NI = no information, NA = not applicable

| **Outcome** | Winkel tongue coating index | **Results** | SMD, -1.36 | | **Weight** | 1 |
| --- | --- | --- | --- | --- | --- | --- |
| **Domain** | **Signalling question** | | | **Response** | | **Comments** |
| **Bias arising from the randomization process** | 1.1 Was the allocation sequence random? | | | NI | | The only information about randomization methods is a statement that the   study is randomized. |
|  | 1.2 Was the allocation sequence concealed until participants were enrolled and assigned to interventions? | | | NI | |  |
|  | 1.3 Did baseline differences between intervention groups suggest a problem with the randomization process? | | | N | | There were no statistically significant differences in oral health-related behavior characteristics and general characteristics of the participants(p > 0.05). |
|  | **Risk of bias judgement** | | | **Some concerns** | |  |
| **Bias due to deviations from intended interventions** | 2.1.Were participants aware of their assigned intervention during the trial? | | | Y | | The interventions are fundamentally different in delivery method, making blinding of participants and personnel impossible. |
|  | 2.2.Were carers and people delivering the interventions aware of participants' assigned intervention during the trial? | | | PY | |  |
|  | 2.3. If Y/PY/NI to 2.1 or 2.2: Were there deviations from the intended intervention that arose because of the experimental context? | | | PN | | There were no deviations from the intended intervention that arose. |
|  | 2.4 If Y/PY to 2.3: Were these deviations likely to have affected the outcome? | | | NA | |  |
|  | 2.5. If Y/PY/NI to 2.4: Were these deviations from intended intervention balanced between groups? | | | NA | |  |
|  | 2.6 Was an appropriate analysis used to estimate the effect of assignment to intervention? | | | Y | | Analysis appears to follow intention-to-treat principles, as all randomized participants who completed the study were included in final analysis. |
|  | 2.7 If N/PN/NI to 2.6: Was there potential for a substantial impact (on the result) of the failure to analyse participants in the group to which they were randomized? | | | NA | |  |
|  | **Risk of bias judgement** | | | **Low** | |  |
| **Bias due to missing outcome data** | 3.1 Were data for this outcome available for all, or nearly all, participants randomized? | | | PN | | "60 participants (30 per group)", "the final analysis included data from 28 participants in the experimental group and 23 in the control group". The dropout rate is 15%. |
|  | 3.2 If N/PN/NI to 3.1: Is there evidence that result was not biased by missing outcome data? | | | PN | | No sensitivity analyses or methods to correct bias were mentioned. |
|  | 3.3 If N/PN to 3.2: Could missingness in the outcome depend on its true value? | | | PN | | Participants withdrew because they were unwilling to continue. |
|  | 3.4 If Y/PY/NI to 3.3: Is it likely that missingness in the outcome depended on its true value? | | | NA | |  |
|  | **Risk of bias judgement** | | | **Low** | |  |
| **Bias in measurement of the outcome** | 4.1 Was the method of measuring the outcome inappropriate? | | | N | | Winkel tongue coating index is a validated method. |
|  | 4.2 Could measurement or ascertainment of the outcome have differed between intervention groups? | | | PN | | Two different researchers performed exams independently, likely same method for both groups. |
|  | 4.3 Were outcome assessors aware of the intervention received by study participants? | | | PY | | Blinding of assessors not mentioned; likely aware due to app training sessions. |
|  | 4.4 If Y/PY/NI to 4.3: Could assessment of the outcome have been influenced by knowledge of intervention received? | | | PN | | Winkel tongue coating index is objective; less prone to assessor bias. |
|  | 4.5 If Y/PY/NI to 4.4: Is it likely that assessment of the outcome was influenced by knowledge of intervention received? | | | NA | |  |
|  | **Risk of bias judgement** | | | **Low** | |  |
| **Bias in selection of the reported result** | 5.1 Were the data that produced this result analysed in accordance with a pre-specified analysis plan that was finalized before unblinded outcome data were available for analysis? | | | NI | | No mention of protocol. |
|  | 5.2 ... multiple eligible outcome measurements (e.g. scales, definitions, time points) within the outcome domain? | | | NI | | No mention of protocol. |
|  | 5.3 ... multiple eligible analyses of the data? | | | NI | | No mention of protocol. |
|  | **Risk of bias judgement** | | | **Some concerns** | |  |
| **Overall bias** | **Risk of bias judgement** | | | **Some concerns** | |  |

Abbreviations: Y = yes, PY = probably yes, PN = probably no, N = no, NI = no information, NA = not applicable

| **Outcome** | Anterior tongue strength | **Results** | SMD, -0.27 | | **Weight** | 1 |
| --- | --- | --- | --- | --- | --- | --- |
| **Domain** | **Signalling question** | | | **Response** | | **Comments** |
| **Bias arising from the randomization process** | 1.1 Was the allocation sequence random? | | | NI | | The only information about randomization methods is a statement that the   study is randomized. |
|  | 1.2 Was the allocation sequence concealed until participants were enrolled and assigned to interventions? | | | NI | |  |
|  | 1.3 Did baseline differences between intervention groups suggest a problem with the randomization process? | | | N | | There were no statistically significant differences in oral health-related behavior characteristics and general characteristics of the participants(p > 0.05). |
|  | **Risk of bias judgement** | | | **Some concerns** | |  |
| **Bias due to deviations from intended interventions** | 2.1.Were participants aware of their assigned intervention during the trial? | | | Y | | The interventions are fundamentally different in delivery method, making blinding of participants and personnel impossible. |
|  | 2.2.Were carers and people delivering the interventions aware of participants' assigned intervention during the trial? | | | PY | |  |
|  | 2.3. If Y/PY/NI to 2.1 or 2.2: Were there deviations from the intended intervention that arose because of the experimental context? | | | PN | | There were no deviations from the intended intervention that arose. |
|  | 2.4 If Y/PY to 2.3: Were these deviations likely to have affected the outcome? | | | NA | |  |
|  | 2.5. If Y/PY/NI to 2.4: Were these deviations from intended intervention balanced between groups? | | | NA | |  |
|  | 2.6 Was an appropriate analysis used to estimate the effect of assignment to intervention? | | | Y | | Analysis appears to follow intention-to-treat principles, as all randomized participants who completed the study were included in final analysis. |
|  | 2.7 If N/PN/NI to 2.6: Was there potential for a substantial impact (on the result) of the failure to analyse participants in the group to which they were randomized? | | | NA | |  |
|  | **Risk of bias judgement** | | | **Low** | |  |
| **Bias due to missing outcome data** | 3.1 Were data for this outcome available for all, or nearly all, participants randomized? | | | PN | | "60 participants (30 per group)", "the final analysis included data from 28 participants in the experimental group and 23 in the control group". The dropout rate is 15%. |
|  | 3.2 If N/PN/NI to 3.1: Is there evidence that result was not biased by missing outcome data? | | | PN | | No sensitivity analyses or methods to correct bias were mentioned. |
|  | 3.3 If N/PN to 3.2: Could missingness in the outcome depend on its true value? | | | PN | | Participants withdrew because they were unwilling to continue. |
|  | 3.4 If Y/PY/NI to 3.3: Is it likely that missingness in the outcome depended on its true value? | | | NA | |  |
|  | **Risk of bias judgement** | | | **Low** | |  |
| **Bias in measurement of the outcome** | 4.1 Was the method of measuring the outcome inappropriate? | | | N | | Iowa Oral Performance Instrument (IOPI) is valid for tongue strength. |
|  | 4.2 Could measurement or ascertainment of the outcome have differed between intervention groups? | | | PN | | Same instrument used for both groups, same timing. |
|  | 4.3 Were outcome assessors aware of the intervention received by study participants? | | | PY | | Not explicitly stated, but likely that assessors were aware due to unblinded study design. |
|  | 4.4 If Y/PY/NI to 4.3: Could assessment of the outcome have been influenced by knowledge of intervention received? | | | PN | | IOPI is an objective, quantitative measure unlikely to be influenced by assessor judgement. |
|  | 4.5 If Y/PY/NI to 4.4: Is it likely that assessment of the outcome was influenced by knowledge of intervention received? | | | NA | |  |
|  | **Risk of bias judgement** | | | **Low** | |  |
| **Bias in selection of the reported result** | 5.1 Were the data that produced this result analysed in accordance with a pre-specified analysis plan that was finalized before unblinded outcome data were available for analysis? | | | NI | | No mention of protocol. |
|  | 5.2 ... multiple eligible outcome measurements (e.g. scales, definitions, time points) within the outcome domain? | | | NI | | No mention of protocol. |
|  | 5.3 ... multiple eligible analyses of the data? | | | NI | | No mention of protocol. |
|  | **Risk of bias judgement** | | | **Some concerns** | |  |
| **Overall bias** | **Risk of bias judgement** | | | **Some concerns** | |  |

Abbreviations: Y = yes, PY = probably yes, PN = probably no, N = no, NI = no information, NA = not applicable

| **Outcome** | Cheek strength | **Results** | SMD, 0.03 | **Weight** | 1 |
| --- | --- | --- | --- | --- | --- |
| **Domain** | **Signalling question** | | | **Response** | **Comments** |
| **Bias arising from the randomization process** | 1.1 Was the allocation sequence random? | | | NI | The only information about randomization methods is a statement that the   study is randomized. |
|  | 1.2 Was the allocation sequence concealed until participants were enrolled and assigned to interventions? | | | NI |  |
|  | 1.3 Did baseline differences between intervention groups suggest a problem with the randomization process? | | | N | There were no statistically significant differences in oral health-related behavior characteristics and general characteristics of the participants(p > 0.05). |
|  | **Risk of bias judgement** | | | **Some concerns** |  |
| **Bias due to deviations from intended interventions** | 2.1.Were participants aware of their assigned intervention during the trial? | | | Y | The interventions are fundamentally different in delivery method, making blinding of participants and personnel impossible. |
|  | 2.2.Were carers and people delivering the interventions aware of participants' assigned intervention during the trial? | | | PY |  |
|  | 2.3. If Y/PY/NI to 2.1 or 2.2: Were there deviations from the intended intervention that arose because of the experimental context? | | | PN | There were no deviations from the intended intervention that arose. |
|  | 2.4 If Y/PY to 2.3: Were these deviations likely to have affected the outcome? | | | NA |  |
|  | 2.5. If Y/PY/NI to 2.4: Were these deviations from intended intervention balanced between groups? | | | NA |  |
|  | 2.6 Was an appropriate analysis used to estimate the effect of assignment to intervention? | | | Y | Analysis appears to follow intention-to-treat principles, as all randomized participants who completed the study were included in final analysis. |
|  | 2.7 If N/PN/NI to 2.6: Was there potential for a substantial impact (on the result) of the failure to analyse participants in the group to which they were randomized? | | | NA |  |
|  | **Risk of bias judgement** | | | **Low** |  |
| **Bias due to missing outcome data** | 3.1 Were data for this outcome available for all, or nearly all, participants randomized? | | | PN | "60 participants (30 per group)", "the final analysis included data from 28 participants in the experimental group and 23 in the control group". The dropout rate is 15%. |
|  | 3.2 If N/PN/NI to 3.1: Is there evidence that result was not biased by missing outcome data? | | | PN | No sensitivity analyses or methods to correct bias were mentioned. |
|  | 3.3 If N/PN to 3.2: Could missingness in the outcome depend on its true value? | | | PN | Participants withdrew because they were unwilling to continue. |
|  | 3.4 If Y/PY/NI to 3.3: Is it likely that missingness in the outcome depended on its true value? | | | NA |  |
|  | **Risk of bias judgement** | | | **Low** |  |
| **Bias in measurement of the outcome** | 4.1 Was the method of measuring the outcome inappropriate? | | | N | Iowa Oral Performance Instrument (IOPI) is valid for Cheek strength. |
|  | 4.2 Could measurement or ascertainment of the outcome have differed between intervention groups? | | | PN | Same instrument used for both groups, same timing. |
|  | 4.3 Were outcome assessors aware of the intervention received by study participants? | | | PY | Not explicitly stated, but likely that assessors were aware due to unblinded study design. |
|  | 4.4 If Y/PY/NI to 4.3: Could assessment of the outcome have been influenced by knowledge of intervention received? | | | PN | IOPI is an objective, quantitative measure unlikely to be influenced by assessor judgement. |
|  | 4.5 If Y/PY/NI to 4.4: Is it likely that assessment of the outcome was influenced by knowledge of intervention received? | | | NA |  |
|  | **Risk of bias judgement** | | | **Low** |  |
| **Bias in selection of the reported result** | 5.1 Were the data that produced this result analysed in accordance with a pre-specified analysis plan that was finalized before unblinded outcome data were available for analysis? | | | NI | No mention of protocol. |
|  | 5.2 ... multiple eligible outcome measurements (e.g. scales, definitions, time points) within the outcome domain? | | | NI | No mention of protocol. |
|  | 5.3 ... multiple eligible analyses of the data? | | | NI | No mention of protocol. |
|  | **Risk of bias judgement** | | | **Some concerns** |  |
| **Overall bias** | **Risk of bias judgement** | | | **Some concerns** |  |

Abbreviations: Y = yes, PY = probably yes, PN = probably no, N = no, NI = no information, NA = not applicable

## Manan 2025

| **Outcome** | Oral healthcare-related knowledge | **Results** | p > 0.05 | **Weight** | 1 |
| --- | --- | --- | --- | --- | --- |
| **Domain** | **Signalling question** | | **Response** | | **Comments** |
| **Bias arising from the randomization process** | 1.1 Was the allocation sequence random? | | Y | | “Simple random sampling or method of chances, in which each member of the population had an equal chance to be chosen as the sample, was applied.” No mention of allocation concealment. |
|  | 1.2 Was the allocation sequence concealed until participants were enrolled and assigned to interventions? | | NI | |  |
|  | 1.3 Did baseline differences between intervention groups suggest a problem with the randomization process? | | N | | Table 1 shows baseline characteristics were comparable between groups, with no indication of substantial imbalance. |
|  | **Risk of bias judgement** | | **Some concerns** | |  |
| **Bias due to deviations from intended interventions** | 2.1.Were participants aware of their assigned intervention during the trial? | | PY | | The interventions are fundamentally different in delivery method, making blinding of participants and personnel impossible. |
|  | 2.2.Were carers and people delivering the interventions aware of participants' assigned intervention during the trial? | | PY | |  |
|  | 2.3. If Y/PY/NI to 2.1 or 2.2: Were there deviations from the intended intervention that arose because of the experimental context? | | PN | | There were no deviations from the intended intervention that arose. |
|  | 2.4 If Y/PY to 2.3: Were these deviations likely to have affected the outcome? | | NA | |  |
|  | 2.5. If Y/PY/NI to 2.4: Were these deviations from intended intervention balanced between groups? | | NA | |  |
|  | 2.6 Was an appropriate analysis used to estimate the effect of assignment to intervention? | | Y | | Data were analyzed using a statistical software pro­gram (IBM SPSS Statistics, v27; IBM Corp). The Shapiro-Wilk test was used to assess the normality distribution of the data. The Wilcoxon Signed-Rank test was used to compare pre-intervention and post­ intervention scores for each item in each domain of knowledge and level of awareness in each group. The Mann-Whitney U test was used to compare level im­provements between the 2 methods. Paired t tests compared the total scores for each domain of knowledge and level of awareness between pre- and postinterven­tions, whereas independent t tests were used to compare knowledge increments between the 2 groups (α=.05). |
|  | 2.7 If N/PN/NI to 2.6: Was there potential for a substantial impact (on the result) of the failure to analyse participants in the group to which they were randomized? | | NA | |  |
|  | **Risk of bias judgement** | | **Low** | |  |
| **Bias due to missing outcome data** | 3.1 Were data for this outcome available for all, or nearly all, participants randomized? | | Y | | 62 participants were recruited, all completed pre- and post-questionnaires |
|  | 3.2 If N/PN/NI to 3.1: Is there evidence that result was not biased by missing outcome data? | | NA | |  |
|  | 3.3 If N/PN to 3.2: Could missingness in the outcome depend on its true value? | | NA | |  |
|  | 3.4 If Y/PY/NI to 3.3: Is it likely that missingness in the outcome depended on its true value? | | NA | |  |
|  | **Risk of bias judgement** | | **Low** | |  |
| **Bias in measurement of the outcome** | 4.1 Was the method of measuring the outcome inappropriate? | | N | | The outcome was predetermined. |
|  | 4.2 Could measurement or ascertainment of the outcome have differed between intervention groups? | | PN | |  |
|  | 4.3 Were outcome assessors aware of the intervention received by study participants? | | N | | Mann-Whitney U test was used to compare level im­provements between the 2 methods. Paired t tests compared the total scores for each domain of knowledge and level of awareness between pre- and postinterven­tions, whereas independent t tests were used to compare knowledge increments between the 2 groups (α=.05). |
|  | 4.4 If Y/PY/NI to 4.3: Could assessment of the outcome have been influenced by knowledge of intervention received? | | NA | |  |
|  | 4.5 If Y/PY/NI to 4.4: Is it likely that assessment of the outcome was influenced by knowledge of intervention received? | | NA | |  |
|  | **Risk of bias judgement** | | **Low** | |  |
| **Bias in selection of the reported result** | 5.1 Were the data that produced this result analysed in accordance with a pre-specified analysis plan that was finalized before unblinded outcome data were available for analysis? | | NI | | No mention of protocol. |
|  | 5.2 ... multiple eligible outcome measurements (e.g. scales, definitions, time points) within the outcome domain? | | NI | | No mention of protocol. |
|  | 5.3 ... multiple eligible analyses of the data? | | NI | | No mention of protocol. |
|  | **Risk of bias judgement** | | **Some concerns** | |  |
| **Overall bias** | **Risk of bias judgement** | | **Some concerns** | |  |

Abbreviations: Y = yes, PY = probably yes, PN = probably no, N = no, NI = no information, NA = not applicable

## Ng 2021

| **Outcome** | GI | **Results** | SMD, -1.04 | **Weight** | 1 |
| --- | --- | --- | --- | --- | --- |
| **Domain** | **Signalling question** | | | **Response** | **Comments** |
| **Bias arising from the randomization process** | 1.1 Was the allocation sequence random? | | | Y | A random number generator (https://stattrek.com/statistics/random-number-generator.aspx) was used to assign unique identification numbers to the participants, who were then allocated equally into the PWA and control groups via a random sequence generator software (https://www.graphpad.com/quickcalcs/randomize1/). The participants’ allocation group was concealed in sealed brown envelopes labelled with their identification numbers by a research assistant who was not involved in the data collection nor analysis. |
|  | 1.2 Was the allocation sequence concealed until participants were enrolled and assigned to interventions? | | | Y |  |
|  | 1.3 Did baseline differences between intervention groups suggest a problem with the randomization process? | | | N | The results of the two-way mixed ANOVA showed no significant difference in mean knowledge scores (F = 0.007, p = 0.934) between the PWA group and the control group at T0, T1 and T2. |
|  | **Risk of bias judgement** | | | **Low** |  |
| **Bias due to deviations from intended interventions** | 2.1.Were participants aware of their assigned intervention during the trial? | | | PY | Due to the nature of the intervention, blinding of the participants was infeasible. |
|  | 2.2.Were carers and people delivering the interventions aware of participants' assigned intervention during the trial? | | | PY |  |
|  | 2.3. If Y/PY/NI to 2.1 or 2.2: Were there deviations from the intended intervention that arose because of the experimental context? | | | N | There were no deviations from the intended intervention that arose. |
|  | 2.4 If Y/PY to 2.3: Were these deviations likely to have affected the outcome? | | | NA |  |
|  | 2.5. If Y/PY/NI to 2.4: Were these deviations from intended intervention balanced between groups? | | | NA |  |
|  | 2.6 Was an appropriate analysis used to estimate the effect of assignment to intervention? | | | PY | ITT analysis. |
|  | 2.7 If N/PN/NI to 2.6: Was there potential for a substantial impact (on the result) of the failure to analyse participants in the group to which they were randomized? | | | NA |  |
|  | **Risk of bias judgement** | | | **Low** |  |
| **Bias due to missing outcome data** | 3.1 Were data for this outcome available for all, or nearly all, participants randomized? | | | Y | Dropout rate is 5.8%. |
|  | 3.2 If N/PN/NI to 3.1: Is there evidence that result was not biased by missing outcome data? | | | NA |  |
|  | 3.3 If N/PN to 3.2: Could missingness in the outcome depend on its true value? | | | NA |  |
|  | 3.4 If Y/PY/NI to 3.3: Is it likely that missingness in the outcome depended on its true value? | | | NA |  |
|  | **Risk of bias judgement** | | | **Low** |  |
| **Bias in measurement of the outcome** | 4.1 Was the method of measuring the outcome inappropriate? | | | N | GI is a validated index; examiner calibrated (kappa = 0.826). |
|  | 4.2 Could measurement or ascertainment of the outcome have differed between intervention groups? | | | N | Same examiner, same method, same time points. |
|  | 4.3 Were outcome assessors aware of the intervention received by study participants? | | | N | The study was single-blind, whereby the participant’s allocation was kept anonymous from the outcome assessor. |
|  | 4.4 If Y/PY/NI to 4.3: Could assessment of the outcome have been influenced by knowledge of intervention received? | | | NA |  |
|  | 4.5 If Y/PY/NI to 4.4: Is it likely that assessment of the outcome was influenced by knowledge of intervention received? | | | NA |  |
|  | **Risk of bias judgement** | | | **Low** |  |
| **Bias in selection of the reported result** | 5.1 Were the data that produced this result analysed in accordance with a pre-specified analysis plan that was finalized before unblinded outcome data were available for analysis? | | | Y | The data collected were explored and analysed on SPSS (version 26, IBM). The participants’ demographic characteristics were summarised using descriptive statistics. The effect of PWA on the knowledge scores compared with that of the conventional method of education was analysed using two-way mixed ANOVA. For ordinal data, differences in the PI, GI and DP scores between the PWA group and the control group at all three visits were determined using the Mann–Whitney U test. Friedman test was conducted to analyse the changes in baseline PI, GI, and DP scores within each group after exposure to education. The level of significance was set at p < 0.05. |
|  | 5.2 ... multiple eligible outcome measurements (e.g. scales, definitions, time points) within the outcome domain? | | | PN | All outcomes mentioned in the methods are reported in results with complete statistical data. |
|  | 5.3 ... multiple eligible analyses of the data? | | | PN | All outcomes mentioned in the methods are reported in results with complete statistical data. |
|  | **Risk of bias judgement** | | | **Low** |  |
| **Overall bias** | **Risk of bias judgement** | | | **Low** |  |

Abbreviations: Y = yes, PY = probably yes, PN = probably no, N = no, NI = no information, NA = not applicable

## Nishi 2019

| **Outcome** | chance of avoiding new cavities | **Results** | Personalised | | **Weight** | 1 |
| --- | --- | --- | --- | --- | --- | --- |
| **Domain** | **Signalling question** | | | **Response** | | **Comments** |
| **Bias arising from the randomization process** | 1.1 Was the allocation sequence random? | | | Y | | The statistician generated random numbers for stratified and blocked randomisation using Proc Surveyselect, SAS, Version 9.4 (SAS Institute Inc., Cary, NC). Details on the allocation concealment is presented in Additional file 2. |
|  | 1.2 Was the allocation sequence concealed until participants were enrolled and assigned to interventions? | | | Y | |  |
|  | 1.3 Did baseline differences between intervention groups suggest a problem with the randomization process? | | | N | | Table 1 shows baseline characteristics were generally balanced between groups. Minor differences appear compatible with chance. |
|  | **Risk of bias judgement** | | | **Low** | |  |
| **Bias due to deviations from intended interventions** | 2.1.Were participants aware of their assigned intervention during the trial? | | | Y | | Participants received different letters (personalized vs. non-personalized) and different text messages, so they would have known their group assignment. The staff sending letters (LF) and the programmer sending texts knew the allocation. |
|  | 2.2.Were carers and people delivering the interventions aware of participants' assigned intervention during the trial? | | | Y | |  |
|  | 2.3. If Y/PY/NI to 2.1 or 2.2: Were there deviations from the intended intervention that arose because of the experimental context? | | | PN | | There were no deviations from the intended intervention that arose. |
|  | 2.4 If Y/PY to 2.3: Were these deviations likely to have affected the outcome? | | | NA | |  |
|  | 2.5. If Y/PY/NI to 2.4: Were these deviations from intended intervention balanced between groups? | | | NA | |  |
|  | 2.6 Was an appropriate analysis used to estimate the effect of assignment to intervention? | | | Y | | Both ITT and per-protocol analyses were conducted. |
|  | 2.7 If N/PN/NI to 2.6: Was there potential for a substantial impact (on the result) of the failure to analyse participants in the group to which they were randomized? | | | NA | |  |
|  | **Risk of bias judgement** | | | **Low** | |  |
| **Bias due to missing outcome data** | 3.1 Were data for this outcome available for all, or nearly all, participants randomized? | | | PN | | The dropout rate was 35.1%(60/171) |
|  | 3.2 If N/PN/NI to 3.1: Is there evidence that result was not biased by missing outcome data? | | | N | | No sensitivity analyses or methods to correct for bias were reported. |
|  | 3.3 If N/PN to 3.2: Could missingness in the outcome depend on its true value? | | | PN | | 54individuals did not attend the clinical examination, and 6 did not return their three-day dietary diaries. The component loss-to-follow-up rate was balanced. |
|  | 3.4 If Y/PY/NI to 3.3: Is it likely that missingness in the outcome depended on its true value? | | | NA | |  |
|  | **Risk of bias judgement** | | | **Low** | |  |
| **Bias in measurement of the outcome** | 4.1 Was the method of measuring the outcome inappropriate? | | | N | | Cariogram is a validated caries risk assessment tool. |
|  | 4.2 Could measurement or ascertainment of the outcome have differed between intervention groups? | | | N | | Same clinical examination, CRT tests, food diaries, and questionnaires were used for both groups. |
|  | 4.3 Were outcome assessors aware of the intervention received by study participants? | | | N | | The study was single-blinded (assessor-blinded). Dentists performing examinations were blinded. |
|  | 4.4 If Y/PY/NI to 4.3: Could assessment of the outcome have been influenced by knowledge of intervention received? | | | NA | |  |
|  | 4.5 If Y/PY/NI to 4.4: Is it likely that assessment of the outcome was influenced by knowledge of intervention received? | | | NA | |  |
|  | **Risk of bias judgement** | | | **Low** | |  |
| **Bias in selection of the reported result** | 5.1 Were the data that produced this result analysed in accordance with a pre-specified analysis plan that was finalized before unblinded outcome data were available for analysis? | | | Y | | The protocol was registered (UMIN000027253), and primary/secondary outcomes were pre-specified. |
|  | 5.2 ... multiple eligible outcome measurements (e.g. scales, definitions, time points) within the outcome domain? | | | N | | Only “chance of avoiding new cavities” from Cariogram was used as primary outcome. |
|  | 5.3 ... multiple eligible analyses of the data? | | | N | | All outcomes mentioned in methods are reported in results with complete statistical data. |
|  | **Risk of bias judgement** | | | **Low** | |  |
| **Overall bias** | **Risk of bias judgement** | | | **Low** | |  |

Abbreviations: Y = yes, PY = probably yes, PN = probably no, N = no, NI = no information, NA = not applicable

## Romalee 2023

| **Outcome** | oral healthcare-related knowledge | **Results** | p>.05 | **Weight** | 1 |
| --- | --- | --- | --- | --- | --- |
| **Domain** | **Signalling question** | | | **Response** | **Comments** |
| **Bias arising from the randomization process** | 1.1 Was the allocation sequence random? | | | Y | A random number table was employed by a principal investigator for   random selection and allocation in a 3-arm, parallel, open-label randomized controlled trial. The paper does not describe allocation concealment methods. |
|  | 1.2 Was the allocation sequence concealed until participants were enrolled and assigned to interventions? | | | NI |  |
|  | 1.3 Did baseline differences between intervention groups suggest a problem with the randomization process? | | | N | Participant characteristics did not significantly differ between the groups, except for alcohol consumption (p = .027)However, no significant correlations were found between alcohol consumption and the outcome variables. In addition, no statistically significant association regarding characteristics and outcomes at T3 was detected. |
|  | **Risk of bias judgement** | | | **Some concerns** |  |
| **Bias due to deviations from intended interventions** | 2.1.Were participants aware of their assigned intervention during the trial? | | | Y | Due to the nature of the intervention, blinding of the participants was infeasible. The interventions (lecture vs MAR) were fundamentally different. |
|  | 2.2.Were carers and people delivering the interventions aware of participants' assigned intervention during the trial? | | | Y |  |
|  | 2.3. If Y/PY/NI to 2.1 or 2.2: Were there deviations from the intended intervention that arose because of the experimental context? | | | PN | There were no deviations from the intended intervention that arose. |
|  | 2.4 If Y/PY to 2.3: Were these deviations likely to have affected the outcome? | | | NA |  |
|  | 2.5. If Y/PY/NI to 2.4: Were these deviations from intended intervention balanced between groups? | | | NA |  |
|  | 2.6 Was an appropriate analysis used to estimate the effect of assignment to intervention? | | | PY | The analysis appears to follow intention-to-treat principles; all randomized participants who completed follow-up were analyzed in their original groups. |
|  | 2.7 If N/PN/NI to 2.6: Was there potential for a substantial impact (on the result) of the failure to analyse participants in the group to which they were randomized? | | | NA |  |
|  | **Risk of bias judgement** | | | **Low** |  |
| **Bias due to missing outcome data** | 3.1 Were data for this outcome available for all, or nearly all, participants randomized? | | | N | A total of 75 older adults. Data from 61 participants were analyzed. Dropout rate was 19%. |
|  | 3.2 If N/PN/NI to 3.1: Is there evidence that result was not biased by missing outcome data? | | | PN | No sensitivity analysis or methods to correct for bias were reported. |
|  | 3.3 If N/PN to 3.2: Could missingness in the outcome depend on its true value? | | | NI | No details on reasons for dropout; cannot judge likelihood. |
|  | 3.4 If Y/PY/NI to 3.3: Is it likely that missingness in the outcome depended on its true value? | | | NI |  |
|  | **Risk of bias judgement** | | | **High** |  |
| **Bias in measurement of the outcome** | 4.1 Was the method of measuring the outcome inappropriate? | | | N | Knowledge was measured via a validated questionnaire (15 items, K-R20 = 0.69), which is appropriate. |
|  | 4.2 Could measurement or ascertainment of the outcome have differed between intervention groups? | | | N | Same questionnaire was used for all groups at same time points. |
|  | 4.3 Were outcome assessors aware of the intervention received by study participants? | | | Y | Participants self-reported knowledge, and they were aware of their intervention. |
|  | 4.4 If Y/PY/NI to 4.3: Could assessment of the outcome have been influenced by knowledge of intervention received? | | | PY | Participant-reported outcomes can be influenced by knowledge of group assignment. |
|  | 4.5 If Y/PY/NI to 4.4: Is it likely that assessment of the outcome was influenced by knowledge of intervention received? | | | PY |  |
|  | **Risk of bias judgement** | | | **High** | Due to the nature of the intervention, blinding of the participants was infeasible. The outcome measurements in such a study are always self-reported. |
| **Bias in selection of the reported result** | 5.1 Were the data that produced this result analysed in accordance with a pre-specified analysis plan that was finalized before unblinded outcome data were available for analysis? | | | Y | There was a trial register entry, which provided details of the pre‐specified outcomes. |
|  | 5.2 ... multiple eligible outcome measurements (e.g. scales, definitions, time points) within the outcome domain? | | | N | The measurement methods for oral health-related knowledge are relatively limited and standardized. The paper explicitly states that a specialized 15-item questionnaire developed in its previous research was used to measure knowledge, with a Kuder-Richardson 20 reliability coefficient of 0.69. No mention was made of using alternative scales. |
|  | 5.3 ... multiple eligible analyses of the data? | | | PN | The paper provides a detailed description of the statistical methods: one-way analysis of variance (ANOVA) was used to compare score changes from T1 to T3 across the three groups, while independent samples t-tests were employed to compare score changes from T1 to T2 and from T2 to T3 between two groups. There is no indication that they attempted multiple models and then reported only the favorable results. |
|  | **Risk of bias judgement** | | | **Low** |  |
| **Overall bias** | **Risk of bias judgement** | | | **High** |  |

Abbreviations: Y = yes, PY = probably yes, PN = probably no, N = no, NI = no information, NA = not applicable

## Wanyonyi 2022

| **Outcome** | OHIP-14 | **Results** | SMD, -0.03 | | **Weight** | 1 |
| --- | --- | --- | --- | --- | --- | --- |
| **Domain** | **Signalling question** | | | **Response** | | **Comments** |
| **Bias arising from the randomization process** | 1.1 Was the allocation sequence random? | | | Y | | The research staff at the clinic randomised participants. Each consented participant had a number drawn randomly and those labelled with an odd participant number were allocated to the TMI arm, and each participant labelled with an even participant number were allocated to the leaflet arm. |
|  | 1.2 Was the allocation sequence concealed until participants were enrolled and assigned to interventions? | | | PY | |  |
|  | 1.3 Did baseline differences between intervention groups suggest a problem with the randomization process? | | | N | | There were no statistically significant differences between the two arms at baseline. |
|  | **Risk of bias judgement** | | | **Low** | |  |
| **Bias due to deviations from intended interventions** | 2.1.Were participants aware of their assigned intervention during the trial? | | | Y | | Participants were not blinded to the randomisation arm due to the nature of the intervention. |
|  | 2.2.Were carers and people delivering the interventions aware of participants' assigned intervention during the trial? | | | Y | |  |
|  | 2.3. If Y/PY/NI to 2.1 or 2.2: Were there deviations from the intended intervention that arose because of the experimental context? | | | PN | | There were no deviations from the intended intervention that arose. |
|  | 2.4 If Y/PY to 2.3: Were these deviations likely to have affected the outcome? | | | NA | |  |
|  | 2.5. If Y/PY/NI to 2.4: Were these deviations from intended intervention balanced between groups? | | | NA | |  |
|  | 2.6 Was an appropriate analysis used to estimate the effect of assignment to intervention? | | | PY | | The analysis appears to follow intention-to-treat principles for the nested study, though full 12-month outcomes are pending. |
|  | 2.7 If N/PN/NI to 2.6: Was there potential for a substantial impact (on the result) of the failure to analyse participants in the group to which they were randomized? | | | NA | |  |
|  | **Risk of bias judgement** | | | **Low** | |  |
| **Bias due to missing outcome data** | 3.1 Were data for this outcome available for all, or nearly all, participants randomized? | | | N | | Only 68 out of 150 participants responded to the nested study (45%). |
|  | 3.2 If N/PN/NI to 3.1: Is there evidence that result was not biased by missing outcome data? | | | PN | | No sensitivity analyses or methods to correct bias were mentioned. |
|  | 3.3 If N/PN to 3.2: Could missingness in the outcome depend on its true value? | | | PN | | Although only 45% responded to the nested study survey, there was a non-biased distribution of respondees between the leaflet and the text group. There were no statistically significant differences between the two arms at baseline and during the nested study. This was assessed by looking at the proportion of participants in both samples by deprivation categories, sex and baseline clinical disease risk scoring category (red, amber and green). |
|  | 3.4 If Y/PY/NI to 3.3: Is it likely that missingness in the outcome depended on its true value? | | | NA | |  |
|  | **Risk of bias judgement** | | | **Low** | |  |
| **Bias in measurement of the outcome** | 4.1 Was the method of measuring the outcome inappropriate? | | | N | | OHIP-14 is a validated oral health-related quality of life instrument. |
|  | 4.2 Could measurement or ascertainment of the outcome have differed between intervention groups? | | | N | | OHIP-14 was self-reported via survey, same for both groups. |
|  | 4.3 Were outcome assessors aware of the intervention received by study participants? | | | Y | | OHIP-14 is self-reported; participants were aware of their group. |
|  | 4.4 If Y/PY/NI to 4.3: Could assessment of the outcome have been influenced by knowledge of intervention received? | | | PY | | Knowledge of intervention could influence self-reported outcomes. |
|  | 4.5 If Y/PY/NI to 4.4: Is it likely that assessment of the outcome was influenced by knowledge of intervention received? | | | PY | |  |
|  | **Risk of bias judgement** | | | **High** | |  |
| **Bias in selection of the reported result** | 5.1 Were the data that produced this result analysed in accordance with a pre-specified analysis plan that was finalized before unblinded outcome data were available for analysis? | | | Y | | The data were analysed in accordance with a pre-specified analysis plan. |
|  | 5.2 ... multiple eligible outcome measurements (e.g. scales, definitions, time points) within the outcome domain? | | | PN | | OHIP-14 was pre-specified as an outcome in the trial registration. |
|  | 5.3 ... multiple eligible analyses of the data? | | | PN | | Only one analysis method reported for OHIP-14. |
|  | **Risk of bias judgement** | | | **Low** | |  |
| **Overall bias** | **Risk of bias judgement** | | | **High** | |  |

Abbreviations: Y = yes, PY = probably yes, PN = probably no, N = no, NI = no information, NA = not applicable
